# Tomato Spotted Wilt Virus Reprogrammes Host Glycolysis to Facilitate Proliferation by a Phase‐Separated Co‐Aggregate of Nucleocapsid Protein and Phosphoglycerate Kinase

**DOI:** 10.1111/pbi.70529

**Published:** 2026-01-06

**Authors:** Guangcheng Zu, Zhifu Xing, Jiao Li, Tangbing Yang, Huan Wu, Qiangsheng Ge, Yanju Wang, Baoan Song, Runjiang Song

**Affiliations:** ^1^ State Key Laboratory of Green Pesticide Center for R&D of Fine Chemicals of Guizhou University Guiyang China

**Keywords:** aggregate, ATP, glycolysis, nucleocapsid protein, tomato spotted wilt virus

## Abstract

Efficient viral proliferation within the host is a critical step in pathogenicity and requires adenosine triphosphate (ATP). The replication, movement and immune evasion of many plant viruses within their hosts are associated with phase separation (PS)‐derived aggregates formed by viral components. However, the host factors that drive the formation of these condensates remain largely unknown. This study provides evidence that the nucleocapsid protein (N) of tomato spotted wilt virus (TSWV) recruits the host factor phosphoglycerate kinase (NbPGK) from *Nicotiana benthamiana* to form phase‐separated condensates. This remodels the host glycolytic pathway to generate ATP, supplying energy for viral replication via ribonucleoprotein complexes and acting as a promoter to regulate the PS network, thereby facilitating condensate formation. Notably, we have developed a small‐molecule PS modulator, **F10**. By combining drug affinity‐responsive target stability, molecular docking, microscale thermophoresis and bio‐layer interferometry techniques allowed **F10**, we confirmed binding to sites Arg94, Lys192 and Gly228 on TSWV N, residues critical for maintaining NbPGK recruitment. **F10** interacts with N, liberating the hijacked host factor NbPGK, and exhibits potent antiviral activity, outperforming the commercial virucide Ningnanmycin. This study elucidates the molecular machinery underlying viral exploitation of host cellular metabolism and identifies a lead compound that is amenable to managing TSWV by targeting this process.

## 1 Introduction

As obligate intracellular parasites, viruses are entirely dependent on the host cellular system throughout their life cycle (Moulder 1985). Interactions between viral proteins and host cellular components are critical for viral entry, replication and systemic spread (Brito and Pinney 2017; Hyodo et al. 2014; Wang, Yang, et al. 2025). Recent advances have highlighted the key role of phase separation (PS) in viral pathogenesis (Wang, Luo, et al. 2025; Yin et al. 2025; Zan et al. 2025). Through multivalent interactions, viral and host proteins assemble into membrane‐less biomolecular condensates (BMCs), which facilitate the spatial concentration of the replication machinery while shielding viral components from host immune surveillance (Li et al. 2022). Fundamentally, the assembly of PS‐driven BMCs is thermodynamically regulated and does not intrinsically require energy input (Mukherjee and Schäfer 2023). However, the synthesis and turnover of constituent biomolecules, which are necessary to achieve concentration thresholds, require energy (Zhao and Poo 2021). Viruses must co‐opt host energy metabolism to achieve efficient replication (Mayer et al. 2019). Intracellular compartmentalization provided by PS creates a favourable environment for energy hijacking. Furthermore, adenosine triphosphate (ATP) acts as a multifunctional molecule beyond its role as a primary energy currency, nucleic acid building block and substrate for protein modification. It is widely recognised as a concentration‐dependent regulator of PS (Kota et al. 2024). For instance, Patel et al. (2017) suggested that ATP suppresses the condensation of purified Fused in Sarcoma (FUS) protein at physiological concentrations (5–10 mM), whereas Kang et al. (2018) showed that lower ATP concentrations can promote FUS‐PS. Given the dual nature of ATP and its central role in both energy metabolism and PS regulation, it is essential to deepen our understanding of ATP's role in modulating PS during viral proliferation to develop novel antiviral strategies.

*Tomato spotted wilt virus* (TSWV), a member of the order *Bunyavirales* and family *Tospoviridae*, is one of the most devastating plant‐infecting viruses within the genus *Orthotospovirus*, and is primarily transmitted by western flower thrips (Lv et al. 2023; Zhang et al. 2024). Since its initial identification, TSWV has been widely acknowledged as a highly destructive plant pathogen (Scholthof et al. 2011). It infects more than 1000 plant species, including members of the *Solanaceae*, *Fabaceae* and *Asteraceae* families, along with a broad spectrum of economically valuable crops. Infected plants typically exhibit symptoms such as foliar mottling, growth retardation and complete yield loss in severe cases (Huang et al. 2021; Oliver and Whitfield 2016). The virus is responsible for considerable economic damage, with annual losses estimated to approach one billion dollars, and remains a significant threat to global agricultural productivity and food security (Huang et al. 2020, 2024). Current strategies for managing TSWV primarily involve controlling insect vectors and deploying resistant crop cultivars (Nilon et al. 2021). However, the emergence and widespread dissemination of insecticide resistance within thrip populations has significantly undermined the effectiveness of chemical‐based interventions (Li et al. 2016; Nilon et al. 2021). Although the *Sw‐5b* resistance gene effectively delays disease progression, its durability remains limited due to the emergence of resistance‐breaking viral strains (Huang et al. 2022, 2024; Zhu et al. 2019). The lack of highly effective, commercially available antiviral agents impedes the management of TSWV on farmland.

The TSWV genome consists of three single‐stranded RNA segments (L, M and S) that collectively encode essential viral proteins, including the nucleocapsid protein (N), the movement protein (NSm) and the RNA‐dependent RNA polymerase (RdRp) (Tsompana et al. 2005). Among these, the N protein is a crucial structural component and collaborates with RdRp to assemble into ribonucleoprotein (RNP), a complex that encapsulates viral RNA and functions as a centre to facilitate viral replication and transcription (Feng et al. 2020; Guo et al. 2017; Richmond et al. 1998). Moreover, N proteins form highly dynamic cytoplasmic inclusion bodies (IBs) that are transported along actin filaments (Feng et al. 2013). Our previous study demonstrated that N protein interacts with RNA and undergoes PS to form intracellular gel‐like condensates, which promote viral proliferation within the plant host (Zan et al. 2025). However, the host factors driving the formation of these BMCs remain unknown, and the disruption of this process could lead to novel antiviral protocols.

In this study, based on our previously published co‐immunoprecipitation mass spectrometry (Co‐IP MS) data (Wang, Luo, et al. 2025), a group of candidate host proteins was selected for luciferase complementation assay (LCA) and bimolecular fluorescence complementation (BiFC) to validate the interaction between N protein and phosphoglycerate kinase (NbPGK) in *Nicotiana benthamiana* (*N*. *benthamiana*). NbPGK, a key enzyme in the glycolytic pathway, catalyses the conversion of 1,3‐bisphosphoglycerate to 3‐phosphoglycerate with concomitant ATP production. We provide evidence that TSWV directly recruits host NbPGK into the condensates of N protein and RNA via PS, thereby remodelling the host glycolytic pathway to provide a foundation for robust viral proliferation. The generated ATP supplies energy for viral replication and non‐enzymatically regulates the interaction network of biomacromolecules within the droplets to promote their formation. Notably, we designed and synthesised a small‐molecule PS regulator, **F10**, which targets the N protein. **F10** binds to critical residues in the TSWV N protein, including Arg94 (R94), Lys192 (K192) and Gly228 (G228), and liberates the hijacked host factor NbPGK. Furthermore, compound **F10** exhibited potent antiviral activity, outperforming the commercial antiviral agent, Ningnanmycin in its ability to inactivate TSWV in a local lesion half‐leaf assay. This study elucidates the molecular machinery by which a plant virus manipulates the host metabolic system to achieve efficient proliferation and identifies a potential antiviral agent for plant protection.

## 2 Results

### 2.1 TSWV N Interacts With the Host Protein Phosphoglycerate Kinase

**FIGURE 1:**
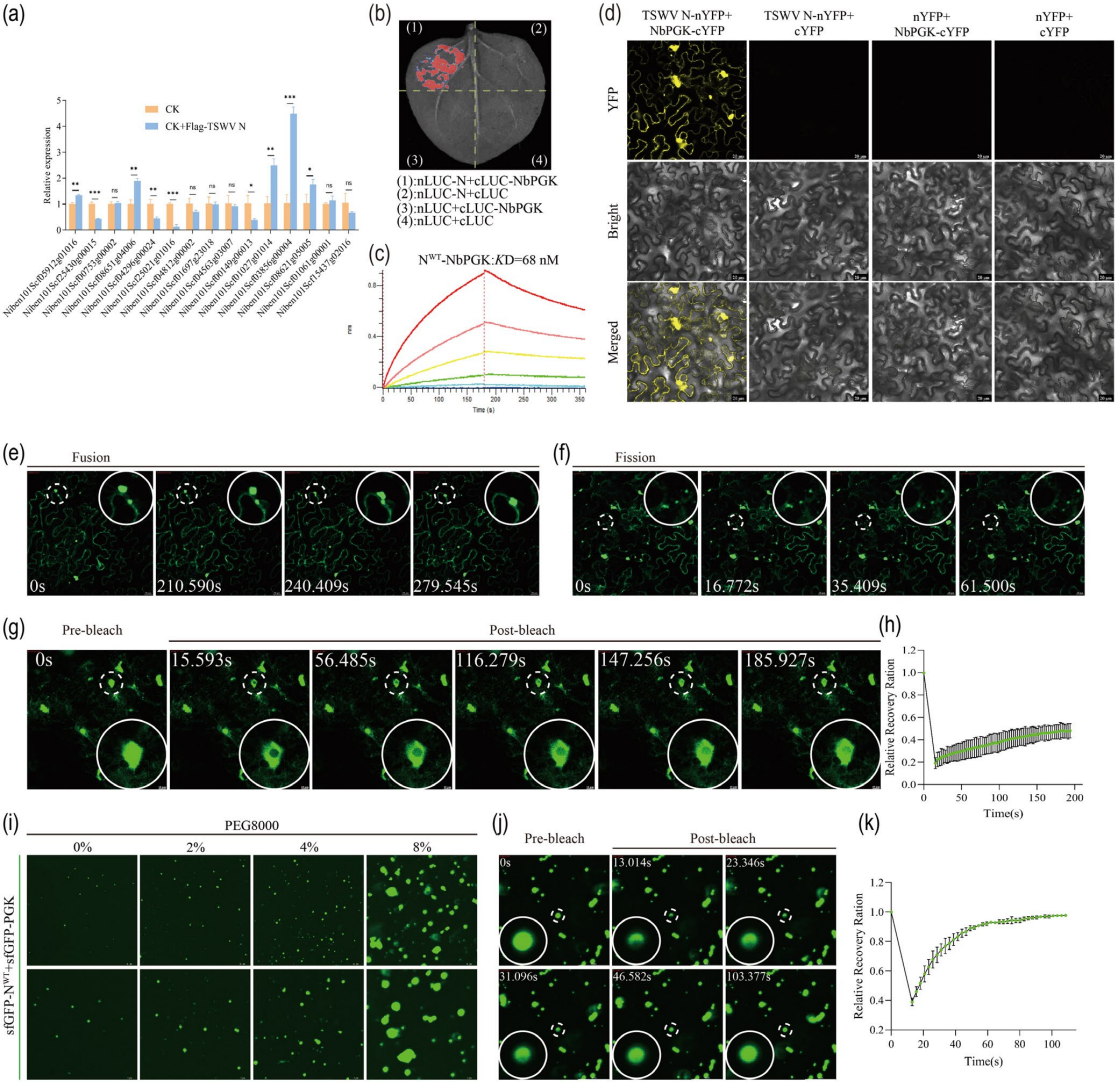
The interaction of TSWV N^WT^ with NbPGK. (a) The upregulation and downregulation of some genes were verified by qRT‐PCR. The experiment was carried out with three mixed samples, and the gene content was calculated by 2^−ΔΔCt^. Data are means SD for three biological replicates. Student's *t‐*test: *p < 0.05, **p < 0.01, ***p < 0.001, *n.s*. indicates *p* ≥ 0.05. (b) Luciferase complementation assays (LCA) analysis the interaction of TSWV N with NbPGK. (c) The binding ability of N^WT^ with PGK was measured by biolayer interferometry (BLI). (d) Bimolecular fluorescence complementation (BiFC) analysis the interaction of TSWV N with NbPGK. Bars, 20 μm. (e) The fusion of IBs was observed by confocal microscopy at 48‐h post‐infiltration (hpi). Bars, 20 μm. (f) The fission of IBs was observed by confocal microscopy at 48 hpi. Bars, 20 μm. (g) Fluorescence recovery after photobleaching (FRAP) analysis of condensates between TSWV N and PGK in *N. benthamiana* leaf cells reveals recovery kinetics. Bars, 10 μm. (h) Quantification of condensates formed by the interaction between TSWV N and PGK in FRAP assays. (*n* = 3 puncta). (i) The interaction between N^WT^ and NbPGK was observed in vitro. Purified sfGFP‐N^WT^ and sfGFP‐NbPGK (10 μL each) were mixed and incubated in buffer containing different concentrations of PEG8000. Bars, 10 μm. (j) FRAP analysis of the droplets formed by sfGFP‐N^WT^ and sfGFP‐NbPGK in vitro. Bars, 10 μm. (k) Plot showing the recovery curves of the FRAP assay shown in (j). (*n* = 3 puncta).

Based on previously published Co‐IP MS data from our group (Wang, Luo, et al. 2025), a subset of candidate host proteins was selected for preliminary analysis. Quantitative reverse transcription polymerase chain reaction (qRT‐PCR) of *N. benthamiana* leaves transiently expressing Flag‐TSWV N revealed that NbPGK was significantly upregulated (Figure 1a and Figure S1). Given previous reports that NbPGK can be hijacked by *Tomato bushy stunt virus* (TBSV) protein to promote viral replication (Prasanth et al. 2017), NbPGK was selected as a candidate interactor for further validation using LCA and BiFC assays. The LCA results (Figure 1b) demonstrated that TSWV N^WT^ interacted with NbPGK, as evidenced by the strong luminescence signals observed in the nLUC‐N + cLUC‐NbPGK combinations. In contrast, no detectable luminescence was observed in the negative controls. BiFC analysis (Figure 1d) demonstrated that TSWV N^WT^ interacted with NbPGK in planta and that the formation of discrete IBs accompanied this interaction. In contrast, no fluorescent signal was detected in the negative control group. These observations further supported the existence of a specific in vivo interaction between TSWV N^WT^ and NbPGK. At 48 h post‐infiltration (hpi), we observed that the interaction induced IBs underwent fusion to form larger granules (Figure 1e and Movie S1), whereas some inclusions also underwent slow fission into smaller granules (Figure 1f and Movie S2). Fluorescence recovery after photobleaching (FRAP) analysis revealed that the fluorescence intensity of the inclusions was recoverable at 48 hpi (Figure 1g,h and Movie S3), suggesting that these IBs possessed gel‐like properties. We performed BiFC assays using coat proteins (CPs) from three representative plant viruses—pepper mild mottle virus (PMMoV, *Tobamovirus*), cucumber mosaic virus (CMV, *Cucumovirus*) and potato virus Y (PVY, *Potyvirus*)—as controls to examine whether NbPGK‐triggered IB formation was specific to its interaction with TSWV N. All three CPs interacted with NbPGK, as indicated by detectable YFP fluorescence signals; however, none of these interactions led to the formation of visible IBs (Figure S2). In contrast, co‐expression of NbPGK with TSWV N^WT^ protein resulted in distinct IBs. These results demonstrated that IB formation is a unique feature of the NbPGK‐N^WT^ interaction, rather than a general property of viral coat protein‐NbPGK associations.

Recombinant sfGFP‐N^WT^ and sfGFP‐NbPGK were purified from *Escherichia coli* lysates (Figure S3), and their ability to form condensates upon in vitro interaction was evaluated (Figure 1i and Figure S4). When sfGFP‐N^WT^ and sfGFP‐NbPGK were mixed at equimolar concentrations (10 μM each), confocal microscopy revealed the formation of small condensates in the absence of PEG8000. The average size of the condensates increased with the addition of 2% PEG8000, and further increasing the PEG8000 concentration (4% and 8%) resulted in progressively larger condensates. FRAP assays on larger droplets showed rapid fluorescence recovery, indicating dynamic molecular exchange within the condensates (Figure 1j,k and Movie S4). These results demonstrated that sfGFP‐N^WT^ can form condensates with NbPGK upon in vitro interaction. This observation is consistent with the BiFC assays performed in planta, in which the N^WT^ protein forms condensates upon interaction with NbPGK. The binding affinity between the N^WT^ and NbPGK was further examined in vitro (Figure 1c), providing additional evidence for the interaction between N and NbPGK.

### 2.2 *NbPGK* Silencing Weakens TSWV Infection

**FIGURE 2:**
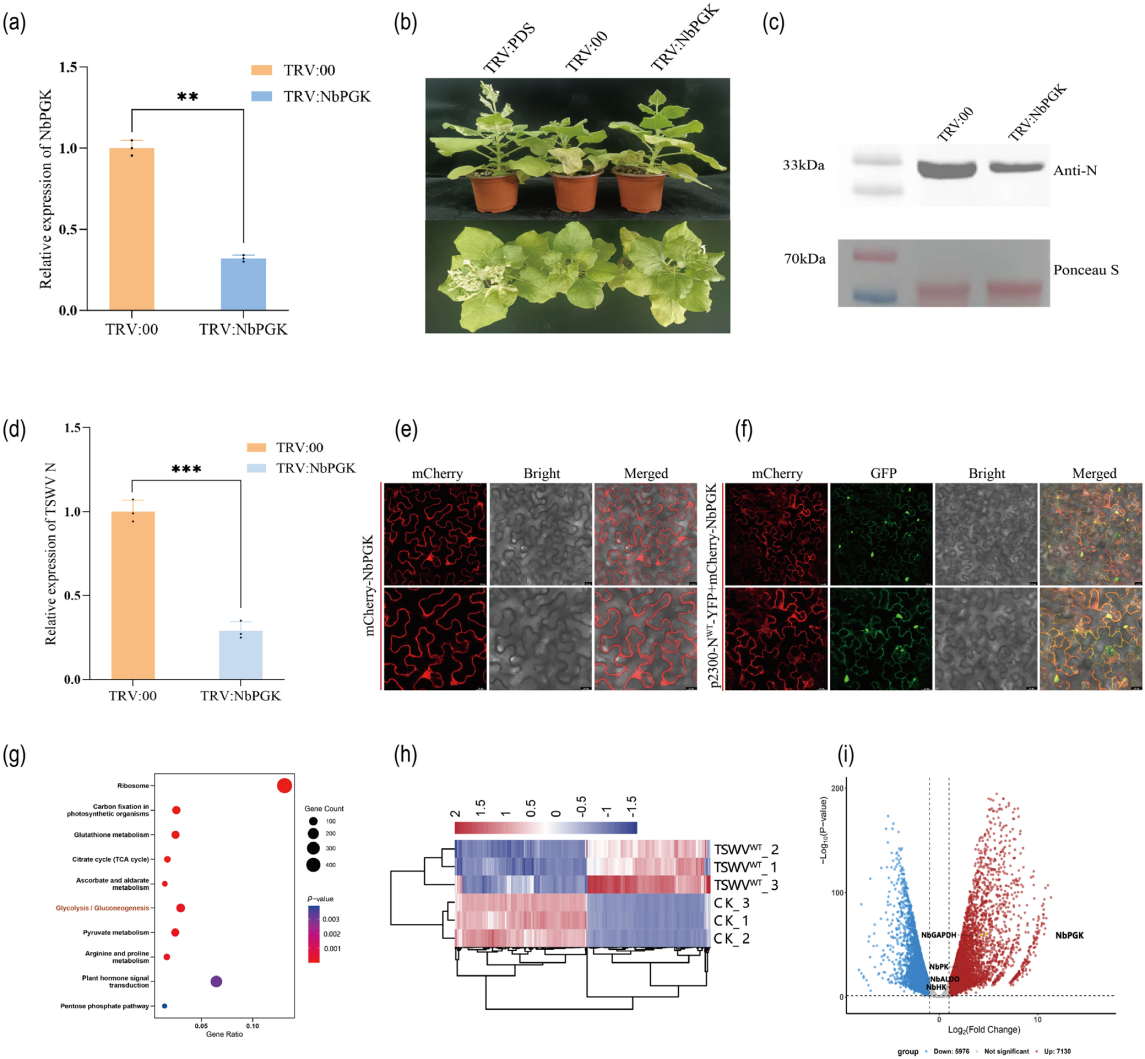
Silencing of *NbPGK* significantly attenuated systemic invasion of TSWV. (a) Detection of NbPGK expression in TRV:NbPGK‐injected *N. benthamiana* using TRV:00 as a negative control at 10 dpi determined by qRT‐PCR. Data are means SD for three biological replicates. Student's *t‐*test: *p < 0.05, **p < 0.01, ***p < 0.001, *n.s*. indicates *p* ≥ 0.05. (b) Observation of the differences in symptoms of TRV:00 and TRV:NbPGK of *N. benthamiana* infected at 21 dpi. (c, d) Western blot and qRT‐PCR showed the accumulation of TSWV N in TRV:NbPGK plants compared to TRV:00. Data are means SD for three biological replicates. Student's *t‐*test: *p < 0.05, **p < 0.01, ***p < 0.001, *n.s*. indicates *p* ≥ 0.05. (e) The transient overexpression vector mCherry‐NbPGK showed no condensate formation in *N. benthamiana* cells at 48 hpi. Bars, 20 μm. (f) Confocal microscopy observation of co‐expression of N^WT^‐YFP and mCherry‐NbPGK at 48 hpi. Bars, 20 μm. (g) KEGG pathway enrichment analysis revealed significant enrichment of the glycolysis pathway in CK and N^WT^ samples. (h) Heatmap showing differentially expressed genes in two biological replicates of CK and WT. (i) Volcano plot highlights significantly altered glycolysis‐related genes between CK and WT.

A tobacco rattle virus (TRV)‐mediated gene silencing system was used to explore the impact of NbPGK on viral infection (Purkayastha and Dasgupta 2009). As shown in Figure 2a, compared with *N. benthamiana* inoculated with TRV:00, the expression level of the NbPGK‐encoding gene was significantly reduced to approximately 30% in plants inoculated with TRV:NbPGK, and no noticeable phenotypic changes were observed at 10 days post‐inoculation (dpi). The *N. benthamiana* plants were subsequently inoculated with TSWV N^WT^ (L_(+)opt_ + SR_(+)eGFP_ + VSRs + M_(−)opt_) and maintained in a greenhouse for 14 days. As shown in Figure 2b, TRV:NbPGK inoculated plants exhibited milder disease symptoms than the TRV:00 control plants, with no noticeable leaf curling in the newly emerged leaves, whereas the control group displayed more pronounced leaf wilting. Further Western blot and qRT‐PCR analyses revealed a marked reduction in the accumulation of TSWV N protein and significant downregulation at the transcriptional level (by approximately 60%–70%) in TRV:NbPGK‐treated plants (Figure 2c,d). Collectively, these results suggested that silencing *NbPGK* in *N. benthamiana* effectively suppressed TSWV genome replication and inhibited systemic viral infection.

### 2.3 NbPGK‐N Interaction Remodels the Host Glycolytic Pathway

A transient overexpression construct of NbPGK fused with an mCherry tag was generated and expressed in *N. benthamiana* leaves to further investigate the regulatory role of NbPGK in the behaviour of N protein. The results showed that mCherry‐NbPGK was diffusely distributed in the cytoplasm without forming visible inclusion bodies, indicating that NbPGK alone cannot drive condensate formation (Figure 2e). However, when co‐expressed with N^WT^‐YFP (Figure 2f), the number and size of N protein granules increased significantly, suggesting that NbPGK promotes condensate formation by interacting with N protein. Notably, partial co‐localisation of mCherry‐PGK with N^WT^‐YFP was observed within some particles, indicating that NbPGK may be recruited to N protein particles. These findings indicate that the TSWV N protein facilitates condensate formation by recruiting the host glycolytic enzyme NbPGK, thereby potentially promoting viral replication and systemic infection in *N. benthamiana*.

We performed transcriptome analysis of *N. benthamiana* leaves transiently expressing Flag‐TSWV N^WT^ and an empty vector control (CK) to study the underlying mechanism of N protein‐induced condensate formation. Differentially expressed genes (DEGs) were identified using a cutoff of |log_2_FoldChange| > 1 and *p*‐value of < 0.05. KEGG and GO enrichment analyses revealed that the glycolysis pathway was significantly enriched in the CK‐N^WT^ group (Figure 2g and Figure S5), suggesting that the N protein may activate and remodel the host glycolytic pathway through its interaction with NbPGK.

A heatmap illustrating the transcriptional changes between the CK and N^WT^ samples (Figure 2h). Sample clustering indicated high reproducibility within the groups, and the DEGs displayed distinct expression patterns. A subset of genes was significantly upregulated, whereas others were downregulated, implying their involvement in viral infection responses or regulatory pathways. Volcano plot analysis of the glycolysis‐related DEGs showed that hexokinase (NbHK), aldolase (NbALDO), NbPGK and pyruvate kinase (NbPK) were significantly upregulated in the N^WT^ group, with NbPGK exhibiting the most pronounced change. In contrast, glyceraldehyde‐3‐phosphate dehydrogenase (NbGAPDH) expression was significantly downregulated (Figure 2i). Together, these results indicated that the N protein, through its interaction with NbPGK, may reprogramme host glycolysis to create a localised metabolic advantage, thereby facilitating viral replication and infection.

**FIGURE 3:**
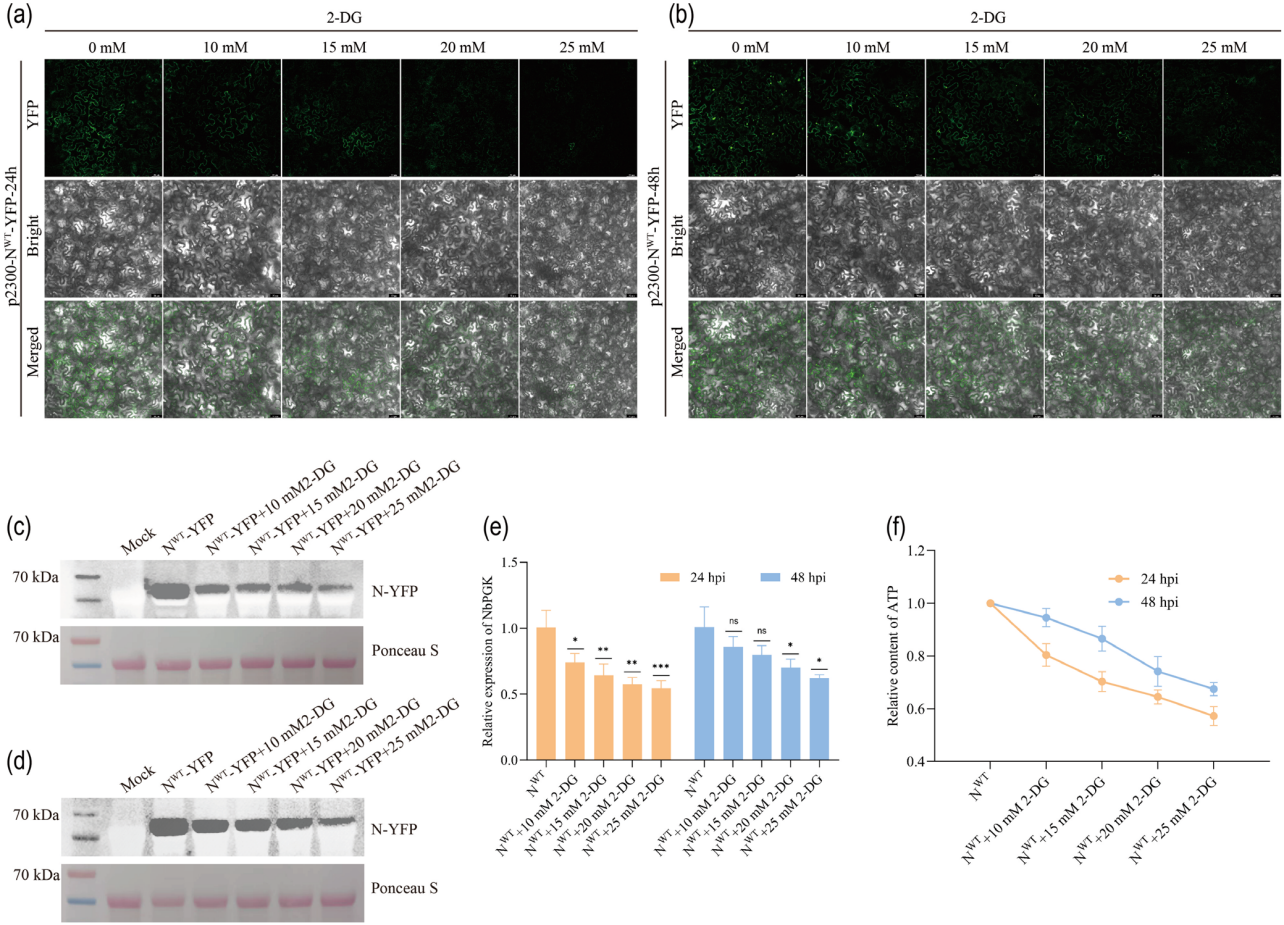
2‐deoxy‐D‐glucose (2‐DG) significantly inhibited the formation of condensates. (a, b) N^WT^‐YFP was expressed in *N. benthamiana*, and after treatment with 2‐DG, changes in intracellular condensates were observed by confocal microscopy at 24 and 48 hpi. Bars, 50 μm. (c, d) Western blot was used to detect the accumulation level of N‐YFP in the infiltrated leaves. Staining of RuBisCO with Ponceau S was used as a sample loading control. (e) Relative expression levels of NbPGK at 24 and 48 hpi after treatment with 2‐DG, as determined by qRT‐PCR. Data are means SD for three biological replicates. Student's *t‐*test: *p < 0.05, **p < 0.01, ***p < 0.001, *n.s*. indicates *p* ≥ 0.05. (f) Relative expression levels of ATP at 24 and 48 hpi after treatment with 2‐DG.

*N. benthamiana* leaves expressing N^WT^‐YFP were treated with the glycolytic inhibitor 2‐deoxy‐D‐glucose (2‐DG) following *Agrobacterium*‐mediated infiltration to investigate the role of glycolysis in the formation of N protein‐associated granules (Lin et al. 2020; Liu et al. 2018). Condensate formation in N^WT^‐YFP expressing cells was markedly inhibited by 2‐DG treatment in a dose‐dependent manner, as observed using confocal microscopy at 24 and 48 hpi (Figure 3a,b). Building on these results, the accumulation of N^WT^‐YFP after 2‐DG treatment was further quantified using Western blot, which revealed a pronounced reduction in N‐YFP levels at 24 and 48 hpi (Figure 3c,d). These findings suggest that condensate formation is essential for stabilising the N protein (Perdikari et al. 2020; Wei et al. 2022; Zhang et al. 2023). Glycolytic inhibition disrupts condensate assembly, consequently diminishing N protein stability, and potentially facilitating accelerated degradation within the host plant (Zan et al. 2025). The qRT‐PCR analysis demonstrated a dose dependent decline in the relative expression levels of *NbPGK* following treatment with increasing concentrations of 2‐DG (Figure 3e). Quantification of the ATP levels revealed a concentration‐dependent decrease upon treatment with escalating doses of 2‐DG (Figure 3f). These findings indicated that 2‐DG effectively suppressed ATP synthesis by inhibiting glycolytic flux, potentially mediated by the downregulation of *NbPGK* expression.

### 2.4 Glycolysis‐Derived ATP Promotes N Protein PS

**FIGURE 4:**
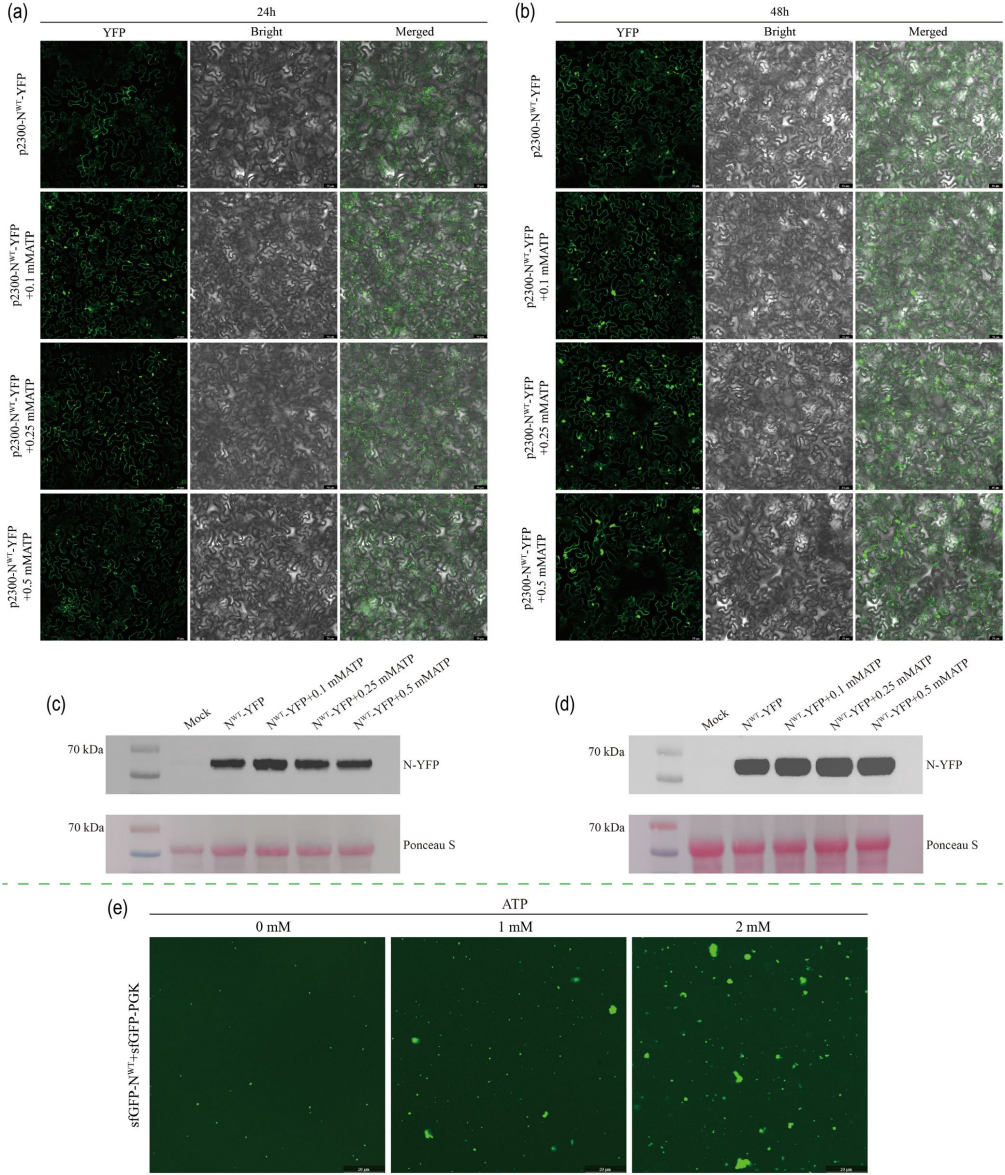
Exogenous ATP promotes N^WT^‐YFP condensate formation in a concentration dependent manner. (a, b) *N. benthamiana* leaves expressing N^WT^‐YFP were co‐treated with 0.1, 0.25 or 0.5 mM ATP, and condensate formation was assessed by confocal microscopy at 24 and 48 hpi. Bars, 50 μm. (c, d) Western blot was used to detect the accumulation level of N‐YFP in the infiltrated leaves. Staining of RuBisCO with Ponceau S was used as a sample loading control. (e) Under in vitro conditions, the formation of condensates resulting from the interaction between sfGFP‐N^WT^ and sfGFP‐PGK was observed following ATP supplementation.

Glycolysis is widely recognised as the principal metabolic pathway responsible for cellular energy production in plants, yielding two ATP molecules per metabolised glucose molecule (Chandel 2021; Prasanth et al. 2017). Exogenous ATP at various concentrations (0.1, 0.25 and 0.5 mM) was co‐applied with N‐YFP via agroinfiltration into *N. benthamiana*, and condensate formation was monitored at 24 and 48 hpi to assess the influence of glycolytic activity on condensate formation. As shown in Figure 4a, at 24 hpi, treatment with 0.1 mM ATP markedly promoted the formation of N protein granules, as evidenced by a significant increase in both the number and size of granules. In contrast, the 0.25 mM and 0.5 mM ATP treatments resulted in only slight increases in condensate formation compared to that with the control. At 48 hpi (Figure 4b), condensate formation in the 0.1 mM ATP group was comparable to that in the N^WT^‐YFP group, whereas the 0.25 mM ATP treatment resulted in a pronounced increase in condensate number, and the 0.5 mM group also exhibited a moderate increase. These results suggested that ATP regulates N protein condensate formation in a concentration and time‐dependent manner. We performed Western blot analysis of samples treated with different ATP concentrations to further assess the effects of ATP on N‐YFP accumulation (Figure 4c,d). At 24 hpi, the 0.1 mM ATP treatment resulted in elevated levels of N‐YFP accumulation, while the 0.25 mM and 0.5 mM groups showed band intensities similar to that of N^WT^‐YFP. At 48 hpi, N‐YFP accumulation was significantly increased in the 0.25 mM ATP group, and moderately elevated in both the 0.1 and 0.5 mM groups relative to the control. Collectively, these results indicated that appropriate levels of ATP can promote the formation of stable N protein condensates, potentially by enhancing protein stability or reducing degradation, and that this effect is concentration‐ and time‐dependent. We quantitatively measured their binding affinity using biolayer interferometry (BLI) and microscale thermophoresis (MST) to confirm whether ATP directly interacts with N proteins to mediate these effects. Both assays revealed a strong and specific interaction, with equilibrium affinity constants (*K*D) of 0.48 μM (BLI) and 1.49 μM (MST) (Figure S6). These results demonstrated that ATP binds tightly to the N protein, supporting the notion that ATP may act as a direct regulatory factor in N‐mediated condensate formation and RNP assembly.

We performed coarse‐grained molecular dynamics (CGMD) simulations to test whether ATP exerted a non‐enzymatic effect on the condensation of N proteins. In the model, ATP was represented by three representative components, that is, adenine, ribose and phosphate, to capture both electrostatic and hydrophobic interactions with the N protein (Figure S7a). Six ATP‐ N protein ratios (A:N = 0, 1, 5, 10, 50 and 500) were used to evaluate the effect of the ATP concentration on condensate formation. The simulation results indicated that ATP promotes N protein condensate formation at low concentrations but inhibits PS formation at high concentrations (Figure S7c,d). Specifically, within the low concentration range (A:N = 1–50), the Δ*P* value gradually increased with rising ATP concentration, indicating enhanced intermolecular interactions within condensates; however, at A:N = 500, Δ*P* decreased markedly, even below that of the ATP‐free system (Figure S7e). Cluster number analysis showed a gradual reduction in cluster quantity as the concentration increased from 0 to 50, suggesting that ATP mediates N protein intermolecular interactions via bridging. Under high concentration conditions (A:N = 500), the cluster number remained unchanged and was consistently higher than that of the system without ATP (Figure S7f). Furthermore, mean square displacement (MSD) analysis revealed that the molecular mobility decreased progressively with increasing ATP concentration, facilitating condensate formation (Figure S7g).

We examined the effects of ATP on condensate formation in vitro. Following incubation with 1 or 2 mM ATP, sfGFP‐N^WT^ and sfGFP‐PGK showed an increased propensity to form condensates in an ATP concentration‐dependent manner. Confocal microscopy revealed that increasing ATP concentrations led to a gradual increase in both the number and size of condensates (Figure 4e). These findings further supported the idea that ATP promotes PS of viral and host proteins, underscoring the pivotal role of glycolysis‐derived ATP in modulating BMCs dynamics. This indicates that active glycolysis is essential for condensate dynamics and further supports the role of glycolytic pathway remodelling in promoting TSWV replication and infection.

### 2.5 Small‐Molecule **F10** Significantly Reduces N Protein Condensates and Proper Replication of RNPs by Targeting the N Protein

**FIGURE 5:**
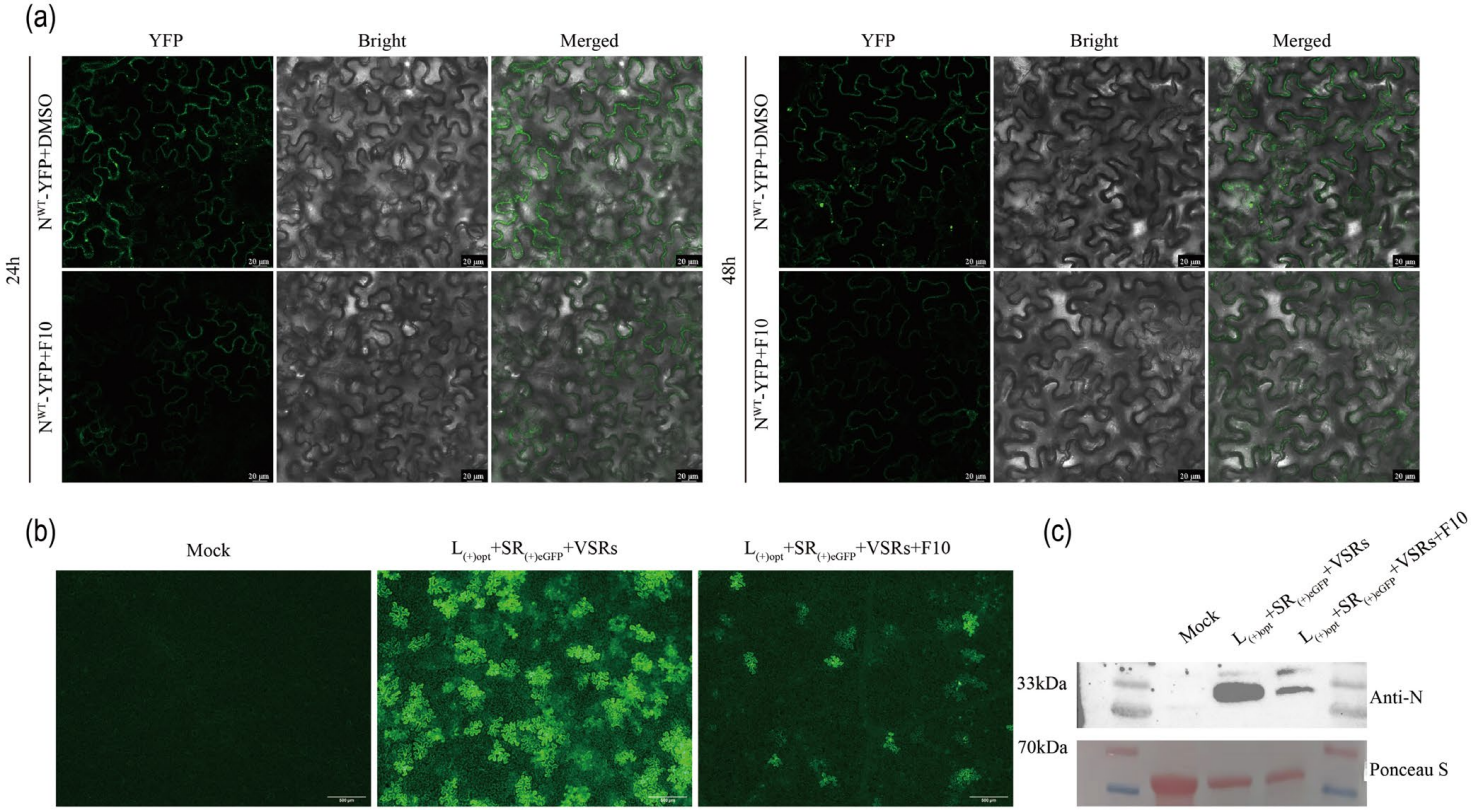
Compound **F10** inhibits significantly reduces N protein condensates and proper replication of RNPs. (a) Representative fluorescence images showing compound **F10** could significantly reduce the formation of IBs in *N. benthamiana leaves* at 24 and 48 hpi. DMSO was used as a control. Bars, 20 μm. (b) The amounts of fluorescence in the infiltrated leaves were observed at 5 dpi. Bars, 500 μm. (c) Western blot was used to detect the accumulation level of N protein in the infiltrated leaves. Staining of RuBisCO with Ponceau S was used as a sample loading control.

The TSWV N protein assembles into dynamic cytoplasmic IBs, which are essential for viral genome replication and propagation (Feng et al. 2013). Based on the mechanism by which TSWV hijacks host glycolysis through its N protein, we strategically designed small molecules to intervene in this process, potentially restoring the normal function of the host NbPGK. Accordingly, we synthesised a series of potential PS modulators (Scheme S1) and evaluated their effects on the formation of N protein condensates and their in vivo behaviours. At 18 hpi, either the synthetic compound working solution or DMSO vehicle control was introduced into distinct regions of the same *N. benthamiana* leaf. IB formation was assessed at 24 and 48 hpi. Compared with the DMSO control, treatment with **F10** resulted in a pronounced reduction in the number of small IBs within the infiltrated leaf regions (Figure 5a). By 48 hpi, the IB count in the **F10** treated areas had declined by more than 50%. These findings indicated that **F10** impairs IB formation of N protein.

The N protein induces the viral genomic RNA to form RNP, acting as a central scaffold that orchestrates RNP assembly and plays an essential role in viral genome replication and transcription (Li et al. 2015). The RNP serves as the central molecular machinery that facilitates viral genome transcription and replication throughout the infection cycle (Hong et al. 2021; Wu et al. 2024). A functional TSWV minigenome replication system consisting of L_(+)opt_, SR_(+)eGFP_ and VSRs was used to evaluate the effects of **F10** on RNP‐mediated viral replication in *N. benthamiana* (Feng et al. 2020). At 5 dpi, fluorescence microscopy revealed a substantial attenuation of green fluorescence in leaf tissues treated with **F10** (Figure 5b). Western blotting analysis (Figure 5c) consistently showed a marked decrease in N protein accumulation in **F10**‐treated samples relative to untreated controls. Collectively, these results provide compelling evidence that compound **F10** potently inhibits TSWV RNP replication in planta.

We assessed the anti‐TSWV efficacy of these compounds using a local lesion half‐leaf assay on *Nicotiana glutinosa* ( *N. glutinosa* ) plants to investigate whether inhibiting the formation of N protein condensates could concurrently confer antiviral activity. As presented in Tables S1 and S2, the results showed that compounds **F10**, **F19** and **F28** exhibited significantly enhanced inactivation activities, with EC_50_ values of 81.83 μg/mL, 127.79 μg/mL and 109.38 μg/mL, respectively, compared to that of Ningnanmycin (136.29 μg/mL). Moreover, compounds **F10**, **F13** and **F15** showed superior protective activities, with EC_50_ values of 143.77 μg/mL, 152.72 μg/mL and 174.58 μg/mL, respectively, which were also better than that of Ningnanmycin (198.31 μg/mL). The bio‐assay results indicated that compound **F10** has potent inactivation activity against TSWV. To further evaluate the biosafety and practical applicability of compound **F10** in host plants, we examined the phytotoxicity and antiviral performance in tomato ( *Solanum lycopersicum* , M82). Tomato plants were treated with **F10**, whereas water‐treated plants served as controls. Phenotypic observations were conducted 5 days after treatment, and no visible morphological or growth abnormalities were detected in the **F10**‐treated group compared with that in the controls (Figure S8), indicating that **F10** exhibited no detectable phytotoxic effects under the tested conditions. Furthermore, in TSWV‐infected tomato plants, no apparent leaf wilting or necrotic symptoms were observed in the **F10**‐treated group compared with that in the wild‐type control (Figure S9a), whereas the relative transcript level of the N gene was significantly reduced (Figure S9b). These findings demonstrated that **F10** is non‐phytotoxic and remains effective in suppressing TSWV infection not only in model plants but also in natural host species.

The total protein was extracted from *N. benthamiana* leaves infected with TSWV and subjected to a drug affinity‐responsive target stability (DARTS) assay to identify candidate proteins that may directly bind to **F10** and identify the molecular target of **F10**. Total protein extracts from the infected leaves were incubated with **F10** and subsequently subjected to limited proteolysis using pronase. A markedly intensified protein band between 25–33 kDa was observed in the **F10**‐treated samples, indicating that the interaction with **F10** may enhance resistance to proteolytic degradation (Figure S10). Mass spectrometry analysis of the excised protein bands further verified the identity of the N protein (Table S3). These results provide direct evidence that the N protein serves as a molecular receptor for compound **F10**.

### 2.6 The Absence of Key Binding Sites for **F10** to N Protein Significantly Impairs Viral Proliferation In Vivo

**FIGURE 6:**
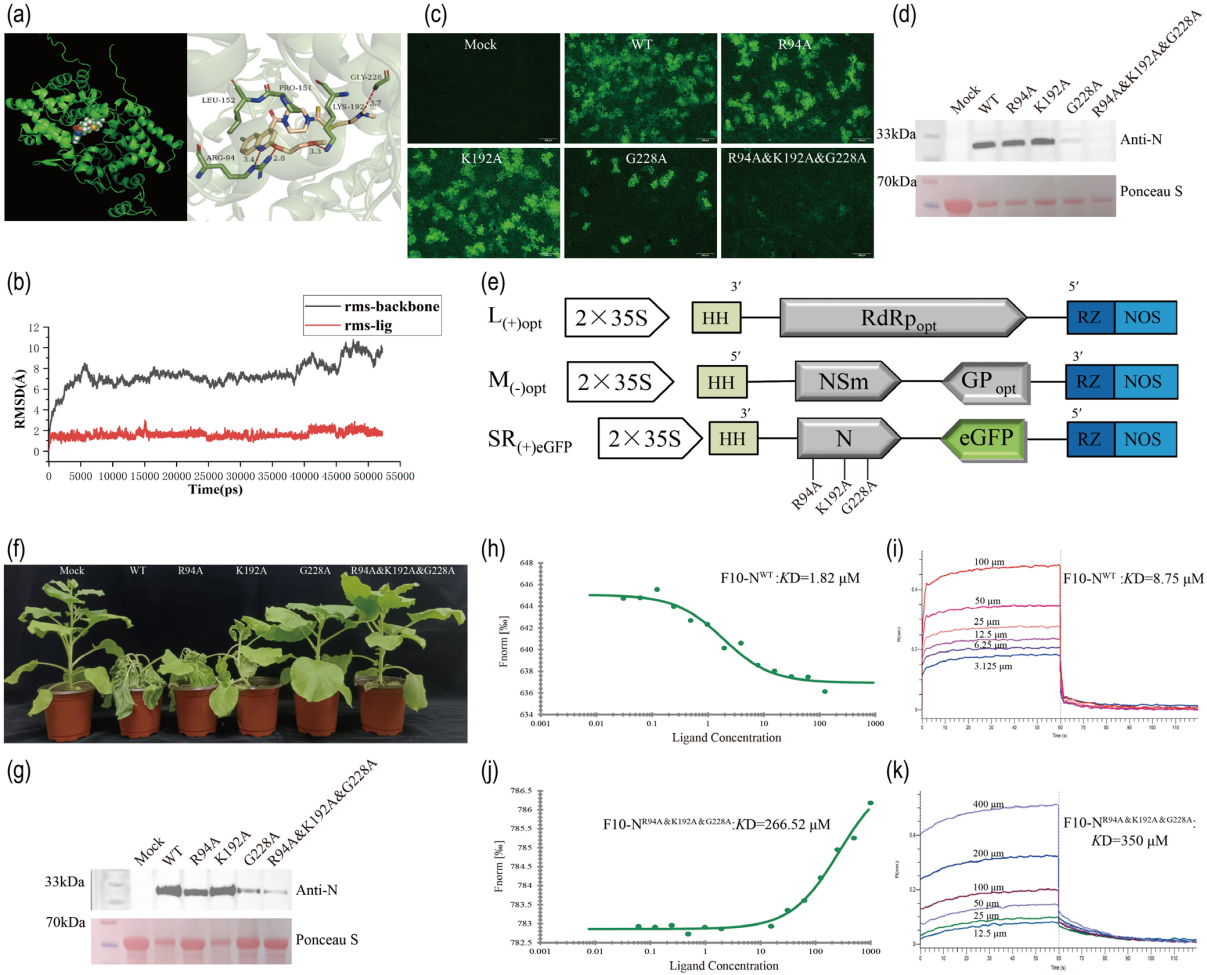
Identification and validation of key amino acid residues in TSWV N protein involved in **F10** action. (a) Molecular docking of **F10** with TSWV N. (b) Molecular dynamics simulation studies of compound **F10** with TSWV N. (c) The amounts of fluorescence in the infiltrated leaves were observed at 5 dpi. Bars, 500 μm. (d) Western blot was used to detect the accumulation level of N protein in the infiltrated leaves. Staining of RuBisCO with Ponceau S was used as a sample loading control. (e) Genomic structure of TSWV infectious cloning. (f) Phenotypes of TSWV^WT^ and TSWV^Mut^ infected plants on the 21 dpi with at least 10 plants in each experiment. (g) The accumulation level of N protein in the systemic leaves was detected by Western blot. Staining of RuBisCO with Ponceau S was used as a sample loading control. (h, i) The binding ability of compound **F10** with N was measured by microscale thermophoresis (MST) and BLI. (j, k) The binding ability of compound **F10** with N^R94A&K192A&G228A^ was measured by MST and BLI.

To gain mechanistic insights into the anti‐TSWV activity of **F10**, a molecular docking analysis was conducted using AutoDock 4.2, with the TSWV N protein (PDB ID: 5IP1) as the molecular target. Docking analysis revealed that **F10** formed stable hydrogen bonds with the residues Arg94, Lys192 and Gly228 of the N protein, potentially contributing to enhanced binding affinity and target specificity (Figure 6a). Molecular dynamics (MD) simulations were performed to evaluate the binding stability between **F10** and the N protein. A total of 100 snapshots from the last 1 ns of the simulation trajectory were extracted for the analysis. The root mean square deviation (RMSD) profiles of both the N protein backbone and **F10** ligand remained relatively stable throughout the simulation, suggesting that the protein‐ligand complex maintained its overall structural integrity (Figure 6b). The calculated binding free energy (Δ*G*) was −38.07 kcal/mol, suggesting a stable interaction and strong binding affinity between **F10** and the N protein.

To assess the function of key amino acid residues implicated in the interaction between compound **F10** and the TSWV N protein during RNP replication, a series of mutant plasmids, SR_(+)eGFP_^Mut^, including SR_(+)eGFP_^R94A^, SR_(+)eGFP_^K192A^, SR_(+)eGFP_^G228A^ and SR_(+)eGFP_^R94A&K192A&G228A^, were constructed and introduced into *N. benthamiana* leaves via the RNP_eGFP_ system (Figure 6c). Compared to RNP_eGFP_^WT^, the K192A single mutation had no appreciable effect on fluorescence intensity, whereas the R94A mutation caused a slight reduction in fluorescence signals. The G228A single mutation led to a moderate decrease in fluorescence intensity. In contrast, the triple‐site mutants (R94A&K192A&G228A) exhibited a pronounced reduction in fluorescence signals. Simultaneously, the accumulation of N proteins was evaluated (Figure 6d). Relative to RNP_eGFP_^WT^, the triple‐site mutant displayed undetectable levels of N protein at 5 dpi. The G228A single mutant showed low but detectable N protein accumulation, whereas the R94A mutation resulted in a slight decrease. In contrast, the K192A mutation had a minimal effect on N protein levels.

To further examine the effects of binding site mutations on viral pathogenicity in *N. benthamiana*, the M_(−)opt_ segment was incorporated to facilitate systemic infection with the recombinant virus (Figure 6e). At 21 dpi, plants infected with TSWV N^WT^ exhibited pronounced disease symptoms, including complete wilting and mortality, resulting in 100% disease incidence. In contrast, plants inoculated with the mutant strains exhibited delayed symptom onset and significantly attenuated disease severity (Figure 6f). Among them, plants infected with TSWV N^K192A^ showed a disease incidence of approximately 90%, with symptom severity similar to that of TSWV N^WT^. Plants infected with TSWV N^R94A^ exhibited a disease incidence of approximately 60%, with a noticeable reduction in systemic leaf symptoms. In plants infected with TSWV N^G228A^, the disease incidence decreased to approximately 30%; plants inoculated with the triple‐site mutant TSWV N^R94A&K192A&G228A^ exhibited the mildest symptoms, with the disease incidence reduced to approximately 10%, and no evident symptoms were observed in systemic leaves. Subsequent Western blot analysis revealed that, compared to TSWV N^WT^, the expression level of the N protein in the systemic leaves of TSWV N^R94A&K192A&G228A^ was significantly reduced. Additionally, TSWV N^R94A^, TSWV N^K192A^ and TSWV N^G228A^ also exhibited varying degrees of reduced N protein accumulation (Figure 6g). In summary, the integrated analysis of symptom development and immunoblotting data revealed that triple site mutations (R94A, K192A and G228A) in the TSWV N protein substantially impaired the capacity of the virus for systemic spread and led to a pronounced reduction in virulence.

The binding interaction between compound **F10** and the N protein was further quantified using MST and BLI. MST analysis revealed that the *K*D of **F10** binding to N^WT^ was 1.82 μM (Figure 6h), which was markedly stronger than its affinity for the N^R94A&K192A&G228A^ (266 μM) (Figure 6j). Similarly, BLI analysis revealed a significantly reduced binding affinity of **F10** to the triple mutant, with a *K*D of 350 μM, compared to 8.75 μM for N^WT^ (Figure 6i,k). These findings demonstrated that the triple mutation (R94A, K192A and G228A) substantially diminished the binding affinity of **F10**, highlighting the critical role of these residues in mediating the interaction between **F10** and the N protein.

### 2.7 Mutations at the Binding Sites Markedly Inhibit the Formation of Condensates

**FIGURE 7:**
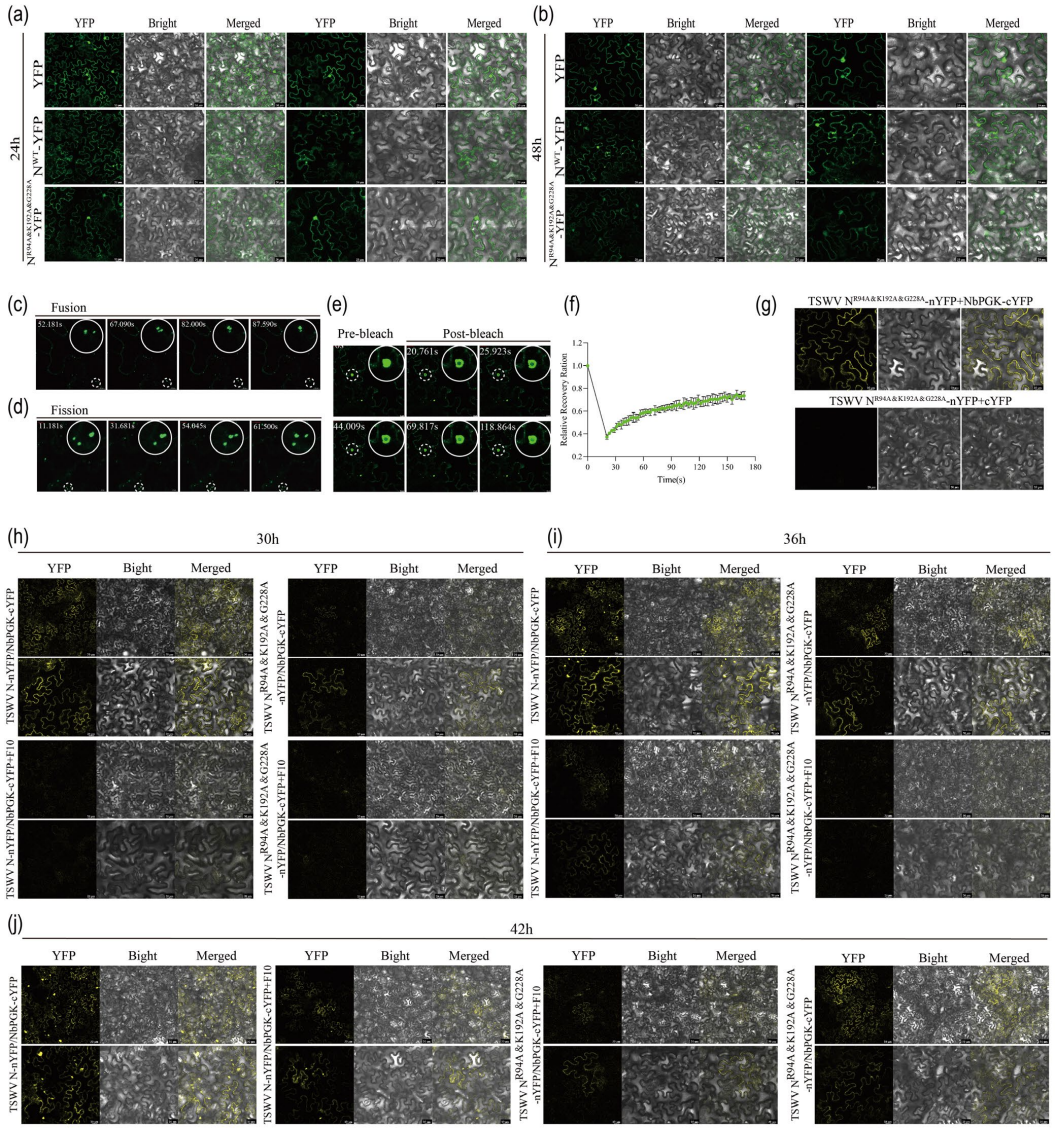
The key binding sites significantly reduce the aggregation of N protein in vivo. (a, b) observed the Condensates formed by N‐YFP and N^R94A&K192A&G228A^‐YFP by confocal microscopy at 24 and 48 hpi, with empty vector YFP used as a control. Bars, 20 μm. (c) Fluorescence time‐lapse confocal images showing the fusion of IBs. The white circles indicated the IBs undergoing fusion at 48 hpi. Bars, 10 μm. (d) Fluorescence time lapse confocal images showing the fission of IBs. The white circles indicated the IBs undergoing fission at 48 hpi. Bars, 10 μm. (e) FRAP of small IBs formed in *N. benthamiana* cells. The images were taken every 2.59 s for 168 s to document fluorescence recovery, each time point was normalised to before photobleaching. Bars, 10 μm. (f) Quantification of N‐YFP condensates in FRAP assays (*n* = 3 puncta). (g) BiFC analysis the interaction of TSWV N^R94A&K192A&G228A^ with NbPGK. Bars, 20 μm. (h–j) At 30, 36 and 42 hpi, the differences in the interaction between TSWV N or TSWV N^R94A&K192A&G228A^ and NbPGK after **F10** treatment were observed. Bars, 50 and 20 μm.

We transiently expressed the constructed mutant plasmids N^R94A&K192A&G228A^‐YFP in *N. benthamiana* leaves, using p2300‐YFP as a control, to elucidate the role of the binding sites in the formation of condensates (Figure 7a,b). At 24 hpi, N^WT^‐YFP formed numerous small, discrete cytoplasmic IBs, whereas no obvious inclusion structures were observed for N^R94A&K192A&G228A^‐YFP. At 48 hpi, N^WT^‐YFP further aggregated into larger granules, whereas the fluorescence signals of the N^R94A&K192A&G228A^‐YFP mutant remained weak. These observations indicated that mutations at the binding sites significantly impair the ability of the N protein to form granules, a phenomenon that closely resembles the effect observed upon treatment with **F10**. At 48 hpi, fusion (Figure 7c and Movie S5), fission (Figure 7d and Movie S6) and FRAP assays (Figure 7e,f and Movie S7) were performed on N^WT^‐YFP expressing cells, further supporting the hypothesis that the N protein may exhibit gel‐like properties. BiFC analysis has previously demonstrated that TSWV N^WT^ interacts with NbPGK in planta, and that this interaction is associated with the formation of discrete granules. The interactions between N^R94A&K192A&G228A^ and NbPGK were assessed to elucidate further the functional relevance of specific amino acid residues in this process. Although the mutant retained its capacity to interact with NbPGK, no condensate formation was detected (Figure 7g), further supporting the critical importance of these key amino acid residues and their essential roles in condensate formation. In addition, it was clearly observed that treatment with compound **F10** also affected the interaction between TSWV N^WT^ or TSWV N^R94A&K192A&G228A^ and NbPGK (Figure 7h,i,j).

## 3 Discussion

In recent years, the aggregation of viral proteins has been increasingly recognised as a critical mechanism underlying viral replication and infection (Guseva et al. 2020; Heinrich et al. 2018). In a recent study, Zan et al. (2025) used a rational design strategy to develop small molecule inhibitors targeting the N protein, and demonstrated that these compounds effectively disrupted condensate formation by the N protein of TSWV, thereby significantly suppressing systemic viral infection and accumulation. These findings underscore the essential role of N protein condensate formation in the TSWV life cycle. However, the extent to which this process is modulated by the host cellular factors remains poorly understood. We systematically identified host plant proteins that interact with the N protein to reveal the host‐dependent regulatory network underlying viral PS dynamics to elucidate further the regulatory mechanisms involved in N protein aggregation. In this study, subsequent analyses identified the glycolytic enzyme NbPGK as a critical host interactor for the TSWV N protein. BiFC and LCA assays validated the interaction between NbPGK and the N^WT^ (Figure 1b,d). Interestingly, the BiFC analysis demonstrated that the interaction between NbPGK and N^WT^ resulted in the formation of discernible IBs. The interaction between NbPGK and N^WT^ was examined in vitro, and the results were consistent with those observed in vivo (Figure 1i).

Virus‐induced gene silencing (VIGS) was employed to investigate the functional role of NbPGK during TSWV infection and was found to regulate viral infection positively. Furthermore, the co‐expression of NbPGK with the N protein markedly enhanced both the number and size of granules, accompanied by partial colocalization, suggesting that the N protein may hijack the host NbPGK to facilitate condensate formation, thereby promoting viral replication and infection. This finding is consistent with those for previous reports (Chaturvedi et al. 2016; Hyodo et al. 2019), Glyceraldehyde‐3‐phosphate dehydrogenase (GAPDH) and receptor for activated C kinase 1 (RACK1) have been identified as critical host cofactors for the replication of RNA viruses, particularly *Cucumber mosaic virus* (CMV) and *Red clover necrotic mosaic virus* (RCNMV), respectively. In addition to its canonical role in glycolysis, GAPDH contributes to the assembly of the viral replication complex by stabilising interactions among CMV replication associated proteins, thereby facilitating viral RNA synthesis. RACK1, a multifunctional scaffold protein, is hijacked by RCNMV to function as a molecular bridge between the viral replication protein p27 and the host calcium dependent protein kinase (CDPK), thereby facilitating viral RNA translation and replication. These findings illustrate that viruses hijack not only metabolic enzymes to fulfil their energy demands, but also repurpose structural and regulatory host proteins as molecular scaffolds to orchestrate their replication cycle precisely.

Transcriptome profiling was performed to elucidate the mechanism underlying N protein condensate formation. The results revealed that N protein reprogrammed the host glycolytic pathway by significantly upregulating key enzymes, including NbPGK, NbHK, NbALDO and NbPK, suggesting that glycolytic remodelling is essential for the interaction between N protein and NbPGK, which may confer a localised metabolic advantage for viral replication. Therefore, the glycolytic inhibitor 2‐DG was utilised to pharmacologically interfere with the host glycolytic pathway (Lin et al. 2020; Liu et al. 2018). The results demonstrated that 2‐DG treatment markedly inhibited condensate formation in a concentration‐dependent manner (Figure 3a,b). We also observed that the relative expression of NbPGK progressively decreased with increasing 2‐DG concentrations (Figure 3e).

As a key glycolytic enzyme that interacts with the N protein, NbPGK is not only responsible for catalysing ATP generation via substrate level phosphorylation (Chuang et al. 2017; Prasanth et al. 2017), but is also involved in the formation of BMCs through its direct physical association with the N protein (Dang et al. 2021; Kang et al. 2018; Toyama et al. 2022; Zalar et al. 2023). We hypothesised that the ATP generated within the replication compartment as a consequence of the interaction between the N protein and NbPGK plays two pivotal roles. First, it functions as an energy currency to support the replication of the viral genome following the assembly of the N protein into the RNP. Previous studies have demonstrated that viral infection promotes efficient replication by enhancing host glucose uptake, upregulating key glycolytic enzymes, and generating ATP locally in the vicinity of viral replication complexes (Fontaine et al. 2015; Prasanth et al. 2017; Qian et al. 2022). Secondly, ATP may facilitate BMC formation through biophysical interactions. CGMD simulations revealed that ATP enhanced intermolecular interactions among N proteins at low concentrations, thereby promoting condensate formation, whereas high concentrations of ATP inhibited PS (Figure S7). These results suggest that ATP regulates N‐protein condensate formation in a concentration‐dependent manner through a non‐enzymatic mechanism. Kota et al. (2024) demonstrated that ATP acts as a multivalent scaffold, bridging extensive intermolecular interactions to drive PS of basic intrinsically disordered proteins. This mechanism partially aligns with the findings of this study. The TSWV N protein possesses intrinsically disordered regions and several basic residues. Additionally, by incorporating host proteins into the condensate matrix, TSWV may mask or sequester host immune factors, potentially aiding immune evasion. The resulting condensates are presumed to stabilise the N protein within host cells, reducing their susceptibility to host‐mediated degradation and thereby establishing a favourable intracellular environment for successful viral infection. Collectively, these findings highlight the critical role of the glycolytic pathway, particularly NbPGK, in facilitating RNP assembly and condensate formation, which are essential for efficient TSWV replication and infection. Exogenous ATP was applied both in vivo and in vitro, resulting in a concentration‐dependent enhancement of N protein condensate formation. Specifically, low concentrations of ATP (0.1–0.5 mM) significantly increased the number and size of N‐YFP granules (Figure 4a,b). Notably, at 48 hpi, the 0.25 mM ATP treatment group exhibited a greater number of granules than the N^WT^‐YFP control, while at 24 hpi, the 0.1 mM ATP group showed the highest condensate density. This observation is consistent with previous studies reporting that ATP promotes condensate formation at low concentrations but inhibits it at higher concentrations (Kota et al. 2024; Liu and Wang 2023; Ren et al. 2022). This effect may be closely related to the dynamic consumption of ATP within plant cells, where 0.25 mM ATP at 48 hpi may represent an optimal concentration. In vitro assays further confirmed that the addition of 1–2 mM ATP enhanced the formation of condensates by sfGFP‐N^WT^ and host protein sfGFP‐NbPGK (Figure 4e). These findings provide further evidence that the glycolytic pathway enzyme NbPGK plays a critical role in meeting the energy demands required for viral replication through ATP generation and the regulation of N protein condensate formation, thereby contributing to the subsequent TSWV infection process.

Considering the essential role of NbPGK‐mediated glycolysis in driving ATP‐dependent condensate formation by the N protein and promoting TSWV replication, further structural optimization was performed based on previously identified small molecules capable of disrupting PS (Zan et al. 2025). A series of candidate molecules was subsequently selected through rational design and screening with the aim of specifically inhibiting this mechanism and ultimately achieving effective suppression of TSWV infection. Chloroquine (CQ), a synthetic 4‐aminoquinoline derivative, was first clinically used in 1944, primarily for malaria treatment (Thomé et al. 2013). In subsequent decades, interest in CQ has resurged owing to its documented broad‐spectrum antiviral activity against multiple viral families (Han et al. 2019). As a weakly basic compound, CQ elevates the pH within intracellular organelles, leading to the disruption of their structural integrity and physiological functions, which in turn interferes with viral entry, replication and infection (Wei et al. 2020). Similarly, hydroxychloroquine, dibucaine and amodiaquine demonstrated significant antiviral efficacy (Musharrafieh et al. 2020; Piconi et al. 2011; Sakurai et al. 2018) (Figure S11a). Accumulating evidence has underscored the critical influence of pH modulation on biomolecular condensate formation (Fujioka et al. 2020; King et al. 2024; Ray et al. 2020). Notably, numerous PS regulators possess side chains containing weakly basic amine groups, which are critical for modulating the condensate dynamics (Huang et al. 2023; Jack et al. 2021; Zhao et al. 2021) (Figure S11b). Based on this insight, a series of quinoline derivatives containing basic amine side chains was rationally designed and synthesised to target virus and host protein interactions with the aim of disrupting or modulating the assembly of N protein particles (Figure S11c).

**FIGURE 8:**
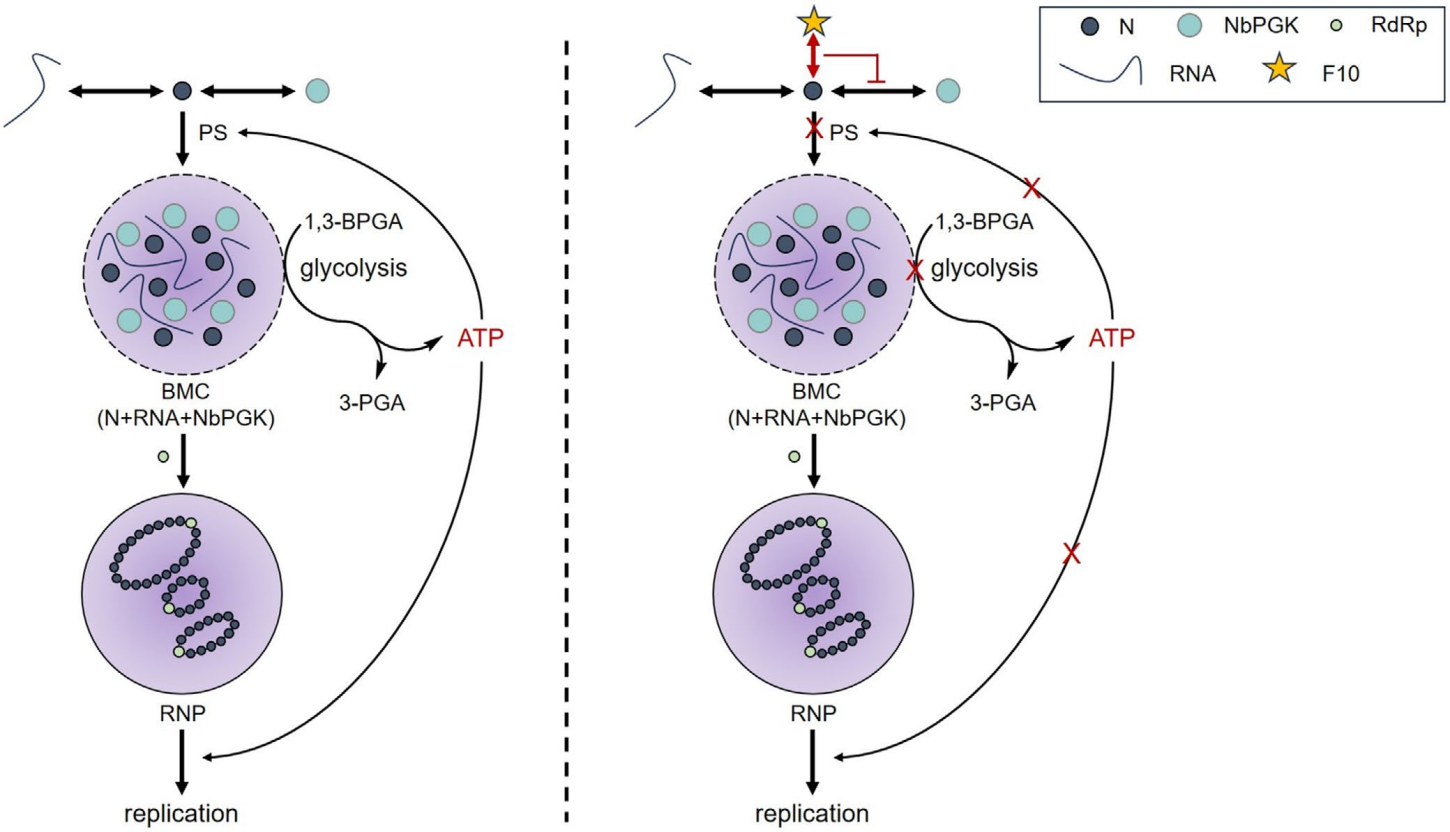
A proposed working model illustrating how the TSWV directly recruits the host NbPGK into the condensates of N protein and RNA via PS to reprogramme the glycolytic pathway, thereby generating ATP not only to supply energy for viral replication via ribonucleoprotein complexes but also to act as a promoter to regulate the PS network facilitating condensate formation. **F10** specifically targets the sites Arg94, Lys192 and Gly228 on TSWV N to inhibit the formation of BMC and suppress viral replication.

The N protein forms highly motile cytoplasmic IBs that travel along the actin filaments (Feng et al. 2013). Compound **F10** effectively inhibited the formation of N protein particles, which are essential for viral replication and assembly. Condensate formation contributes to the stabilisation of the N protein, rendering it less susceptible to degradation within plant cells (Zan et al. 2025). The TSWV N protein plays a critical role in viral replication and transcription by assembling viral RNA into RNP (Li et al. 2015). Treatment with **F10** significantly suppressed RNP replication, as indicated by a marked reduction in fluorescence intensity in *N. benthamiana* leaves (Figure 5b), suggesting that **F10** likely targets the N protein to inhibit viral genome replication. Subsequently, we systematically assessed antiviral activity against TSWV. **F10** exhibited excellent antiviral efficacy (Tables S1 and S2, Figure S9). Identification of the N protein as a direct target of **F10** was further validated using the DARTS assay. Further investigations were conducted to elucidate the underlying mechanisms of action of **F10**. Molecular docking was performed using the N protein as the target and three key amino acid residues, R94, K192 and G228, that are critical for **F10** binding (Figure 6a). Functional analyses revealed that triple‐site mutation (R94A&K192A&G228A) significantly compromised RNP replication (Figure 6c), systemic infection (Figure 6f), and N protein accumulation (Figure 6g), highlighting the critical role of these residues in viral pathogenicity and their relevance to **F10** binding. In vivo assays revealed that N^R94A&K192A&G228A^‐YFP failed to exhibit detectable condensate formation in *N. benthamiana* leaf cells at 24 and 48 hpi (Figure 7a,b). This result indicates that the mutations severely compromised the capacity of the N protein to mediate condensate assembly. Interestingly, this phenotype closely resembled that observed following **F10** treatment, implying that **F10** may inhibit condensate formation by targeting these essential residues. Subsequently, we observed that **F10** treatment at 30, 36 and 42 hpi markedly disrupted the interaction between N^WT^ or N^R94A&K192A&G228A^ and NbPGK (Figure 7h,i,j), suggesting that **F10** dissociated the N‐NbPGK complex and released the sequestered NbPGK, thereby contributing to the restoration of host glycolytic homeostasis. These findings indicated that **F10** may exert its anti‐TSWV activity by specifically interfering with this critical host‐virus interaction (Figure 8).

In summary, we uncovered a novel mechanism by which TSWV hijacks the host glycolytic enzyme, NbPGK, to reprogramme host metabolism via PS, thereby promoting condensate formation and facilitating efficient viral replication. ATP generated through this reprogramming plays a dual role: it serves as an essential energy source for viral replication and acts in a non‐enzymatic manner to modulate the macromolecular interaction network within condensates, thereby enhancing their formation. Furthermore, we rationally designed and synthesised a small molecule PS modulator, **F10**, which targeted key residues of the N protein (Arg94, Lys192 and Gly228), disrupted its interaction with NbPGK, and effectively suppressed condensate formation. This work not only advances our mechanistic understanding of how plant viruses exploit host metabolism to support their proliferation, but also provides a strategic protocol for the development of antiviral agrochemicals targeting PS‐driven processes in plant pathology.

## 4 Materials and Methods

### 4.1 Materials and Virus Sources

Seeds of *N. benthamiana* and *N. glutinosa* were preserved in our laboratory. The TSWV lettuce isolate was maintained in *N. benthamiana*, and the infected leaves were stored at −80°C. *N. glutinosa* was used to test the bioactivity of the compounds. The VSRs expression vectors, full‐length infectious clone vectors L_(+)__opt_, M_(−)__opt_ and SR_(+)eGFP_, and plasmid pCambia2300‐N‐YFP were graciously provided by Professor Xiaorong Tao (Nanjing Agricultural University).

### 4.2 CO‐IP MS

We referred to previously published Co‐IP MS data from our group (Wang, Luo, et al. 2025). Based on peptide scores and relevant literature reports (Prasanth et al. 2017), several candidate proteins were selected for preliminary validation. Gene‐specific primers were designed (Table S4), and the transcript levels of these candidates were quantified by qRT‐PCR in *N. benthamiana* leaves transiently expressing N^WT^ relative to healthy controls.

### 4.3 LCA

The plasmids JW771‐nLUC‐N (nLUC‐N), JW771‐nLUC‐N^R94A&K192A&G228A^ (nLUC‐N^R94A&K192A&G228A^) and JW772‐cLUC‐PGK (cLUC‐PGK) were constructed by homologous recombination. After sequence verification, the constructs were transformed into the *Agrobacterium tumefaciens* strain GV3101 (pSoup‐p19), along with the empty vector controls JW771‐nLUC (nLUC) and JW772‐cLUC (cLUC). *Agrobacterium* cultures were adjusted to an optical density at 600 nm (OD_600_) of 1.0 in infiltration buffer. Equal volumes were mixed and infiltrated into the 5–6 leaf‐stage of *N. benthamiana* plants with uniform growth. Leaf images were captured using an imager for detection and subsequently processed using Image Lab and Adobe Illustrator software. Primers used in the experiments are listed in Table S5.

### 4.4 BiFC

Plasmids pCV‐nYFP‐N (nYFP‐N), pCV‐nYFP‐N^R94A&K192A&G228A^ (nYFP‐N^R94A&K192A&G228A^) and pCV‐cYFP‐PGK (cYFP‐PGK) were constructed by homologous recombination. After sequence verification, the constructs were transformed into *Agrobacterium tumefaciens* strain GV3101 (pSoup‐p19), including empty vector controls, nYFP and cYFP. Agroinfiltration buffer was prepared at an optical density (OD_600_) of 1.0. Equal volumes of the suspensions were mixed and infiltrated into 5–6 leaf‐stage *N. benthamiana* plants under uniform growth conditions. Fluorescence signals were observed using confocal laser microscopy 48–60 hpi. The primers used in the experiments are listed in Table S5.

### 4.5 Protein–Protein Interaction In Vitro

The purified sfGFP‐N^WT^ and sfGFP‐NbPGK proteins were concentrated and then diluted to 10 μM using desalting buffer (150 mM NaCl and 20 mM Tris–HCl, pH 7.5). Equal volumes (10 μL each) of sfGFP‐N^WT^ and sfGFP‐NbPGK were mixed, followed by the addition of polyethylene glycol 8000 (PEG8000) to a final concentration of 0%, 2%, 4% or 8%. The mixtures were incubated for 10 min and observed under a confocal microscope.

### 4.6 Virus‐Induced Gene Silencing (VIGS)

Agrobacterium cultures harbouring pTRV2‐PGK and pTRV1 (OD_600_ = 0.5) were mixed in a 1:1 volumetric ratio and co‐infiltrated into *N. benthamiana* plants at the 3–5 leaf stage. The negative control consisted of plants infiltrated with pTRV1 and pTRV2, whereas the positive control (pTRV2‐PDS + pTRV1) exhibited characteristic leaf photobleaching at 10 dpi. The silencing efficiency of PGK was assessed by quantitative real‐time PCR (qRT‐PCR) at 10 dpi. The TSWV N^WT^ agroinfection suspensions contained genomic components (L_(+)opt_, M_(−)opt_, and SR_(+)eGFP_ at a final OD_600_ of 0.2, and VSRs at a final OD_600_ of 0.1). At 10 dpi, the relative transcript levels of the N gene and the protein accumulation of the N protein in *N. benthamiana* were determined by qRT‐PCR and Western blot analysis, respectively. Primer sequences utilised for VIGS are provided in Table S5.

### 4.7 Assessment of the Role of Glycolytic Pathway in N Protein Condensate Formation

To evaluate the role of the glycolytic pathway in N protein‐mediated condensate formation, *N. benthamiana* leaves were infiltrated with *Agrobacterium tumefaciens* (OD_600_ = 0.6) harbouring p2300‐N^WT^‐YFP. At 12 hpi, the right side of the same leaf was injected with working solutions of 2‐DG at final concentrations of 10, 15, 20 and 25 mM. As a control, the left side was injected with an equal volume of deionised water. Leaf tissues were collected at 24 and 48 hpi for confocal laser scanning microscopy to observe N protein condensate formation. All experiments were independently repeated at least three times.

### 4.8 MST and BLI Assays

The *K*D between compound **F10** and both TSWV N^WT^ and TSWV N^R94A&K192A&G228A^ were determined using MST. Similarly, The *K*D between **F10** and N^WT^ or N^R94A/K192A/G228A^ were determined using the Octet BLI Discovery system (version 12.2, ForteBio, San Francisco, USA), following previously published protocols (Zan et al. 2025).

### 4.9 Plasmid Construction

Using SR_(+)eGFP_^WT^ as a template, linearized vectors and target fragments were amplified by reverse transcription polymerase chain reaction. The mutant plasmids SR_(+)eGFP_^Mut^(SR_(+)eGFP_^R94A^, SR_(+)eGFP_^K192A^, SR_(+)eGFP_^G228A^ and SR_(+)eGFP_^R94A&K192A&G228A^) were constructed via homologous recombination. The linearized vector was amplified from pCambia2300‐N‐YFP, and the N^R94A&K192A&G228A^ target fragment was amplified from the SR_(+)eGFP_^R94A&K192A&G228A^ plasmid to construct the plasmid pCambia2300‐N^R94A&K192A&G228A^‐YFP. The two fragments were assembled using homologous recombination. The pAN580‐N‐eGFP plasmid was synthesised by Biorun Biosciences Co. Ltd. (Wuhan, China). All the recombinant constructs were verified by DNA sequencing. The primer sequences used for plasmid construction are listed in Table S5.

### 4.10 Antiviral Activity Assay

TSWV was propagated in *N. benthamiana* and purified following established methodologies described in the literature. The antiviral activities of compounds **F1–F37** against TSWV were evaluated according to published biochemical and phenotypic assay protocols (Zu et al. 2021).

### 4.11 Molecular Docking

Based on compound **F10**, which exhibited the most potent anti‐TSWV activity, potential key amino acid binding sites on the crystal structure of the TSWV N protein (PDB ID: 5IP1) were predicted and analysed. Molecular dynamics (MD) simulations were performed using the molecular docking results.

### 4.12 Drug Treatment

At 24 hpi, using the minigenome replication system (L_(+)opt_ + SR_(+)eGFP_ + VSRs) in *N. benthamiana*, compound **F10** was applied to the left side of the leaf. **F10** (1 mg) was dissolved in 30 μL DMSO and diluted with 10 mL ddH_2_O to a final concentration of 100 μg/mL. The eGFP fluorescence was examined at 5 dpi using a fluorescence microscope. Similarly, at 18 hpi with Agrobacterium harbouring the N‐YFP, the **F10** solution (100 μg/mL) was infiltrated into the upper left region of the leaf, while a control solution (30 μL DMSO in 10 mL ddH_2_O) was infiltrated into the upper right region. Treated leaves were imaged at 24 and 48 hpi using a confocal laser scanning microscope.

## Author Contributions

Guangcheng Zu conducted most of the experiments. Zhifu Xing contributed to some experiments. Guangcheng Zu, Jiao Li and Huan Wu analysed the data. Guangcheng Zu, Tangbing Yang, Qiangsheng Ge and Yanju Wang drafted the manuscript. Runjiang Song and Baoan Song designed the experiments, supervised the study and wrote and revised the paper. All authors read and approved its content.

## Conflicts of Interest

The authors declare no conflicts of interest.

## Supporting information


**Movie S1:** pbi70529‐sup‐0001‐MovieS1.avi.

**Movie S2:** pbi70529‐sup‐0002‐MovieS2.avi.

**Movie S3:** pbi70529‐sup‐0003‐MovieS3.avi.

**Movie S4:** pbi70529‐sup‐0004‐MovieS4.avi.

**Movie S5:** pbi70529‐sup‐0005‐MovieS5.avi.

**Movie S6:** pbi70529‐sup‐0006‐MovieS6.avi.

**Movie S7:** pbi70529‐sup‐0007‐MovieS7.avi.

**Data S1:** pbi70529‐sup‐0008‐Supinfo.docx.

## Data Availability

The data that supports the findings of this study are available in the Supporting Information of this article.
