# Supplementary material for: Tomato Spotted Wilt Virus Reprogrammes Host Glycolysis to Facilitate Proliferation by a Phase‐Separated Co‐Aggregate of Nucleocapsid Protein and Phosphoglycerate Kinase
Source: Plant Biotechnol J. 2026 Jan 6;24(5):2826–42. doi: 10.1111/pbi.70529 (PMC13110190; doi:10.1111/pbi.70529)
Supplement: Supplementary file 8 — Data S1: pbi70529‐sup‐0008‐Supinfo.docx. [file PBI-24-2826-s007.docx]

**Supporting Information**

**Tomato spotted wilt virus reprograms host glycolysis to facilitate proliferation by a phase-separated co-aggregate of nucleocapsid protein and phosphoglycerate kinase**

Guangcheng Zu^1^, Zhifu Xing^1^, Jiao Li^1^, Tangbing Yang^1^, Huan Wu^1^, Qiangsheng Ge^1^, Yanju Wang^1^, Baoan Song ^1,^ *, Runjiang Song ^1,^ *

^1^Current address: State Key Laboratory of Green Pesticide, Center for R&D of Fine Chemicals of Guizhou University, Guiyang 550025, P. R. China.

*Address of the corresponding author

Fax: 0086-851-83622211; E-mail: [basong@gzu.edu.cn](mailto:basong@gzu.edu.cn), songrj@gzu.edu.cn.

Contents

[1. Results 1](#_Toc212764059)

[2. Table S. Primer sequence of PCR and qRT-PCR. 11](#_Toc212764060)

[3. Materials and methods 14](#_Toc212764061)

[4. Characterizations of products 19](#_Toc212764062)

[5. ^1^H, ^13^C NMR and HRMS data 35](#_Toc212764063)

[6. References 76](#_Toc212764064)

**1.** **Results**


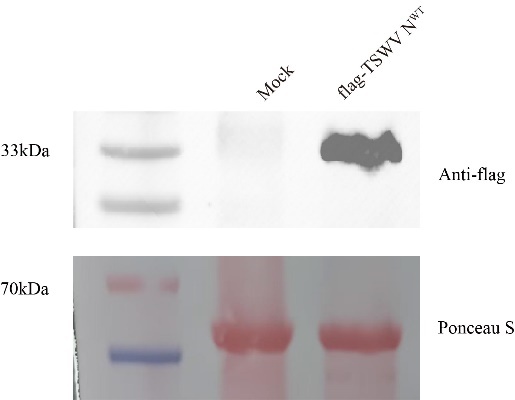


**Figure S1** The expression levels of Flag-TSWV N^WT^ was detected by Western blot at 48 hours post inoculation (hpi).


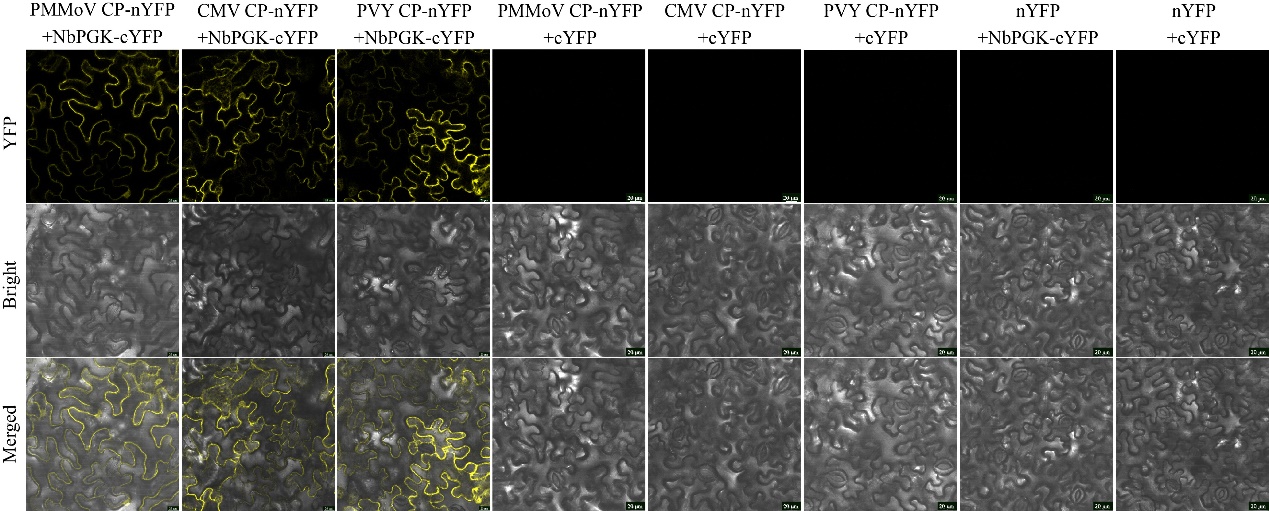


**Figure S2** The interactions between the coat proteins of Pepper mild mottle virus (PMMoV, *Tobamovirus*), Cucumber mosaic virus (CMV, *Cucumovirus*), and Potato virus Y (PVY, *Potyvirus*) and NbPGK were assessed using BiFC assays.


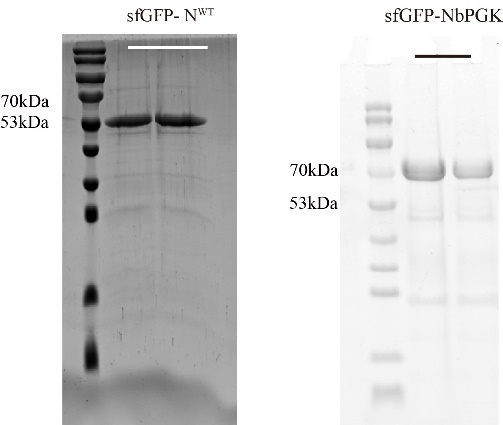


**Figure S3** The purified protein sfGFP-N^WT^ and sfGFP-NbPGK were identified by SDS-PAGE.


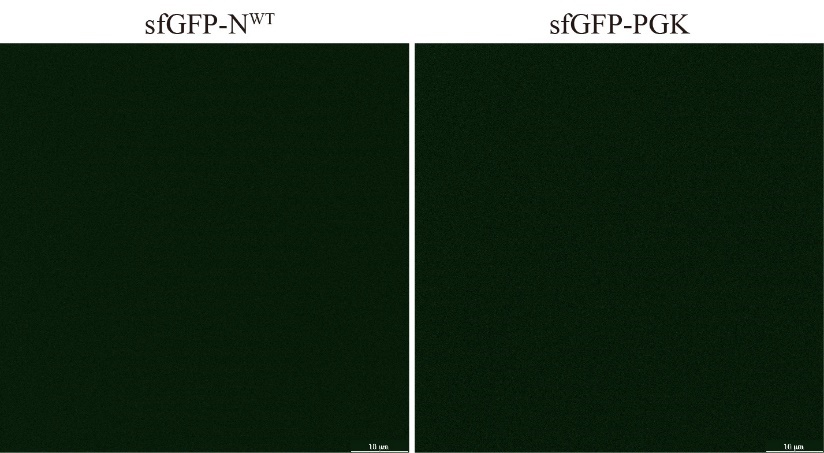


**Figure S4** confocal microscopy revealed that neither sfGFP-N^WT^ nor sfGFP-NbPGK formed condensates when incubated individually.


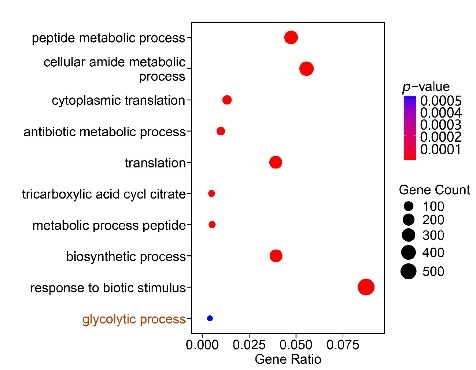


**Figure S5** GO pathway enrichment analysis revealed significant enrichment of the glycolysis pathway in CK and N^WT^ samples.


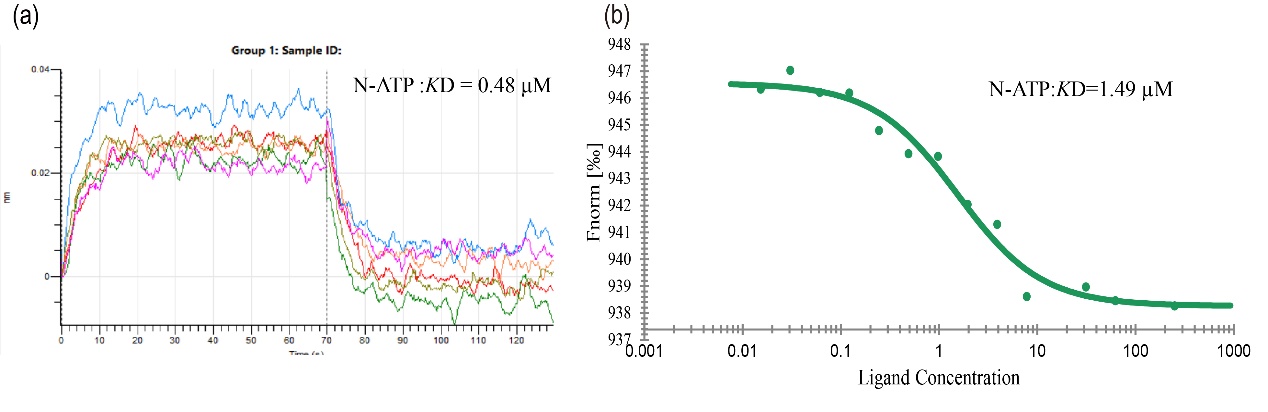


**Figure S6** Binding affinity measurement between ATP and the TSWV N protein. (a) The binding affinity between ATP and the TSWV N protein was quantitatively assessed by biolayer interferometry (BLI). (b) The binding affinity between ATP and the TSWV N protein was quantitatively assessed by microscale thermophoresis (MST).


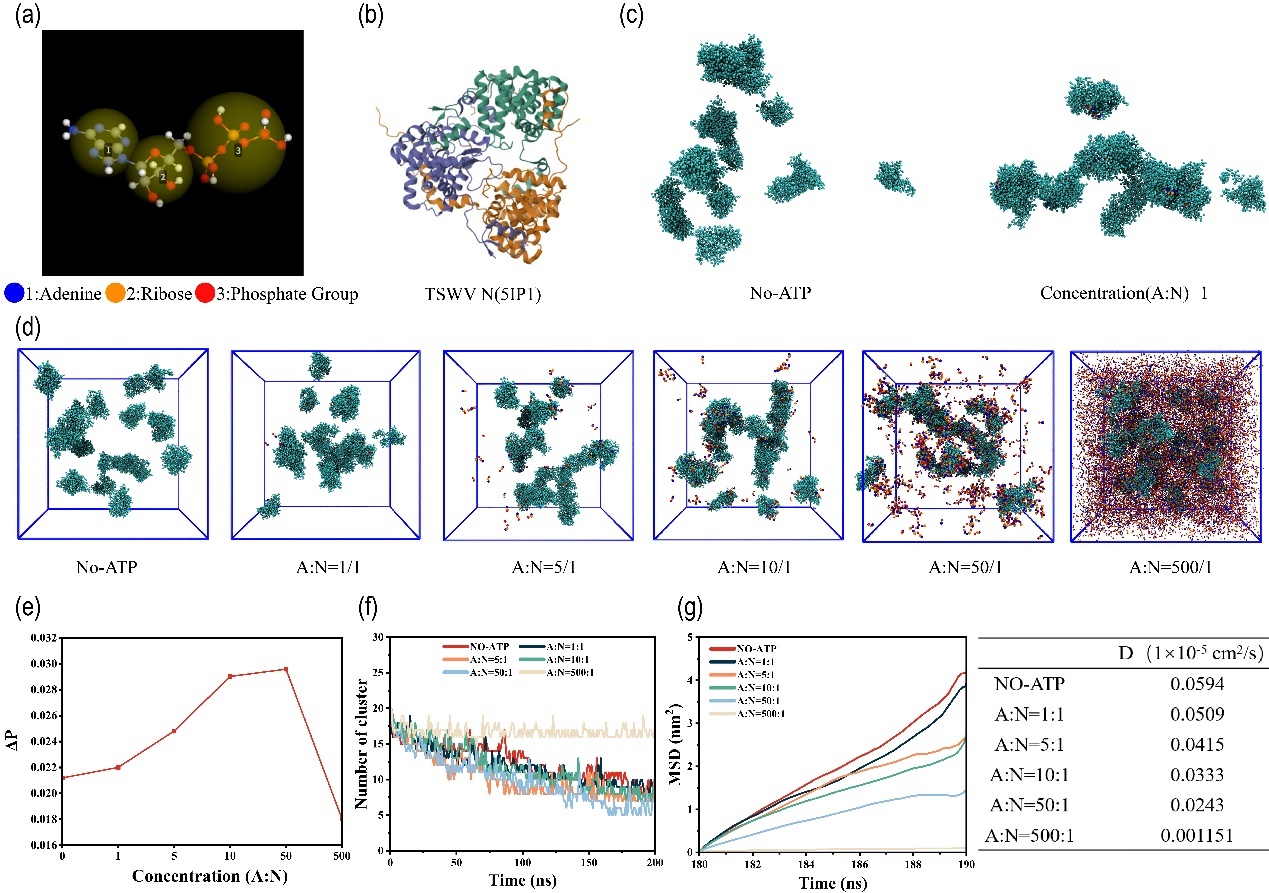


**Figure S7** ATP promotes the phase separation of N through non-enzymatic interactions. (a) Coarse-grained mapping of ATP into adenine, ribose, and phosphate groups. (b) Asymmetric trimeric structure of the TSWV N protein (PDB: 5IP1). (c, d) Conformations obtained from simulations at different ATP concentrations. Blue rectangles indicate the simulation boxes. (e) Changes in ΔP values at different ATP concentrations. The Y-axis represents the difference between the maximum and minimum fractions of N beads (ΔP), which reflects the degree of phase separation. Higher ΔP values indicate stronger condensate formation. The X-axis shows the ATP-to-N concentration ratio (A:N). (f) Number of condensate clusters formed at different ATP concentrations. The X-axis represents simulation time, and the Y-axis represents the number of condensate clusters identified in each system. (g) Mean squared displacement (MSD) curves of N at different ATP concentrations. The MSD values were calculated from the trajectories of N beads in each simulation system to evaluate molecular mobility. The X-axis represents simulation time, and the Y-axis indicates the MSD of N.


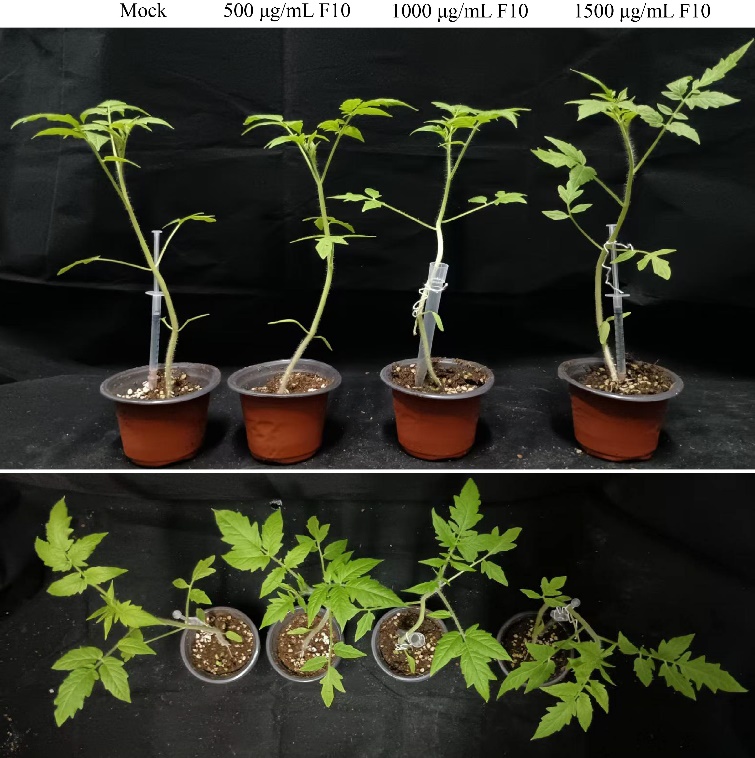


**Figure S8** Assessment of the phytotoxicity of the small molecule **F10** on tomato plants.


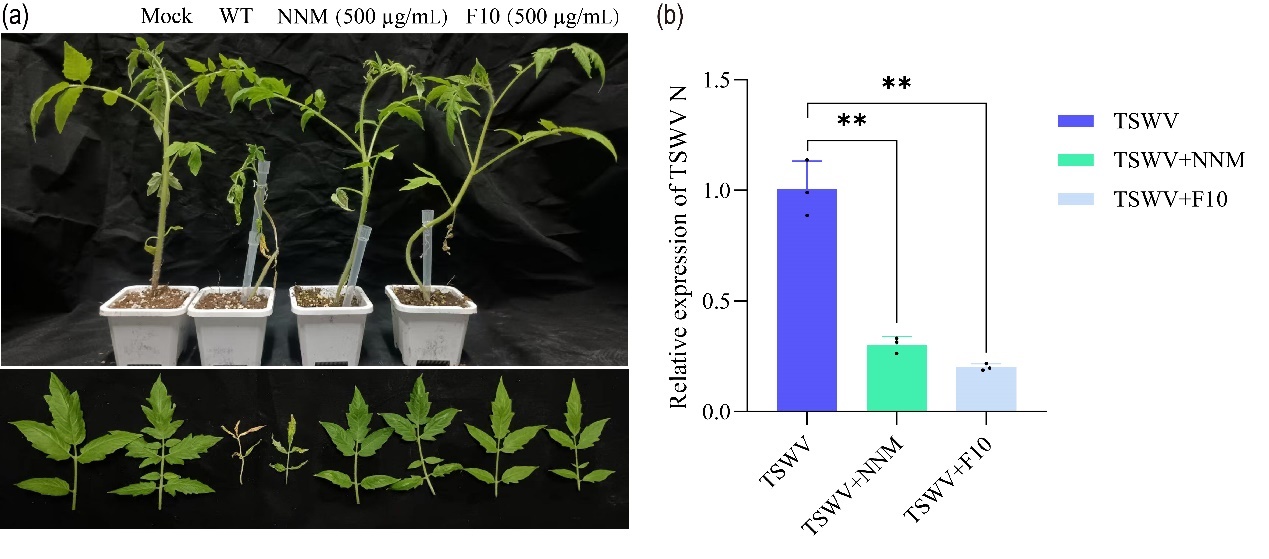


**Figure S9** Evaluation of the antiviral activity of the small molecule **F10** against TSWV using tomato plants as the experimental system. (a) Phenotypic changes were observed on the 21st day post-virus inoculation. (b) Relative expression levels of the N gene were analyzed across different treatment groups.


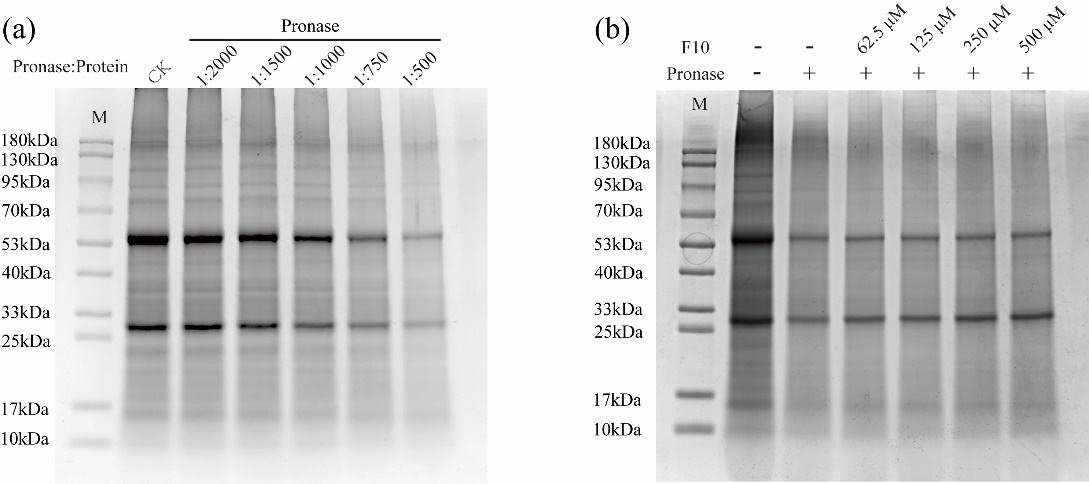


**Figure S10** DARTS assay. (a) Optimization of pronase concentration for proteolytic digestion. (b**)** incubation of total protein lysates with increasing concentrations of the compound for gradient analysis.


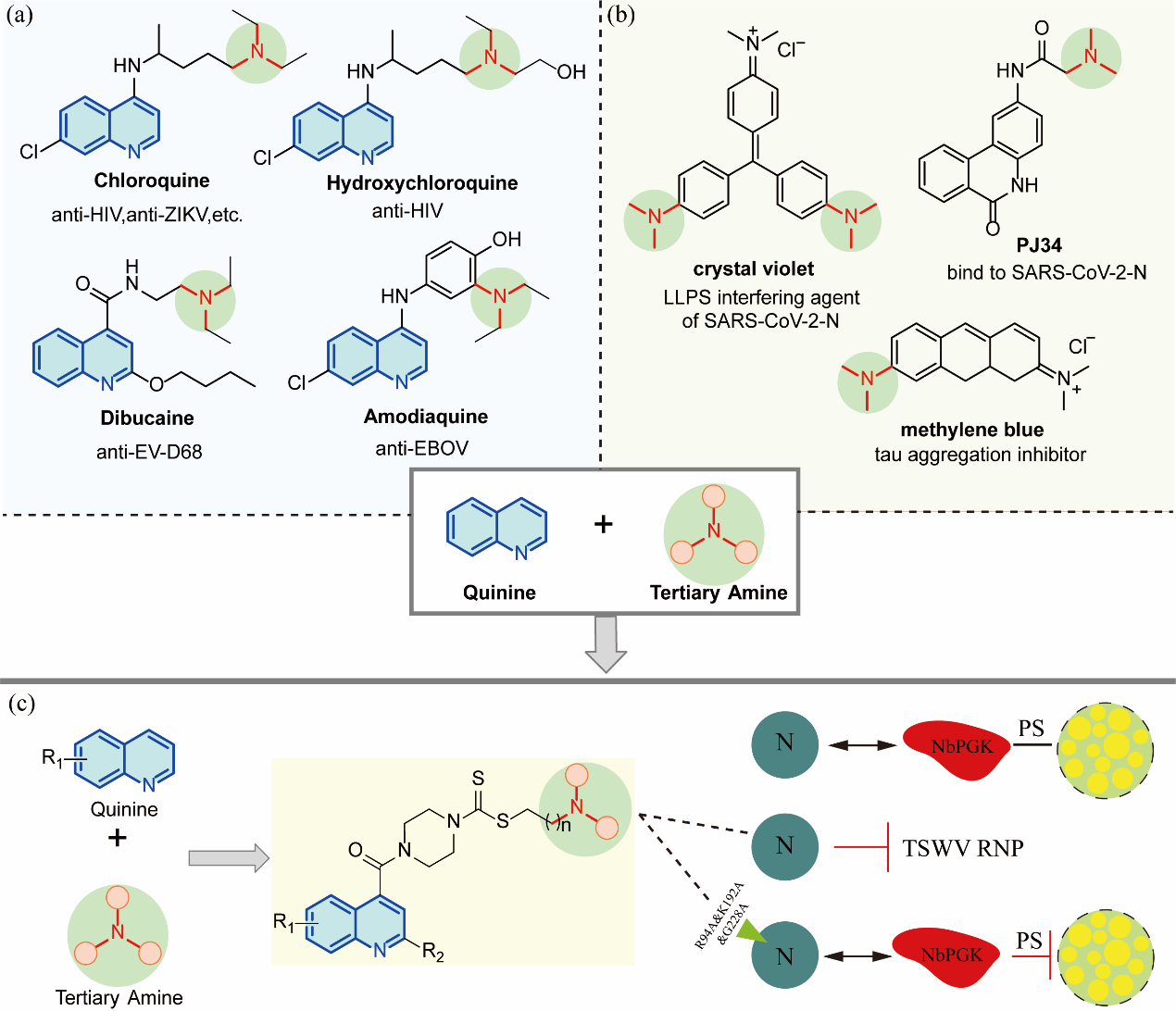


**Figure S11** Design strategy for biomolecular condensate inhibiting phytovirucides. (a) Structures of quinoline-based compounds with antiviral activity. (b) Structures of reported aggregation modulators containing tertiary amine groups. (c) Rational design of anti-TSWV compounds in this study aimed at inhibiting phase separated (PS) condensates.

**Scheme S1** Synthetic route to the title compounds **F1**-**F37**.

**Table S1** Anti-TSWV activity of target compounds **F1-F37** at 500 µg/mL

|  | | | | | | | |
| --- | --- | --- | --- | --- | --- | --- | --- |
| compound | R_1_ | R_2_ | n | R_3_ | Curative^a^(%) effect(%) | Protective^a^(%) effect(%) | Inactive^a^(%) effect(%) |
| F1 | 6-CH_3_ | -CH_3_ | 1 | -O(CH_2_)_2_OCH_3_ | 55.73±3.23 | 55.08±2.87 | 63.44±3.99 |
| F2 | 6-CH_3_ | -CH_3_ | 2 | -O(CH_2_)_2_OCH_3_ | 47.08±4.52 | 51.37±3.01 | 69.42±1.11 |
| F3 | 6-CH_3_ | -CH_2_CH_3_ | 2 | -O(CH_2_)_2_OCH_3_ | 54.56±3.93 | 54.24±3.12 | 73.65±4.39 |
| F4 | 6-CH_3_ | -CH_3_ | 1 | -O(CH_2_)_3_OCH_3_ | 57.00±5.28 | 45.57±2.65 | 59.80±4.34 |
| F5 | 6-CH_3_ | -CH_2_CH_3_ | 1 | -O(CH_2_)_3_OCH_3_ | 52.50±3.72 | 40.44±3.23 | 64.28±2.30 |
| F6 | 6-CH_3_ | -CH_3_ | 2 | -O(CH_2_)_3_OCH_3_ | 39.73±5.54 | 34.07±4.42 | 39.65±4.98 |
| F7 | 6-CH_3_ | -CH_2_CH_3_ | 2 | -O(CH_2_)_3_OCH_3_ | 58.38±3.03 | 42.89±3.89 | 63.11±3.87 |
| F8 | 6-F | -CH_3_ | 1 | -O(CH_2_)_2_OCH_3_ | 51.08±3.73 | 53.06±2.27 | 60.58±4.09 |
| F9 | 6-F | - CH_2_CH_3_ | 1 | -O(CH_2_)_2_OCH_3_ | 56.17±2.24 | 55.81±3.02 | 66.79±2.31 |
| F10 | 6-F | -CH_3_ | 2 | -O(CH_2_)_2_OCH_3_ | 60.11±4.53 | 63.89±3.57 | 74.09±2.92 |
| F11 | 6-F | -CH_2_CH_3_ | 2 | -O(CH_2_)_2_OCH_3_ | 59.87±5.26 | 60.92±1.42 | 61.83±3.50 |
| F12 | 6-F | -CH_3_ | 1 | -O(CH_2_)_3_OCH_3_ | 43.08±5.77 | 39.37±5.39 | 42.97±3.44 |
| F13 | 6-F | -CH_2_CH_3_ | 1 | -O(CH_2_)_3_OCH_3_ | 56.06±6.38 | 58.92±6.77 | 55.18±4.62 |
| F14 | 6-F | -CH_3_ | 2 | -O(CH_2_)_3_OCH_3_ | 59.46±4.38 | 59.41±4.73 | 69.55±4.71 |
| F15 | 6-F | -CH_2_CH_3_ | 2 | -O(CH_2_)_3_OCH_3_ | 60.11±3.66 | 56.15±5.43 | 71.32±6.23 |
| F16 | 6-Cl | -CH_3_ | 1 | -O(CH_2_)_2_OCH_3_ | 54.91±5.59 | 38.46±6.06 | 64.92±1.19 |
| F17 | 6-Cl | -CH_2_CH_3_ | 1 | -O(CH_2_)_2_OCH_3_ | 56.22±3.21 | 53.27±5.11 | 49.26±2.55 |
| F18 | 6-Cl | -CH_3_ | 2 | -O(CH_2_)_2_OCH_3_ | 52.39±3.47 | 57.83±4.49 | 65.66±4.22 |
| F19 | 6-Cl | -CH_2_CH_3_ | 2 | -O(CH_2_)_2_OCH_3_ | 56.77±4.47 | 50.60±3.82 | 72.50±2.78 |
| F20 | 6-Cl | -CH_3_ | 1 | -O(CH_2_)_3_OCH_3_ | 47.44±4.06 | 52.72±3.68 | 60.56±4.20 |
| F21 | 6-Cl | -CH_2_CH_3_ | 1 | -O(CH_2_)_3_OCH_3_ | 46.28±3.50 | 51.61±2.84 | 56.05±4.24 |
| F22 | 6-Cl | -CH_3_ | 2 | -O(CH_2_)_3_OCH_3_ | 54.60±0.67 | 63.92±4.42 | 56.81±8.55 |
| F23 | 6-Cl | -CH_2_CH_3_ | 2 | -O(CH_2_)_3_OCH_3_ | 53.85±4.38 | 55.97±2.73 | 70.38±2.04 |
| F24 | 7-Cl | -CH_3_ | 1 | -O(CH_2_)_2_OCH_3_ | 57.58±4.28 | 53.73±6.90 | 69.85±2.40 |
| F25 | 7-Cl | -CH_2_CH_3_ | 2 | -O(CH_2_)_2_OCH_3_ | 49.86±1.88 | 53.48±6.15 | 62.52±3.90 |
| F26 | 7-Cl | -CH_3_ | 2 | -O(CH_2_)_2_OCH_3_ | 55.37±2.28 | 62.12±5.41 | 65.13±3.86 |
| F27 | 7-Cl | -CH_3_ | 1 | -O(CH_2_)_3_OCH_3_ | 41.67±5.73 | 55.30±6.65 | 67.10±2.76 |
| F28 | 7-Cl | -CH_2_CH_3_ | 1 | -O(CH_2_)_3_OCH_3_ | 54.79±2.88 | 56.83±3.10 | 73.07±3.41 |
| F29 | 7-Cl | -CH_3_ | 2 | -O(CH_2_)_3_OCH_3_ | 42.52±2.75 | 41.81±3.33 | 51.52±5.06 |
| F30 | 7-Cl | -CH_2_CH_3_ | 2 | -O(CH_2_)_3_OCH_3_ | 59.22±4.43 | 55.59±3.30 | 70.31±4.17 |
| F31 | H | -CH_3_ | 1 | -O(CH_2_)_2_OCH_3_ | 47.67±4.09 | 32.65±4.30 | 38.63±4.02 |
| F32 | H | -CH_3_ | 2 | -O(CH_2_)_2_OCH_3_ | 45.59±3.74 | 46.23±2.61 | 51.37±3.49 |
| F33 | H | -CH_2_CH_3_ | 2 | -O(CH_2_)_2_OCH_3_ | 37.71±3.16 | 41.88±6.54 | 46.51±3.60 |
| F34 | H | -CH_3_ | 1 | -O(CH_2_)_3_OCH_3_ | 44.16±3.00 | 45.42±3.94 | 54.78±2.55 |
| F35 | H | -CH_2_CH_3_ | 1 | -O(CH_2_)_3_OCH_3_ | 38.80±4.05 | 33.26±2.64 | 41.83±4.47 |
| F36 | H | -CH_3_ | 2 | -O(CH_2_)_3_OCH_3_ | 51.33±4.02 | 52.86±2.82 | 67.14±4.92 |
| F37 | H | -CH_2_CH_3_ | 2 | -O(CH_2_)_3_OCH_3_ | 57.69±2.40 | 50.67±4.88 | 65.49±3.17 |
| Ningnanmycin^b^ |  |  |  |  | 56.95±3.29 | 60.18±3.54 | 71.11±2.94 |

Notes:^a^ Average of three replicates. ^b^ Ningnanmycin used for comparison of activity.

**Table S2** Anti-TSWV activity assay *in vivo*

| compound |  | | | | EC_50_ for protection effect^a^(μg/mL) | EC_50_ for Inactivation effect^a^(μg/mL) |
| --- | --- | --- | --- | --- | --- | --- |
|  | R_1_ | R_2_ | n | R_3_ |  |  |
| F1 | 6-CH_3_ | -CH_3_ | 1 | -O(CH_2_)_2_OCH_3_ | 293.91±7.85 | 199.51±6.29 |
| F2 | 6-CH_3_ | -CH_3_ | 2 | -O(CH_2_)_2_OCH_3_ | 466.45±5.94 | 148.31±6.65 |
| F3 | 6-CH_3_ | -CH_2_CH_3_ | 2 | -O(CH_2_)_2_OCH_3_ | 350.86±11.64 | 154.95±8.99 |
| F4 | 6-CH_3_ | -CH_3_ | 1 | -O(CH_2_)_3_OCH_3_ | 556.10±7.26 | 293.01±10.4 |
| F5 | 6-CH_3_ | -CH_2_CH_3_ | 1 | -O(CH_2_)_3_OCH_3_ | 660.61±11.69 | 199.17±5.15 |
| F7 | 6-CH_3_ | -CH_2_CH_3_ | 2 | -O(CH_2_)_3_OCH_3_ | 626.05±15.84 | 212.59±8.14 |
| F8 | 6-F | -CH_3_ | 1 | -O(CH_2_)_2_OCH_3_ | 377.82±9.45 | 236.65±6.93 |
| F9 | 6-F | -CH_2_CH_3_ | 1 | -O(CH_2_)_2_OCH_3_ | 286.45±8.51 | 162.56±8.16 |
| F10 | 6-F | -CH_3_ | 2 | -O(CH_2_)_2_OCH_3_ | 143.77±7.98 | 81.83±8.26 |
| F11 | 6-F | -CH_2_CH_3_ | 2 | -O(CH_2_)_2_OCH_3_ | 203.85±7.14 | 252.89±8.97 |
| F13 | 6-F | -CH_2_CH_3_ | 1 | -O(CH_2_)_3_OCH_3_ | 152.72±9.34 | 234.72±8.30 |
| F14 | 6-F | -CH_3_ | 2 | -O(CH_2_)_3_OCH_3_ | 184.68±7.78 | 133.64±8.49 |
| F15 | 6-F | -CH_2_CH_3_ | 2 | -O(CH_2_)_3_OCH_3_ | 174.58±8.68 | 145.80±5.76 |
| F16 | 6-Cl | -CH_3_ | 1 | -O(CH_2_)_2_OCH_3_ |  | 199.10±9.31 |
| F17 | 6-Cl | -CH_2_CH_3_ | 1 | -O(CH_2_)_2_OCH_3_ | 339.0±10.44 | 495.32±9.16 |
| F18 | 6-Cl | -CH_3_ | 2 | -O(CH_2_)_2_OCH_3_ | 277.75±8.96 | 196.72±10.94 |
| F19 | 6-Cl | -CH_2_CH_3_ | 2 | -O(CH_2_)_2_OCH_3_ | 434.68±7.81 | 127.79±9.12 |
| F20 | 6-Cl | -CH_3_ | 1 | -O(CH_2_)_3_OCH_3_ | 374.51±6.67 | 240.41±8.64 |
| F21 | 6-Cl | -CH_2_CH_3_ | 1 | -O(CH_2_)_3_OCH_3_ | 399.50±9.50 | 324.09±7.56 |
| F22 | 6-Cl | -CH_3_ | 2 | -O(CH_2_)_3_OCH_3_ | 191.74±6.29 | 340.04±8.06 |
| F23 | 6-Cl | -CH_2_CH_3_ | 2 | -O(CH_2_)_3_OCH_3_ | 277.84±10.94 | 151.05±7.24 |
| F24 | 7-Cl | -CH_3_ | 1 | -O(CH_2_)_2_OCH_3_ | 361.40±8.43 | 155.66±10.54 |
| F25 | 7-Cl | -CH_3_ | 2 | -O(CH_2_)_2_OCH_3_ | 349.99±12.28 | 195.79±6.48 |
| F26 | 7-Cl | -CH_2_CH_3_ | 2 | -O(CH_2_)_2_OCH_3_ | 200.09±8.18 | 209.39±11.39 |
| F27 | 7-Cl | -CH_3_ | 1 | -O(CH_2_)_3_OCH_3_ | 291.68±7.83 | 177.09±8.06 |
| F28 | 7-Cl | -CH_2_CH_3_ | 1 | -O(CH_2_)_3_OCH_3_ | 337.22±7.89 | 109.38±6.51 |
| F29 | 7-Cl | -CH_3_ | 2 | -O(CH_2_)_3_OCH_3_ |  | 455.50±9.79 |
| F30 | 7-Cl | -CH_2_CH_3_ | 2 | -O(CH_2_)_3_OCH_3_ | 309.22±9.24 | 139.15±8.18 |
| F32 | H | -CH_3_ | 2 | -O(CH_2_)_2_OCH_3_ | 564.63±8.26 | 475.57±10.33 |
| F33 | H | -CH_2_CH_3_ | 1 | -O(CH_2_)_2_OCH_3_ | 639.27±10.8 | 592.55±8.58 |
| F34 | H | -CH_3_ | 1 | -O(CH_2_)_3_OCH_3_ | 604.90±11.7 | 428.74±13.0 |
| F35 | H | -CH_2_CH_3_ | 1 | -O(CH_2_)_3_OCH_3_ |  | 641.11±11.89 |
| F36 | H | -CH_3_ | 2 | -O(CH_2_)_3_OCH_3_ | 366.60±7.22 | 139.89±9.81 |
| F37 | H | -CH_2_CH_3_ | 2 | -O(CH_2_)_3_OCH_3_ | 452.99±10.44 | 142.04±8.13 |
| Ningnanmycin^b^ |  | | | | 198.31±8.54 | 136.29±7.94 |

Notes:^a^ Average of three replicates. ^b^ Ningnanmycin used for comparison of activity.

**Table S3** Differential protein band identification list (iBAQ>0.5%)

| Accession | Gene | Mw (kDa) | Peptides | iBAQ-F10 | iBAQ-F10(%) | iBAQ-Pronase | iBAQ-Pronase(%) |
| --- | --- | --- | --- | --- | --- | --- | --- |
| F8UX84 | N | 28.94 | 13 | 110177 | 80.11 | 36976.8 | 85.43 |
| Q0PTH3 | N | 28.817 | 13 | 8561.8 | 6.23 | 1110.502 | 2.57 |
| Q0PSC7 | N | 28.817 | 13 | 4299.7 | 3.13 | 751.79 | 1.74 |
| Q0PSB8 | N | 28.817 | 13 | 4043.22 | 2.94 | 1643.15 | 3.80 |
| Q0PTG4 | N | 28.817 | 13 | 3072.79 | 2.23 | 320.71 | 0.74 |
| Q8JXK1 | N | 28.842 | 13 | 1713.91 | 1.25 | 84.566 | 0.20 |
| R9RVH5 | N | 28.984 | 14 | 1372 | 1.00 | 474.85 | 1.10 |
| A0A090AX59 | NSs | 52.285 | 24 | 1072.57 | 0.78 | 654.85 | 1.51 |
| D3K4A6 | N | 28.901 | 13 | 802.06 | 0.58 | 91.244 | 0.21 |

**2. Table S. Primer sequence of PCR and qRT-PCR.**

**Table** **S4** Primers used in qRT-PCR experiments

| primer | purpose | sequence (5′−3′) |
| --- | --- | --- |
| quantification of N gene-F | TSWV N qPCR | CTTGTCGAGGAAACCGGGAA |
| quantification of N gene-R |  | AGGTAAGCTACCTCCCAGCA |
| quantification of Actin gene-F |  | ATCGGAATGGAAGCTGCTGG |
| quantification of Actin gene-R |  | TCATCCTATCAGCAATGCCCG |
| quantification of *S. lycopersicum* Actin gene-F |  | GCTCCACCAGAGAGGAAATACAGT |
| quantification of *S. lycopersicum* Actin gene-R |  | CATACTCTGCCTTTGCAATCCA |
| quantification of NbPGK gene-F | NbPGK qPCR(VIGS) | GCTGCTGGAACAGAGGCTAT |
| quantification of NbPGK gene-F |  | ACGGCGGCAACAGAATCA |
| qNiben101Scf03856g00004-F | *N. benthamiana* genes | TGCTGCTGGAACAGAGGCTA |
| qNiben101Scf03856g00004-R |  | GCACCCCCTCCAGTTGAAAT |
| qNiben101Scf05912g01016-F |  | GCTACTTGGATTGCTGCGTG |
| qNiben101Scf05912g01016-R |  | AGCCCCAACAGCCTCATTAC |
| qNiben101Scf25430g00015-F |  | AGGGCGTACCAAAGTTCCTG |
| qNiben101Scf25430g00015-R |  | CACGATCACCACCCATCCAA |
| qNiben101Scf00753g00002-F |  | AACAAGCCATCTTCCCACGA |
| qNiben101Scf00753g00002-R |  | TCCACTGCGTCATTTCGACC |
| qNiben101Scf08651g04006-F |  | CGCATCTTTTGCCCCATTGA |
| qNiben101Scf08651g04006-R |  | CAGTCTTCTCCTCTGCGGTC |
| qNiben101Scf03777g00018-F |  | ATGGGCGTTGGATTGTGTCT |
| qNiben101Scf03777g00018-R |  | CTCACTCACAAGAAGCCCGT |
| qNiben101Scf04296g00024-F |  | GAGCAAACAACACGCGCTAT |
| qNiben101Scf04296g00024-R |  | AACCCACACAGTTCCAGCTC |
| qNiben101Scf25021g01016-F |  | TAGTTCATGCCCGTTTCGCC |
| qNiben101Scf25021g01016-R |  | CTCCCGCTTCATACCACACT |
| qNiben101Scf04812g00002-F |  | ATTTCGGGTTTCGGGCTTCA |
| qNiben101Scf04812g00002-R |  | GTTGGTGCGGGAGTAGGATT |
| qNiben101Scf01697g23018-F |  | CCAGAAGCCTGGTACTTCCG |
| qNiben101Scf01697g23018-R |  | TCACCAGAAACCACAAGCGT |
| qNiben101Scf04563g03007-F |  | GGGAGAAATATCGGACCCGC |
| qNiben101Scf04563g03007-R |  | AACCAGGAGCCACACCAAAT |
| qNiben101Scf00149g06013-F |  | ACAACAACCCACTTCGCTCT |
| qNiben101Scf00149g06013-R |  | GCTGGTGCCTTATCTGGTGT |
| qNiben101Scf01027g01014-F |  | TCGGCAAGCCTATGGAAGAC |
| qNiben101Scf01027g01014-R |  | AATTGCTGGCGTGTTTGCTT |
| qNiben101Scf08621g05005-F |  | GAGAGAGTGGTTGGCCGTAG |
| qNiben101Scf08621g05005-R |  | GCGCTTCCATTCACCTTGAC |

**Table S5** Primers used in PCR experiments

| primer | | sequence (5′−3′) | purpose |
| --- | --- | --- | --- |
| SR_(+)eGFP_^WT^ | MF83-F | GCATATAACAACTTCTACGATCATCATGTCTAAGGTTAAGCTCAC | Construction of SR_(+)eGFP_^WT^ |
|  | MF90-R | GATGATCGTAGAAGTTGTTATATGC |  |
| SR_(+)eGFP_^R94A^ | 94-F | GAGCCACTGACATGACCTTCGCTAGGCTTGATAGCTTGATC | Construction of SR_(+)eGFP_^Mut^ |
|  | 94-R | GATCAAGCTATCAAGCCTAGCGAAGGTCATGTCAGTGGCTC |  |
| SR_(+)eGFP_^K192A^ | 192-F | CACCAGGGAAGCCTTAGGAGCTGTTTGCACTGTGCTAAAAAG |  |
|  | 192-R | CTTTTTAGCACAGTGCAAACAGCTCCTAAGGCTTCCCTGGTG |  |
| SR_(+)eGFP_^G228A^ | 228-F | GCTCCAGCAATCCTAATGCTAAAGCTAGTATTGCTATGGAACATTAC |  |
|  | 228-R | GTAATGTTCCATAGCAATACTAGCTTTAGCATTAGGATTGCTGGAGC |  |
| p2300-N^WT^-YFP | ZT-F | CAAAACTTGCAGAACTTGCTTCTAGAATGGTGAGCAAGGGCGAGGAG | Construction of p2300-N^WT^-YFP |
|  | ZT-R | GTGAGCTTAACCTTAGACATGGTACCGAGCTCGCGAA |  |
| p2300-N^R94A&K192A&G228A^-YFP | PD-F | CTCGAGCTTTCGCGAGCTCGGTACCATGTCTAAGGTTAAGCTCAC | Construction of p2300-N^R94A&K192A&G228A^-YFP |
|  | PD-R | CCTCGCCCTTGCTCACCATTCTAGAAGCAAGTTCTGCAAGTTTTG |  |
| p2300-Flag-N^WT^ | Flag-F | CTCGGTACCATGGACTACAAGGACGACGACGACAAGATGTCTAAGGTTAAGCTCAC | Construction of p2300-Flag-N^WT^ |
|  | Flag-R | GACTCTAGATTAAGCAAGTTCTGCAAGTTTTG |  |
| p2300-Flag-N^R94A&K192A&G228A^-YFP | Flag-F | CTCGGTACCATGGACTACAAGGACGACGACGACAAGATGTCTAAGGTTAAGCTCAC | Construction of p2300-Flag-N^R94A&K192A&G228A^-YFP |
|  | Flag-R | GACTCTAGATTAAGCAAGTTCTGCAAGTTTTG |  |
| pCV-nYFP-N | nYFP-N-F | TCCGGAGTCGACGCACAGGGTACCATGTCTAAGGTTAAGCTCAC | Construction of BiFC vectors |
|  | nYFP-N-R | ATTCGAGCTCGCCTGGGGATCCTTAAGCAAGTTCTGCAAGTTTTGC |  |
| pCV-nYFP-N^R94A&K192A&G228A^ | nYFP-N-F | TCCGGAGTCGACGCACAGGGTACCATGTCTAAGGTTAAGCTCAC |  |
|  | nYFP-N-F | ATTCGAGCTCGCCTGGGGATCCTTAAGCAAGTTCTGCAAGTTTTGC |  |
| pCV-cYFP-NbPGK | cYFP-NbPGK-F | CGGAGTCGACGCACAGGGTACCATGGCAGTGAAGAAGAGTGTGGGA |  |
|  | cYFP-NbPGK-R | ATTCGAGCTCGCCTGGGGATCCTTAAGCATCATCGAGAGCAAGGA |  |
| JW771-N | JW771-N-F | ACACGGGGGACGAGCTCGGTACCATGTCTAAGGTTAAGCTCACTAAGG | Construction of LCA vectors |
|  | JW771-N-R | ACGCGTACGAGATCTGGTCGACTTAAGCAAGTTCTGCAAGTTTTGC |  |
| JW771-N^R94A&K192A&G228A^ | JW771-N-F | ACACGGGGGACGAGCTCGGTACCATGTCTAAGGTTAAGCTCACTAAGG |  |
|  | JW771-N-R | ACGCGTACGAGATCTGGTCGACTTAAGCAAGTTCTGCAAGTTTTGC |  |
| JW772-NbPGK | JW772-F | GTACGCGTCCCGGGGCGGTACCATGGCAGTGAAGAAGAGTGTGGGA |  |
|  | JW772-R | CTTGTAGTCCATTTGTTGGATCCTTAAGCATCATCGAGAGCAAGGA |  |
| TVR-NbPGK | TRV-F | AGTAAGGTTACCGAATTCCAAGGCCCAAGGATACTCTGTT | Construction of gene silencing vectors |
|  | TRV-R | ACGCGTGAGCTCGGTACCTTTTCAAACTCAAATACTCCC |  |
| sfGFP-TSWV N^WT^ | SF-GFP-TSWV-F | GGCGGGTATCACTCATGGCCTCGAGATGTCTAAGGTTAAGCTCAC | Construction of prokaryotic expression plasmids |
|  | SF-GFP-TSWV-R | GATCTCAATGATGGTGATGATGGTGTTAAGCAAGTTCTGCAAGTTTTG |  |
| sfGFP-TSWV N^R94A&K192A&G228A^ | SF-GFP-TSWV-F | GGCGGGTATCACTCATGGCCTCGAGATGTCTAAGGTTAAGCTCAC |  |
|  | SF-GFP-TSWV-R | GATCTCAATGATGGTGATGATGGTGTTAAGCAAGTTCTGCAAGTTTTG |  |
| sfGFP-NbPGK | sfGFP-NbPGK-F | GGCGGGTATCACTCATGGCCTCGAGATGGCAGTGAAGAAGAGTGT | Construction of prokaryotic expression plasmids |
|  | sfGFP-NbPGK-R | GATCTCAATGATGGTGATGATGGTGTTAAGCATCATCGAGAGCAAGGA |  |

**3. Materials and methods**

**Synthesis**

All solvents and reagents were obtained from commercial suppliers and used without further purification. Reaction progress was monitored by thin-layer chromatography (TLC) on silica gel GF254 plates under UV illumination. ^1^H and ^13^C NMR spectra were recorded on JEOL ECX-600 (JEOL, Tokyo, Japan), JEOL ECX-500 (JEOL, Tokyo, Japan) or Bruker Ascend-400 (Bruker, Germany) spectrometers using CDCl_3_ as the solvent and TMS as the internal standard. Melting points were measured using an uncorrected X-4B melting point apparatus (Shanghai Yidian Physical Optical Instrument Co., Ltd., China). High-resolution mass spectrometry (HRMS) analyses were performed on a Thermo Scientific Orbitrap LC-MS Q Exactive system (Thermo Fisher Scientific, USA).

**Synthesis of intermediates a-e and preparation of the target compound series F1-F37**

**Synthesis of intermediate a and b**

Intermediates **a** and **b** were synthesized according to previously reported procedures (Li *et al*., 2018).

**Synthesis of intermediate c**

Intermediate **b** (4.82 mmol) was dissolved in dichloromethane (20 mL) in a single-necked round-bottom flask. EDCI (5.78 mmol), HOBt (5.78 mmol), and triethylamine (7.22 mmol) were added sequentially. After stirring at room temperature for 30 minutes, 1-(tert-butoxycarbonyl)piperazine (4.82 mmol) was added. The reaction mixture was stirred at ambient temperature for an additional 6-8 hours, and progress was monitored by thin-layer chromatography (TLC). Upon completion, the reaction mixture was diluted with dichloromethane (50 mL) and extracted with water (3 × 30 mL). The organic layer was separated, washed twice with saturated brine, dried over anhydrous magnesium sulfate, filtered, and purified by column chromatography to yield intermediate **c** (Wang *et al*., 2014).

**Synthesis of intermediate d**

3-Methoxy-1-propanol or 2-methoxyethanol (3.08 mmol) was dissolved in dimethylformamide (DMF, 10 mL), and sodium hydride (6.16 mmol) was added under ice-bath conditions. After stirring for 30 minutes, the reaction mixture was allowed to warm to room temperature, followed by the addition of intermediate **c** (1.54 mmol). The mixture was stirred at room temperature for an additional 6 hours. Upon completion, the reaction mixture was slowly poured into ice water, and the product was extracted with ethyl acetate (50 mL). The combined organic layers were washed with saturated brine (3 × 30 mL), dried over anhydrous magnesium sulfate, filtered, and purified by column chromatography to afford intermediate **d** (Li *et al*., 2019).

**Synthesis of intermediate e**

Intermediate **d** (1.16 mmol) was dissolved in dichloromethane (15 mL), and trifluoroacetic acid (0.5 mL) was slowly added under an ice-water bath. After stirring for 1 hour, the reaction mixture was warmed to room temperature and stirred for an additional 2 hours. Upon completion, the solvent was removed under reduced pressure. The resulting residue was diluted with saturated aqueous sodium bicarbonate and extracted with ethyl acetate (3 × 30 mL). The combined organic layers were dried over anhydrous magnesium sulfate, filtered, concentrated, and purified by column chromatography to yield intermediate **e** (Su *et al*., 2019).

**Synthesis of target compounds F1-F37**

Intermediate **e** (0.96 mmol) and triethylamine (1.92 mmol) were added sequentially to dimethylformamide (DMF, 5 mL). Under an ice-water bath, carbon disulfide (1.44 mmol) was added dropwise. After stirring for 30 minutes, halide salts of substituted chloroamines (1.92 mmol) were introduced. The reaction mixture was stirred at room temperature for an additional 6-8 hours. Upon completion, the mixture was poured into water and extracted with ethyl acetate (3 × 30 mL). The combined organic layers were dried over anhydrous magnesium sulfate, filtered, and concentrated under reduced pressure. The crude product was purified by column chromatography to yield the final compounds **F1-F37**.

**Protein purification**

The prokaryotic expression vectors (pET-32a-N^WT^, pET-32a-N^R94A&K192A&G228A^, pET-21a-sfGFP-N^WT^, pET-21a-sfGFP-N^R94A&K192A&G228A^, and pET-21a-sfGFP-NbPGK) were transformed into *Escherichia coli* BL21 (DE3) cells for protein expression. Bacterial cultures were induced with 0.8 mL isopropyl-β-D-1-thiogalactopyranoside (IPTG) at 28°C for 16 h. After induction, the cells were harvested and resuspended in lysis buffer, followed by ultrasonic disruption. The lysis buffer consisted of 37.5 mL PBS, 8.69 g NaCl, 0.36 mL β-mercaptoethanol, and 5 mL glycerol, with ddH₂O added to a final volume of 500 mL (pH 7.4). Cell lysates were clarified by centrifugation at 12,000 rpm for 30 minutes. The supernatant was then purified by affinity chromatography at 4°C using a Trap high-performance column (GE Healthcare, USA).

**Agrobacterium infects** ***N. benthamiana***

The recombinant plasmids were transformed into *Agrobacterium* competent cell GV3101 (Tsingke) and cultured at 28 °C for 48 hours. Single colonies were selected and further cultured at 28 °C for 36-48 hours. cells were harvested by centrifugation at 6000 rpm and resuspended in agroinfiltration buffer (10 mM MgCl₂, 10 mM MES, PH 5.6, 100 µM acetosyringone), with the OD_600_ adjusted to 1.0. The final OD_600_ values of L_(+)opt_, M_(-)opt_, SR_(+)eGFP_ components in the infection buffer were 0.2, and that of VSRs was 0.1. After mixing, the bacterial suspension was incubated at room temperature in the dark for 3 hours, followed by syringe infiltration into *N. benthamiana* leaves at the 5-7 leaf stage. For the transient expression vector pCambia2300-N-YFP, cells were resuspended in the same agroinfiltration buffer and adjusted to a final OD_600_ of 0.5-0.6. The suspension was similarly incubated at room temperature in the dark for 3 hours before infiltration into *N. benthamiana* leaves at the 5-7 leaf stage. All infiltrated plants were maintained in an artificial climate chamber for subsequent analysis. The treatments for all mutant constructs were performed using the same protocol.

**RNA extraction and qRT-PCR**

Total RNA was extracted from samples using TRIzol Reagent (TaKaRa). The extracted RNA was reverse transcribed into cDNA using M-MLV Reverse Transcriptase (RR047A, TaKaRa). The cDNAs were diluted appropriately and used as templates for quantitative PCR (qPCR) with TB Green® Premix Ex Taq™ II (Tli RNase H Plus, TaKaRa). Gene expression levels were quantified using Bio-Rad CFX Manager software. The Actin gene was used as an internal reference to normalize target gene expression. Relative expression was calculated using the ΔCt method (ΔCt = Ct target gene − Ct Actin). Each sample included three biological replicates and three technical replicates to ensure result reliability. Primer sequences used in this study are provided in Table S4 and Table S5.

**Protein extracts and immunoblot assays**

Healthy leaves, agroinfiltrated leaf patches, or systemically TSWV-infected *N. benthamiana* leaves were collected. Total protein was extracted from 1 g of leaf tissue using 1 mL of extraction buffer composed of 150 mM NaCl, 1 mM EDTA, 10 mM dithiothreitol, 25 mM Tris-HCl (pH 7.5), 2% (w/v) polyvinylpolypyrrolidone, 0.5% (v/v) Triton X-100, 10% (v/v) glycerol, and 1 × protease inhibitor cocktail. Protein samples were separated by SDS–PAGE and transferred onto PVDF membranes. Membranes were blocked with 5% (w/v) skim milk and incubated with a rabbit polyclonal anti-N antibody (1:1000; Youlong Biosciences Co., Ltd., Shanghai, China). RuBisCO, stained with Ponceau S, was used as a loading control. Protein signals were detected using a ChemiDoc MP Imaging System (Bio-Rad, Hercules, USA).

**Data Analysis**

All experimental data were processed using Microsoft Office Excel 2019 or GraphPad Prism 8.0 (GraphPad Software, San Diego, CA, USA). Statistical significance between groups was assessed using GraphPad Prism 8.0. P-values were calculated based on three independent replicates using either a two-tailed t-test or a one-tailed paired t-test. Data are presented as mean ± standard deviation (mean ± SD). Four levels of significance were applied for all tests: *(p < 0.05), **(p < 0.01), ***(p < 0.001), and ****(p < 0.0001).

**Coarse-grained molecular dynamics (CGMD) simulations**

The protein used in the simulation, TSWV N (PDB ID: 5IP1), and ATP were parameterized using the Martini 3.0 coarse-grained force field obtained via CHARMM-GUI (Jo *et al.,* 2008; Brooks *et al.,* 2009). Coarse-grained mapping: ATP was represented by three beads corresponding to (1) the adenine group, (2) the ribose group, and (3) the triphosphate group. The triphosphate bead was assigned a total charge of -4. A total of twenty N protein (5IP1) molecules were placed in a 30 × 30 × 30 nm^3^ water box. ATP molecules were randomly added at molar ratios of 1, 5, 10, 50, and 500 relative to N, and counterions were introduced to maintain overall charge neutrality. All simulations were performed using the GROMACS 2025.2 software package. Periodic boundary conditions (PBCs) were applied in all simulations to conserve the number of particles. After system construction, energy minimization was carried out for 50000 steps. The non-bonded interaction cutoff was set to 11 Å, and long-range electrostatic interactions were computed using the reaction-field method. Temperature was maintained at 277.15 K using the V-rescale thermostat with a coupling constant of 0.2 ps, while pressure was maintained at 1 bar using the C-rescale barostat. The LINCS algorithm was employed to constrain bond lengths involving hydrogen atoms. The time step was set to 5 fs, and the final production run was performed for 200 ns for each system.

**Phytotoxicity assay and antiviral activity evaluation of compound F10 in tomato plants**

Tomato plants (*Solanum lycopersicum*, M82) were used to assess the phytotoxicity and antiviral efficacy of the small-molecule compound **F10**. For the phytotoxicity assay, tomato leaves were uniformly sprayed with **F10** solutions at three concentrations (500, 1000, and 1500 µg/mL), while water-treated plants containing an equivalent amount of DMSO served as controls. All plants were maintained in an artificial climate chamber. Phenotypic observations were conducted five days after treatment to evaluate potential morphological or growth abnormalities. For the antiviral assay against TSWV, **F10** was applied at a concentration of 500 µg/mL. Ningnanmycin, a healthy control, and a virus-infected untreated group (wild-type) were included as references. Following mechanical inoculation with TSWV, the compound was evenly sprayed onto tomato leaves six hours post-inoculation. The treated plants were subsequently maintained in an artificial climate chamber for 21 days. Phenotypic changes were recorded, and leaf samples were collected for further molecular and biochemical analyses. Each treatment group consisted of three biological replicates.

**4. Characterizations of products**

 *2-(dimethylamino)ethyl 4-(2-(2-methoxyethoxy)-6-methylquinoline-4-carbonyl)piperazine-1-carbodithioate* (**F1**). White solid; m.p. 95.3-97.1 ℃; yield 71.1 %; ^1^H NMR (500 MHz, CDCl_3_) *δ* 7.72 (d, *J* = 8.5 Hz, 1H, Ar-H), 7.46 (dd, *J* = 8.6, 1.8 Hz, 1H, Ar-H), 7.35(s, 1H, Ar-H), 6.84(s, 1H, Ar-H), 4.68 – 4.57 (m, 2H, -CH_2_-), 4.30 (d, *J* = 91.8 Hz, 2H, -CH_2_-), 4.12 – 3.60 (m, 6H, -3CH_2_-), 3.46 – 3.40 (m, 5H, -CH_2_-, -OCH_3_), 3.30 (d, *J* = 15.5 Hz, 2H, -CH_2_-), 2.63 – 2.55 (m, 2H, -CH_2_-), 2.43(s, 3H, -CH_3_), 2.25(s, 6H, -2CH_3_). ^13^C NMR (125 MHz, CDCl_3_) δ 198.44, 167.28, 160.73, 145.25, 143.56, 135.15, 132.64, 127.81, 123.23, 121.10, 110.33, 70.97, 65.36, 59.26, 57.73, 46.18, 45.42, 41.27, 35.64, 21.64. HRMS (ESI): Calculated for C_23_H_33_O_3_N_4_S_2_ [M+H]^+^:477.19886, found: 477.19809.

*3-(dimethylamino)propyl 4-(2-(2-methoxyethoxy)-6-methylquinoline-4-carbonyl)piperazine-1-carbodithioate* (**F2**). White solid; m.p. 97.3-98.9 ℃; yield 62.64 %; ^1^H NMR (500 MHz, CDCl_3_) *δ* 7.73 (d, *J* = 8.5 Hz, 1H, Ar-H), 7.47 (dd, *J* = 8.6, 1.8 Hz, 1H, Ar-H), 7.36(s, 1H, Ar-H), 6.85(s, 1H, Ar-H), 4.69 – 4.59 (m, 2H, -CH_2_-), 4.28 (d, *J* = 92.2 Hz, 2H, -CH_2_-), 4.10 – 3.63 (m, 6H, -3CH_2_-), 3.44(s, 3H, -CH_3_), 3.37 – 3.27 (m, 4H, -2CH_2_-), 2.44(s, 3H, -CH_3_), 2.35 (t, *J* = 7.1 Hz, 2H, -CH_2_-), 2.20(s, 6H, -2CH_3_), 1.85 (p, *J* = 7.2 Hz, 2H, -CH_2_-).^13^C NMR (125 MHz, CDCl_3_) δ 198.48, 167.30, 160.74, 145.26, 143.60, 135.17, 132.65, 127.81, 123.24, 121.11, 110.33, 70.99, 65.37, 59.28, 58.59, 46.20, 45.58, 41.26, 35.21, 26.84, 21.65. HRMS (ESI): Calculated for C_24_H_35_O_3_N_4_S_2_ [M+H]^+^:491.21451, found: 491.21368.

*3-(diethylamino)propyl 4-(2-(2-methoxyethoxy)-6-methylquinoline-4-carbonyl)piperazine-1-carbodithioate* (**F3**). White solid; m.p. 97.7-99.2 ℃; yield 58.46 %; ^1^H NMR (500 MHz, CDCl_3_) *δ* 7.74 (d, *J* = 8.5 Hz, 1H, Ar-H), 7.49 – 7.45 (m, 1H, Ar-H), 7.37(s, 1H, Ar-H), 6.85(s, 1H, Ar-H), 4.69 – 4.58 (m, 2H, -CH_2_-), 4.30 (d, *J* = 77.5 Hz, 2H, -CH_2_-), 4.10 – 3.65 (m, 6H, -3CH_2_-), 3.44(s, 3H, -CH_3_), 3.38 – 3.23 (m, 4H, -2CH_2_-), 2.53 – 2.47 (m, 6H, -3CH_2_-), 2.45(s, 3H, -CH_3_), 1.82 (d, *J* = 7.4 Hz, 4H, -2CH_2_-), 0.99 (t, *J* = 7.1 Hz, 6H, -2CH_3_). ^13^C NMR (125 MHz, CDCl_3_) δ 198.64, 167.31, 160.75, 145.26, 143.60, 135.18, 132.65, 127.81, 123.24, 121.11, 110.33, 70.99, 65.37, 59.28, 51.69, 46.90, 46.21, 41.27, 35.50, 26.11, 21.65, 11.82. HRMS (ESI): Calculated for C_26_H_39_O_3_N_4_S_2_ [M+H]^+^:519.24581, found: 519.24512.

*2-(dimethylamino)ethyl 4-(2-(3-methoxypropoxy)-6-methylquinoline-4-carbonyl)piperazine-1-carbodithioate* (**F4**). White solid; m.p. 68.5-69.3 ℃; yield 80.55 %; ^1^H NMR (500 MHz, CDCl_3_) *δ* 7.75 (d, *J* = 8.5 Hz, 1H, Ar-H), 7.47 (dd, *J* = 8.6, 1.8 Hz, 1H, Ar-H), 7.36(s, 1H, Ar-H), 6.78(s, 1H, Ar-H), 4.53 (d, *J* = 25.2 Hz, 2H, -CH_2_-), 4.32(s, 2H, -CH_2_-), 4.17 – 3.69 (m, 4H, -2CH_2_-), 3.61 – 3.52 (m, 2H, -CH_2_-), 3.46 (t, *J* = 6.6 Hz, 2H, -CH_2_-), 3.38 – 3.26 (m, 2H, -CH_2_-, -CH_3_), 2.62 (t, *J* = 6.7 Hz, 2H, -CH_2_-), 2.45(s, 3H, -CH_3_), 2.27(s, 6H, -2CH_3_), 2.09 (p, *J* = 6.4 Hz, 2H, -CH_2_-). ^13^C NMR (125 MHz, CDCl_3_) *δ* 198.51, 167.39, 161.00, 145.45, 143.51, 135.01, 132.62, 127.84, 123.17, 120.96, 110.26, 69.55, 63.38, 58.84, 57.74, 46.21, 45.41, 41.28, 35.62, 29.30, 21.63. HRMS (ESI): Calculated for C_24_H_35_O_3_N_4_S_2_ [M+H]^+^:491.21451, found: 49121368.

*2-(diethylamino)ethyl 4-(2-(3-methoxypropoxy)-6-methylquinoline-4-carbonyl)piperazine-1-carbodithioate* (**F5**). Colorless oil; yield 63.47 %; ^1^H NMR (500 MHz, CDCl_3_) *δ* 7.74 (d, *J* = 8.5 Hz, 1H, Ar-H), 7.47 (dd, *J* = 8.6, 1.8 Hz, 1H, Ar-H), 7.36(s, 1H, Ar-H), 6.78(s, 1H, Ar-H), 4.52 (d, *J* = 28.3 Hz, 2H, -CH_2_-), 4.41 – 3.66 (m, 6H, -3CH_2_-), 3.55 (dd, *J* = 13.1, 7.3 Hz, 2H, -CH_2_-), 3.44 – 3.38 (m, 2H, -CH_2_-), 3.37-3.25(m, 2H, -CH_2_-, -CH_3_), 2.79 – 2.70 (m, 2H, -CH_2_-), 2.58 (q, *J* = 7.9, 7.1 Hz, 4H, -2CH_2_-), 2.44(s, 2H, -CH_2_-), 2.08 (p, *J* = 6.4 Hz, 2H, -CH_2_-), 1.02 (t, *J* = 7.2 Hz, 6H, -2CH_3_). ^13^C NMR (125 MHz, CDCl_3_) δ 198.64, 167.37, 161.00, 145.44, 143.53, 135.00, 132.59 (d, *J* = 4.6 Hz), 127.83, 123.17, 120.96, 110.26, 69.55, 63.37, 58.84, 51.22, 47.03, 46.22, 41.28, 35.03, 29.30, 21.63, 11.94. HRMS (ESI): Calculated for C_26_H_39_O_3_N_4_S_2_ [M+H]^+^:519.24581, found: 519.24506.

*3-(dimethylamino)propyl 4-(2-(3-methoxypropoxy)-6-methylquinoline-4-carbonyl)piperazine-1-carbodithioate* (**F6**). Colorless oil; yield 49.88%; ^1^H NMR (500 MHz, CDCl_3_) δ 7.74 (d, *J* = 8.5 Hz, 1H, Ar-H), 7.47 (dd, *J* = 8.6, 1.8 Hz, 1H, Ar-H), 7.37(s, 1H, Ar-H), 6.78(s, 1H, Ar-H), 4.53 (d, *J* = 25.4 Hz, 2H, -CH_2_-), 4.39 – 3.77 (m, 6H, -3CH_2_-), 3.56 (q, *J* = 5.8 Hz, 2H, -CH_2_-), 3.37 – 3.29 (m, 7H, -2CH_2_-, -CH_3_), 2.45(s, 3H, -CH_3_), 2.35 (t, *J* = 7.1 Hz, 2H, -CH_2_-), 2.21(s, 6H, -2CH_3_), 2.08 (p, *J* = 6.4 Hz, 2H, -CH_2_-), 1.86 (p, *J* = 7.2 Hz, 2H, -CH_2_-). ^13^C NMR (125 MHz, CDCl_3_) δ 198.53, 167.38, 161.00, 145.45, 143.54, 135.01, 132.61, 127.84, 123.18, 120.97, 110.26, 69.55, 63.38, 58.84, 58.60, 46.22, 45.57, 41.26, 35.23, 29.31, 26.86, 21.63. HRMS (ESI): Calculated for C_25_H_37_O_3_N_4_S_2_ [M+H]^+^:505.23016, found: 505.22931.

*3-(diethylamino)propyl 4-(2-(3-methoxypropoxy)-6-methylquinoline-4-carbonyl)piperazine-1-carbodithioate* (**F7**). Yellow oil; yield 66.89%; ^1^H NMR (500 MHz, CDCl_3_) *δ* 7.74 (d, *J* = 8.5 Hz, 1H, Ar-H), 7.47 (dd, *J* = 8.6, 1.7 Hz, 1H, Ar-H), 7.36(s, 1H, Ar-H), 6.78(s, 1H, Ar-H), 4.52 (d, *J* = 25.3 Hz, 2H, -CH_2_-), 4.17(s, 2H, -CH_2_-), 4.00 (t, *J* = 5.2 Hz, 4H, -2CH_2_-), 3.56 (q, *J* = 5.9 Hz, 2H, -CH_2_-), 3.35(s, 3H, -CH_3_), 3.30 (p, *J* = 9.0 Hz, 4H, -2CH_2_-), 2.53 – 2.44 (m, 6H, -3CH_2_-), 2.45(s, 3H, -CH_3_), 2.08 (p, *J* = 6.4 Hz, 2H, -CH_2_-), 1.82 (d, *J* = 7.1 Hz, 2H, -CH_2_-), 0.99 (t, *J* = 7.1 Hz, 6H, -2CH_3_). ^13^C NMR (125 MHz, CDCl_3_) *δ* 198.67, 167.38, 161.00, 145.44, 143.55, 135.01, 132.61, 127.83, 123.17, 120.96, 110.26, 69.55, 63.38, 58.84, 51.71, 46.91, 46.23, 41.26, 35.49, 29.30, 26.14, 21.63, 11.81. HRMS (ESI): Calculated for C_27_H_41_O_3_N_4_S_2_ [M+H]^+^:533.26146, found: 533.26062.

*2-(dimethylamino)ethyl 4-(6-fluoro-2-(2-methoxyethoxy)quinoline-4-carbonyl)piperazine-1-carbodithioate* (**F8**). White solid; m.p. 63.4-64.7 ℃; yield 70.28 %; ^1^H NMR (500 MHz, CDCl_3_) δ 7.84 (dd, *J* = 9.1, 5.2 Hz, 1H, Ar-H), 7.41 (td, *J* = 8.8, 2.7 Hz, 1H, Ar-H), 7.27 (dd, *J* = 9.1, 2.7 Hz, 1H, Ar-H), 6.92(s, 1H, Ar-H), 4.62(s, 2H, -CH_2_-), 4.47 – 3.88 (m, 6H, -3CH_2_-), 3.82 – 3.75 (m, 2H, -CH_2_-), 3.51 – 3.43 (m, 5H, -CH_2_-, -OCH_3_), 3.32(s, 2H, -CH_2_-), 2.63 (t, *J* = 6.6 Hz, 2H, -CH_2_-), 2.28(s, 6H, -2CH_3_). ^13^C NMR (125 MHz, CDCl_3_) δ 198.53, 166.58, 160.83, 160.70, 158.74, 143.83, 143.57, 143.53, 130.28, 130.21, 121.61 (d, *J* = 9.6 Hz), 120.30, 120.10, 111.47, 108.40, 108.21, 70.88, 65.56, 59.27, 57.71, 46.22, 45.37, 41.37, 35.56. HRMS (ESI): Calculated for C_22_H_30_O_3_N_4_FS_2_ [M+H]^+^:481.17379, found: 481.17316.

*2-(diethylamino)ethyl 4-(6-fluoro-2-(2-methoxyethoxy)quinoline-4-carbonyl)piperazine-1-carbodithioate* (**F9**). White solid; m.p. 53.3-54.8 ℃; yield 77.21 %; ^1^H NMR (600 MHz, CDCl_3_) *δ* 7.84 (dd, *J* = 9.2, 5.2 Hz, 1H, Ar-H), 7.44 – 7.39 (m, 1H, Ar-H), 7.28 (dd, *J* = 9.0, 2.6 Hz, 1H, Ar-H), 6.92(s, 1H, Ar-H), 4.63(s, 2H, -CH_2_-), 4.50 – 3.86 (m, 6H, -3CH_2_-), 3.79 (t, *J* = 4.3 Hz, 2H, -CH_2_-), 3.47 – 3.41 (m, 5H, -CH_2_-, -OCH_3_), 3.33(s, 2H, -CH_2_-), 2.76 (t, *J* = 7.0 Hz, 2H, -CH_2_-), 2.60 (q, *J* = 6.8 Hz, 4H, -2CH_2_-), 1.04 (t, *J* = 7.1 Hz, 6H, -2CH_3_). ^13^C NMR (150 MHz, CDCl_3_) *δ* 198.70, 166.58, 160.83, 160.70, 158.74, 143.82, 143.59, 143.55, 130.27, 130.20, 121.65, 121.57, 120.30, 120.10, 111.47, 108.40, 108.22, 70.88, 65.55, 59.26, 51.21, 47.03, 46.23, 41.37, 35.02, 11.91. HRMS (ESI): Calculated for C_24_H_34_O_3_N_4_FS_2_ [M+H]^+^:509.20509, found: 509.20438.

*3-(dimethylamino)propyl 4-(6-fluoro-2-(2-methoxyethoxy)quinoline-4-carbonyl)piperazine-1-carbodithioate* (**F10**). White solid; m.p. 62.1-63.9 ℃; yield 77.39%; ^1^H NMR (400 MHz, CDCl_3_) *δ* 7.86 (dd, *J* = 9.2, 5.2 Hz, 1H, Ar-H), 7.43 (td, *J* = 8.7, 2.8 Hz, 1H, Ar-H), 7.30 (dd, *J* = 9.1, 2.7 Hz, 1H, Ar-H), 6.94 (s, 1H, Ar-H), 4.64 (s, 2H, -CH_2_-), 4.52 – 3.89 (m, 6H, -3CH_2_-), 3.84 – 3.73 (m, 2H, -CH_2_-), 3.47 (s, 3H, -CH_3_), 3.35 (t, *J* = 7.3 Hz, 4H, -2CH_2_-), 2.38 (t, *J* = 7.2 Hz, 2H, -CH_2_-), 2.23 (s, 6H, -2CH_3_), 1.88 (p, *J* = 7.2 Hz, 2H, -CH_2_-). ^13^C NMR (100 MHz, CDCl_3_) *δ* 198.55, 166.51, 160.88, 160.76, 158.43, 143.76, 143.50, 130.20, 130.11, 121.59, 121.50, 120.23, 119.98, 111.39, 108.35, 108.12, 70.81, 65.47, 59.17, 58.51, 46.15, 45.46, 41.29, 35.18, 26.74. HRMS (ESI): Calculated for C_23_H_32_O_3_N_4_FS_2_ [M+H]^+^:495.18944, found: 495.18860.

*3-(diethylamino)propyl 4-(6-fluoro-2-(2-methoxyethoxy)quinoline-4-carbonyl)piperazine-1-carbodithioate* (**F11**). Colorless oil; yield 49.35%; ^1^H NMR (500 MHz, CDCl_3_) *δ* 7.83 (dd, *J* = 9.2, 5.2 Hz, 1H, Ar-H), 7.41 (ddd, *J* = 9.1, 8.2, 2.8 Hz, 1H, Ar-H), 7.27 (dd, *J* = 9.0, 2.8 Hz, 1H, Ar-H), 6.92(s, 1H, Ar-H), 4.61(s, 2H, -CH_2_-), 4.18(s, 2H, -CH_2_-), 3.98 (t, *J* = 5.2 Hz, 2H, -CH_2_-), 3.78 (t, *J* = 4.4 Hz, 2H, -CH_2_-), 3.44(s, 3H, -CH_3_), 3.35 – 3.27 (m, 4H, -2CH_2_-), 2.55 – 2.44 (m, 6H, -3CH_2_-), 1.84 (p, *J* = 7.3 Hz, 2H, -CH_2_-), 1.00 (t, *J* = 7.1 Hz, 6H, -2CH_3_), 0.83 (t, *J* = 6.8 Hz, 2H, -CH_2_-). ^13^C NMR (125 MHz, CDCl_3_) *δ* 198.73, 166.57, 160.76 (d, *J* = 17.8 Hz), 158.74, 143.82, 143.59 (d, *J* = 5.0 Hz), 130.23 (d, *J* = 8.6 Hz), 121.61 (d, *J* = 9.3 Hz), 120.29, 120.09, 111.46, 108.40, 108.22, 70.88, 65.55, 59.26, 51.66, 46.91, 46.23, 41.35, 35.47, 26.07, 11.78. HRMS (ESI): Calculated for C_25_H_36_O_3_N_4_FS_2_ [M+H]^+^:523.22074, found: 523.21997.

*2-(dimethylamino)ethyl 4-(6-fluoro-2-(3-methoxypropoxy)quinoline-4-carbonyl)piperazine-1-carbodithioate* (**F12**). White solid; m.p. 61.6-62.7 ℃; yield 85.43%; ^1^H NMR (400 MHz, DMSO-*d*_6_) δ 7.88 (dd, *J* = 9.2, 5.4 Hz, 1H, Ar-H), 7.63 (td, *J* = 8.8, 2.9 Hz, 1H, Ar-H), 7.48 (dd, *J* = 9.5, 2.8 Hz, 1H, Ar-H), 7.13 (s, 1H, Ar-H), 4.47 (t, *J* = 6.5 Hz, 2H, -CH_2_-), 4.16 (s, 2H, -CH_2_-), 3.87 (t, *J* = 5.1 Hz, 2H, -CH_2_-), 3.51 (t, *J* = 6.3 Hz, 2H, -CH_2_-), 3.40 – 3.36 (m, 2H, -CH_2_-), 3.33 (s, 4H, -2CH_2_-), 3.27 (s, 3H, -CH_3_), 2.54 – 2.51 (m, 2H, -CH_2_-), 2.16 (s, 6H, -2CH_3_), 2.03 (p, *J* = 6.4 Hz, 2H, -CH_2_-).^13^C NMR (100 MHz, CDCl_3_) δ 198.59, 166.66, 161.07, 160.63, 158.67, 144.00, 143.50, 143.46, 130.28, 130.21, 121.46, 121.39, 120.25, 120.05, 111.42, 108.32, 108.14, 69.46, 63.60, 58.85, 57.73, 46.24, 45.40, 41.37, 35.65, 29.24. HRMS (ESI): Calculated for C_23_H_32_O_3_N_4_FS_2_ [M+H]^+^:495.18944, found: 495.18866.

*2-(diethylamino)ethyl 4-(6-fluoro-2-(3-methoxypropoxy)quinoline-4-carbonyl)piperazine-1-carbodithioate* (**F13**). White solid; m.p. 127.3-128.5 ℃; yield 64.71%; ^1^H NMR (600 MHz, CDCl_3_) *δ* 7.84 (dd, *J* = 9.0, 5.2 Hz, 1H, Ar-H), 7.44 – 7.37 (m, 1H, Ar-H), 7.27 – 7.25 (m, 1H, Ar-H), 6.85(s, 1H, Ar-H), 4.53(s, 2H, -CH_2_-), 4.32 – 3.67 (m, 8H, -4CH_2_-), 3.55 (t, *J* = 6.0 Hz, 2H, -CH_2_-), 3.35(s, 2H, -CH_2_-, -OCH_3_), 3.15 (d, *J* = 34.0 Hz, 6H, -3CH_2_-), 2.08 (p, *J* = 6.2 Hz, 2H, -CH_2_-), 1.39 (t, *J* = 6.9 Hz, 6H, -2CH_3_). ^13^C NMR (150 MHz, CDCl_3_) *δ* 196.18, 166.64, 161.03, 160.60, 158.64, 144.00, 143.30, 143.27, 121.38, 121.30, 120.25, 120.05, 111.44, 108.22, 108.04, 69.42, 63.62, 58.82, 50.72, 46.69, 46.08, 41.23, 29.20, 9.02. HRMS (ESI): Calculated for C_25_H_36_O_3_N_4_FS_2_ [M+H]^+^:523.22704, found: 523.21997.

*3-(dimethylamino)propyl 4-(6-fluoro-2-(3-methoxypropoxy)quinoline-4-carbonyl)piperazine-1-carbodithioate* (**F14**). White solid; m.p. 52.7-54.3 ℃; yield 74.61%; ^1^H NMR (500 MHz, CDCl_3_) *δ* 7.82 (dd, *J* = 9.2, 5.3 Hz, 1H, Ar-H), 7.39 (td, *J* = 8.6, 2.8 Hz, 1H, Ar-H), 7.27 – 7.24 (m, 1H, Ar-H), 6.84(s, 1H, Ar-H), 4.51(s, 2H, -CH_2_-), 4.26(s, 2H, -CH_2_-), 4.04 (d, *J* = 62.4 Hz, 4H, -2CH_2_-), 3.54 (t, *J* = 6.2 Hz, 2H, -CH_2_-), 3.35 – 3.28 (m, 7H, -2CH_2_-, -CH_3_), 2.45 (t, *J* = 7.3 Hz, 2H, -CH_2_-), 2.28(s, 2H, -2CH_3_), 2.09 – 2.03 (m, 2H, -CH_2_-), 1.90 (p, *J* = 7.3 Hz, 2H, -CH_2_-). ^13^C NMR (125 MHz, CDCl_3_) *δ* 198.34, 166.65, 161.06, 160.61, 158.65, 143.99, 143.50, 143.47, 130.27, 130.20, 121.45, 121.37, 120.24, 120.04, 111.41, 108.31, 108.12, 69.45, 63.59, 58.83, 58.26, 46.22, 45.15, 41.33, 34.90, 29.22, 26.41. HRMS (ESI): Calculated for C_24_H_34_O_3_N_4_FS_2_ [M+H]^+^:509.20509, found: 509.20432.

**3-(diethylamino)propyl 4-(6-fluoro-2-(3-methoxypropoxy)quinoline-4-carbonyl)piperazine-1-carbodithioate (**F15**). White solid; m.p. 55.3-56.7 ℃; yield 61.09%; ^1^H NMR (400 MHz, CDCl_3_) *δ* 7.86 (dd, *J* = 9.2, 5.2 Hz, 1H, Ar-H), 7.42 (td, *J* = 8.7, 8.3, 2.8 Hz, 1H, Ar-H), 7.29 (dd, *J* = 8.7, 2.4 Hz, 1H, Ar-H), 6.87 (s, 1H, Ar-H), 4.55 (s, 2H, -CH_2_-), 4.42 – 3.92 (m, 6H, -3CH_2_-), 3.57 (t, *J* = 6.3 Hz, 2H, -CH_2_-), 3.41 – 3.30 (m, 7H, -CH_3,_ -2CH_2_-), 2.53 (q, *J* = 7.2 Hz, 6H, -3CH_2_-), 2.10 (p, *J* = 6.4 Hz, 2H, -CH_2_-), 1.86 (p, *J* = 7.3 Hz, 2H, -CH_2_-), 1.02 (t, *J* = 7.1 Hz, 6H, -2CH_3_). ^13^C NMR (100 MHz, CDCl_3_) *δ* 198.66, 166.56, 161.00, 160.98, 160.78, 158.33, 143.93, 143.48, 143.43, 130.19, 130.10, 121.41, 121.32, 120.14, 119.89, 111.32, 108.26, 108.03, 69.38, 63.52, 58.71, 51.61, 46.83, 46.15, 41.26, 35.39, 29.17, 26.06, 11.69. HRMS (ESI): Calculated for C_26_H_38_O_3_N_4_FS_2_ [M+H]^+^:537.23639, found: 537.23560.

*2-(dimethylamino)ethyl 4-(6-chloro-2-(2-methoxyethoxy)quinoline-4-carbonyl)piperazine-1-carbodithioate* (**F16**). Colorless oil; yield 55.82%; ^1^H NMR (500 MHz, CDCl_3_) *δ* 7.76 (d, *J* = 8.8 Hz, 1H, Ar-H), 7.59 (d, *J* = 2.1 Hz, 1H, Ar-H), 7.56 (dd, *J* = 8.9, 2.3 Hz, 1H, Ar-H), 6.89(s, 1H, Ar-H), 4.60(s, 2H, -CH_2_-), 4.08 (d, *J* = 113.9 Hz, 6H, -3CH_2_-), 3.77 (t, *J* = 4.3 Hz, 2H, -CH_2_-), 3.43 (t, *J* = 7.2 Hz, 5H, -CH_2_-, -CH_3_), 3.31(s, 2H, -CH_2_-), 2.60 (t, *J* = 6.7 Hz, 2H, -CH_2_-), 2.26(s, 6H, -2CH_3_). ^13^C NMR (125 MHz, CDCl_3_) δ 198.54, 166.39, 161.38, 145.41, 143.31, 131.30, 130.89, 129.60, 123.30, 121.92, 111.52, 70.82, 65.66, 59.25, 57.73, 46.23, 45.40, 41.38, 35.65. HRMS (ESI): Calculated for C_22_H_30_O_3_N_4_ClS_2_ [M+H]^+^:497.14424, found: 497.14352.

*2-(diethylamino)ethyl 4-(6-chloro-2-(2-methoxyethoxy)quinoline-4-carbonyl)piperazine-1-carbodithioate* (**F17**). Colorless oil; yield 69.33%; ^1^H NMR (500 MHz, CDCl_3_) *δ* 7.76 (d, *J* = 8.8 Hz, 1H, Ar-H), 7.60 – 7.58 (m, 1H, Ar-H), 7.56 (dd, *J* = 8.9, 2.3 Hz, 1H, Ar-H), 6.89(s, 1H, Ar-H), 4.60(s, 2H, -CH_2_-), 4.49 – 3.84 (m, 6H, -3CH_2_-), 3.76 (t, *J* = 4.3 Hz, 2H, -CH_2_-), 3.43 – 3.36 (m, 5H, -CH_2_-, -CH_3_), 3.31(s, 2H, -CH_2_-), 2.72 (t, *J* = 7.2 Hz, 2H, -CH_2_-), 2.57 (q, *J* = 7.1 Hz, 4H, -2CH_2_-), 1.00 (t, *J* = 7.1 Hz, 6H, -2CH_3_). ^13^C NMR (125 MHz, CDCl_3_) δ 198.67, 166.38, 161.38, 145.41, 143.33, 131.29, 130.88, 129.60, 123.30, 121.92, 111.51, 70.82, 65.65, 59.24, 51.22, 47.03, 46.23, 41.37, 35.08, 11.95. HRMS (ESI): Calculated for C_24_H_34_O_3_N_4_ClS_2_ [M+H]^+^:525.17554, found: 525.17511.

*3-(dimethylamino)propyl 4-(6-chloro-2-(2-methoxyethoxy)quinoline-4-carbonyl)piperazine-1-carbodithioate* (**F18**). White solid; m.p. 85.1-86.4 ℃; yield 77.37%; ^1^H NMR (500 MHz, CDCl_3_) *δ* 7.78 (d, *J* = 8.9 Hz, 1H, Ar-H), 7.61 (d, *J* = 2.1 Hz, 1H, Ar-H), 7.58 (dd, *J* = 8.8, 2.3 Hz, 1H, Ar-H), 6.91(s, 1H, Ar-H), 4.62(s, 2H, -CH_2_-), 4.48 – 3.84 (m, 6H, -3CH_2_-), 3.79 (t, *J* = 4.2 Hz, 2H, -CH_2_-), 3.45(s, 3H, -CH_3_), 3.33 (t, *J* = 7.3 Hz, 4H, -2CH_2_-), 2.39 – 2.31 (m, 2H, -CH_2_-), 2.21(s, 6H, -2CH_3_), 1.86 (p, *J* = 7.2 Hz, 2H, -CH_2_-). ^13^C NMR (125 MHz, CDCl_3_) δ 198.61, 166.42, 161.40, 145.42, 143.35, 131.32, 130.93, 129.61, 123.31, 121.93, 111.53, 70.84, 65.68, 59.27, 58.59, 46.26, 45.56, 41.38, 35.25, 26.82. HRMS (ESI): Calculated for C_23_H_32_O_3_N_4_ClS_2_ [M+H]^+^:511.15989, found: 511.15939.

*3-(diethylamino)propyl 4-(6-chloro-2-(2-methoxyethoxy)quinoline-4-carbonyl)piperazine-1-carbodithioate* (**F19**). White solid; m.p. 83.4-84.6 ℃; yield 65.90%; ^1^H NMR (500 MHz, CDCl_3_) δ 7.78 (d, *J* = 8.8 Hz, 1H, Ar-H), 7.61 (d, *J* = 2.2 Hz, 1H, Ar-H), 7.58 (dd, *J* = 8.8, 2.3 Hz, 1H, Ar-H), 6.91(s, 1H, Ar-H), 4.62(s, 2H, -CH_2_-), 4.52 – 3.85 (m, 6H, -3CH_2_-), 3.79 (t, *J* = 4.3 Hz, 2H, -CH_2_-), 3.44(s, 3H, -CH_3_), 3.35 – 3.27 (m, 4H, -2CH_2_-), 2.51 (q, *J* = 7.2 Hz, 2H, -3CH_2_-), 1.84 (p, *J* = 7.4 Hz, 2H, -CH_2_-), 1.00 (t, *J* = 7.1 Hz, 6H, -2CH_3_). ^13^C NMR (125 MHz, CDCl_3_) *δ* 198.73, 166.42, 161.40, 145.42, 143.35, 131.32, 130.92, 129.61, 123.31, 121.93, 111.53, 70.84, 65.67, 59.27, 51.68, 46.92, 46.26, 41.38, 35.49, 29.80, 26.08, 11.78. HRMS (ESI): Calculated for C_25_H_36_O_3_N_4_ClS_2_ [M+H]^+^:539.19119, found: 539.19049.

*2-(dimethylamino)ethyl 4-(6-chloro-2-(3-methoxypropoxy)quinoline-4-carbonyl)piperazine-1-carbodithioate* (**F20**). Yellow oil; yield 51.28%; ^1^H NMR (400 MHz, CDCl_3_) *δ* 7.81 (d, *J* = 8.8 Hz, 1H, Ar-H), 7.64 – 7.57 (m, 2H, Ar-H), 6.86 (s, 1H, Ar-H), 4.55 (s, 2H, -CH_2_-), 4.24 (s, 2H, -CH_2_-), 4.15 – 3.78 (m, 4H, -2CH_2_-), 3.58 (t, *J* = 6.2 Hz, 2H, -CH_2_-), 3.48 (t, *J* = 6.7 Hz, 2H, -CH_2_-), 3.38 (s, 5H, -CH_2_-, -CH_3_), 2.64 (t, *J* = 6.7 Hz, 2H, -CH_2_-), 2.30 (s, 6H, -2CH_3_), 2.11 (p, *J* = 6.3 Hz, 2H, -CH_2_-). ^13^C NMR (100 MHz, CDCl_3_) *δ* 198.47, 166.41, 161.54, 145.51, 143.14, 131.20, 130.67, 129.55, 123.15, 121.68, 111.41, 69.35, 63.66, 58.78, 57.64, 46.19, 45.31, 41.31, 35.52, 29.14. HRMS (ESI): Calculated for C_23_H_32_O_3_N_4_ClS_2_ [M+H]^+^:511.15989, found: 511.15927.

*2-(diethylamino)ethyl 4-(6-chloro-2-(3-methoxypropoxy)quinoline-4-carbonyl)piperazine-1-carbodithioate* (**F21**). Yellow solid; m.p. 65.3-67.0 ℃; yield 80.44%; ^1^H NMR (400 MHz, CDCl_3_) *δ* 7.81 (d, *J* = 8.8 Hz, 1H, Ar-H), 7.63 – 7.57 (m, 2H, Ar-H), 6.86 (s, 1H, Ar-H), 4.56 (s, 2H, -CH_2_-), 4.49-3.84 (m, 6H, -3CH_2_-), 3.57 (t, *J* = 6.2 Hz, 2H, -CH_2_-), 3.46 – 3.40 (m, 2H, -CH_2_-), 3.40 – 3.32 (m, 5H, -CH_3,_ -CH_2_-), 2.82 – 2.72 (m, 2H, -CH_2_-), 2.61 (q, *J* = 7.1 Hz, 4H, -2CH_2_-), 2.11 (p, *J* = 6.3 Hz, 2H, -CH_2_-), 1.05 (t, *J* = 7.1 Hz, 6H, -2CH_3_). ^13^C NMR (100 MHz, CDCl_3_) *δ* 198.66, 166.41, 161.54, 145.52, 143.17, 131.19, 130.67, 129.55, 123.16, 121.69, 111.40, 69.35, 63.66, 58.77, 51.14, 46.96, 46.20, 41.31, 35.03, 29.15, 11.87. HRMS (ESI): Calculated for C_25_H_36_O_3_N_4_ClS_2_ [M+H]^+^:539.19119, found: 539.19031.

*3-(dimethylamino)propyl 4-(6-chloro-2-(3-methoxypropoxy)quinoline-4-carbonyl)piperazine-1-carbodithioate* (**F22**). White solid; m.p. 56.3-57.4 ℃; yield 74.85%; ^1^H NMR (400 MHz, CDCl_3_) *δ* 7.81 (d, *J* = 8.8 Hz, 1H, Ar-H), 7.65 – 7.57 (m, 2H, Ar-H), 6.86 (s, 1H, Ar-H), 4.55 (s, 2H, -CH_2_-), 4.29 (s, 2H, -CH_2_-), 4.16 – 3.83 (m, 4H, -2CH_2_-), 3.57 (t, *J* = 6.2 Hz, 2H, -CH_2_-), 3.36 (d, *J* = 11.6 Hz, 7H, -2CH_2_-, -CH_3_), 2.37 (t, *J* = 7.2 Hz, 2H, -CH_2_-), 2.23 (s, 6H, -2CH_3_), 2.11 (p, *J* = 6.3 Hz, 2H, -CH_2_-), 1.88 (p, *J* = 7.3 Hz, 2H, -CH_2_-). ^13^C NMR (100 MHz, CDCl_3_) *δ* 198.55, 166.42, 161.55, 145.52, 143.18, 131.20, 130.69, 129.55, 123.16, 121.69, 111.40, 69.35, 63.66, 58.78, 58.53, 46.21, 45.50, 41.30, 35.19, 29.15, 26.77. HRMS (ESI): Calculated for C_24_H_34_O_3_N_4_ClS_2_ [M+H]^+^:525.17554, found: 525.17493.

*3-(diethylamino)propyl 4-(6-chloro-2-(3-methoxypropoxy)quinoline-4-carbonyl)piperazine-1-carbodithioate* (**F23**). White solid; m.p. 64.7-66.1 ℃; yield 63.65 %; ^1^H NMR (400 MHz, CDCl_3_) *δ* 7.81 (d, *J* = 8.8 Hz, 1H, Ar-H), 7.63 (d, *J* = 2.1 Hz, 1H, Ar-H), 7.60 (dd, *J* = 8.8, 2.3 Hz, 1H, Ar-H), 6.86 (s, 1H, Ar-H), 4.56 (s, 2H, -CH_2_-), 4.25 (s, 2H, -CH_2_-), 4.02 (t, *J* = 5.1 Hz, 2H, -CH_2_-), 3.57 (t, *J* = 6.2 Hz, 2H, -CH_2_-), 3.42 – 3.28 (m, 7H, -CH_3_, -2CH_2_-), 2.52 (q, *J* = 7.2 Hz, 6H, -3CH_2_-), 2.11 (p, *J* = 6.3 Hz, 2H, -CH_2_-), 1.86 (p, *J* = 7.3 Hz, 2H, -CH_2_-), 1.02 (t, *J* = 7.1 Hz, 6H, -2CH_3_). ^13^C NMR (100 MHz, CDCl_3_) δ 198.66, 166.41, 161.54, 145.51, 143.19, 131.19, 130.67, 129.54, 123.16, 121.69, 111.40, 69.35, 63.66, 58.77, 51.62, 46.84, 46.20, 41.30, 35.44, 29.14, 26.05, 11.74. HRMS (ESI): Calculated for C_26_H_38_O_3_N_4_ClS_2_ [M+H]^+^:553.20684, found: 553.20630.

*2-(dimethylamino)ethyl 4-(7-chloro-2-(2-methoxyethoxy)quinoline-4-carbonyl)piperazine-1-carbodithioate* (**F24**). White oil; yield 45.67%; ^1^H NMR (400 MHz, CDCl_3_) *δ* 7.88 (d, *J* = 2.0 Hz, 1H, Ar-H), 7.58 (d, *J* = 8.7 Hz, 1H, Ar-H), 7.37 (dd, *J* = 8.7, 2.0 Hz, 1H, Ar-H), 6.89 (s, 1H, Ar-H), 4.64 (s, 2H, -CH_2_-), 4.34 (s, 2H, -CH_2_-), 4.14 – 3.91 (m, 4H, -2CH_2_-), 3.81 (t, *J* = 4.4 Hz, 2H, -CH_2_-), 3.50 – 3.43 (m, 5H, -CH_3_, -CH_2_-), 3.32 (s, 2H, -CH_2_-), 2.64 (t, *J* = 6.8 Hz, 2H, -CH_2_-), 2.29 (s, 6H, -2CH_3_). ^13^C NMR (100 MHz, CDCl_3_) *δ* 198.51, 166.49, 161.90, 147.52, 143.98, 136.44, 127.27, 126.01, 125.40, 119.57, 110.66, 70.74, 65.63, 59.15, 57.66, 46.10, 45.30, 41.27, 35.55. HRMS (ESI): Calculated for C_22_H_30_O_3_N_4_ClS_2_ [M+H]^+^:497.14424, found: 497.14377.

* 3-(dimethylamino)propyl 4-(7-chloro-2-(2-methoxyethoxy)quinoline-4-carbonyl)piperazine-1-carbodithioate* (**F25**). White oil; yield 60.21%; ^1^H NMR (400 MHz, CDCl_3_) *δ* 7.88 (d, *J* = 1.8 Hz, 1H, Ar-H), 7.58 (d, *J* = 8.7 Hz, 1H, Ar-H), 7.37 (dd, *J* = 8.7, 1.8 Hz, 1H, Ar-H), 6.89 (s, 1H, Ar-H), 4.64 (s, 2H, -CH_2_-), 4.29 (s, 2H, -CH_2_-), 4.15-3.87(m, 4H, -2CH_2_-), 3.81 (t, *J* = 4.4 Hz, 2H, -CH_2_-), 3.47 (s, 3H, -CH_3_), 3.34 (t, *J* = 7.3 Hz, 4H, -2CH_2_-), 2.38 (t, *J* = 7.2 Hz, 2H, -CH_2_-), 2.24 (s, 6H, -2CH_3_), 1.88 (p, *J* = 7.2 Hz, 2H, -CH_2_-). ^13^C NMR (100 MHz, CDCl_3_) *δ* 198.54, 166.50, 161.91, 147.52, 144.01, 136.45, 127.27, 126.02, 125.41, 119.57, 110.66, 70.74, 65.63, 59.16, 58.48, 46.12, 45.43, 41.26, 35.15, 26.71. HRMS (ESI): Calculated for C_23_H_32_O_3_N_4_ClS_2_ [M+H]^+^:511.15989, found: 511.15927.

 *3-(diethylamino)propyl 4-(7-chloro-2-(2-methoxyethoxy)quinoline-4-carbonyl)piperazine-1-carbodithioate* (**F26**). Yellow oil; yield 48.97%; ^1^H NMR (400 MHz, CDCl_3_) *δ* 7.88 (d, *J* = 2.0 Hz, 1H, Ar-H), 7.58 (d, *J* = 8.7 Hz, 1H, Ar-H), 7.37 (dd, *J* = 8.7, 2.0 Hz, 1H, Ar-H), 6.90 (s, 1H, Ar-H), 4.64 (s, 2H, -CH_2_-), 4.47 – 3.94 (m, 6H, -3CH_2_-), 3.81 (t, *J* = 4.4 Hz, 2H, -CH_2_-), 3.46 (s, 3H, -CH_3_), 3.32 (t, *J* = 7.2 Hz, 4H, -CH_2_-), 2.55 – 2.49 (m, 6H, -3CH_2_-), 1.86 (p, *J* = 7.3 Hz, 2H, -CH_2_-), 1.02 (t, *J* = 7.1 Hz, 6H, -2CH_3_). ^13^C NMR (100 MHz, CDCl_3_) *δ* 198.67, 166.47, 161.90, 147.51, 144.01, 136.42, 127.25, 125.99, 125.42, 119.57, 110.65, 70.73, 65.62, 59.14, 51.63, 46.85, 46.11, 41.26, 35.41, 26.08, 11.73. HRMS (ESI): Calculated for C_25_H_36_O_3_N_4_ClS_2_ [M+H]^+^:539.19119, found: 539.19055.

 *2-(dimethylamino)ethyl 4-(7-chloro-2-(3-methoxypropoxy)quinoline-4-carbonyl)piperazine-1-carbodithioate* (**F27**). White solid; m.p. 89.5-91.4 ℃; yield 67.59%; ^1^H NMR (400 MHz, CDCl_3_) *δ* 7.88 (d, *J* = 2.0 Hz, 1H, Ar-H), 7.57 (d, *J* = 8.7 Hz, 1H, Ar-H), 7.36 (dd, *J* = 8.7, 2.0 Hz, 1H, Ar-H), 6.82 (s, 1H, Ar-H), 4.56 (s, 2H, -CH_2_-), 4.40 – 3.88 (m, 6H, -3CH_2_-), 3.57 (t, *J* = 6.2 Hz, 2H, -CH_2_-), 3.47 (t, *J* = 6.7 Hz, 2H, -CH_2_-), 3.38 (s, 3H, -CH_3_), 3.33 (s, 2H, -CH_2_-), 2.63 (t, *J* = 6.7 Hz, 2H, -CH_2_-), 2.29 (s, 6H, -2CH_3_), 2.10 (p, *J* = 6.3 Hz, 2H, -CH_2_-). ^13^C NMR (100 MHz, CDCl_3_) *δ* 198.53, 166.57, 162.13, 147.70, 143.88, 136.40, 127.27, 125.87, 125.33, 119.41, 110.61, 69.34, 63.72, 58.75, 57.65, 46.12, 45.30, 41.27, 35.53, 29.13. HRMS (ESI): Calculated for C_23_H_32_O_3_N_4_ClS_2_ [M+H]^+^:511.15989, found: 511.15952.

* 2-(diethylamino)ethyl 4-(7-chloro-2-(3-methoxypropoxy)quinoline-4-carbonyl)piperazine-1-carbodithioate* (**F28**). White solid; m.p. 77.8-79.3 ℃; yield 54.43%; ^1^H NMR (500 MHz, CDCl_3_) *δ* 7.86 (d, *J* = 2.0 Hz, 1H, Ar-H), 7.55 (d, *J* = 8.7 Hz, 1H, Ar-H), 7.34 (dd, *J* = 8.7, 2.1 Hz, 1H, Ar-H), 6.80(s, 1H, Ar-H), 4.53(s, 2H, -CH_2_-), 4.08 (d, *J* = 98.3 Hz, 6H, -3CH_2_-), 3.55 (t, *J* = 5.5 Hz, 2H, -CH_2_-), 3.45 – 3.39 (m, 2H, -CH_2_-), 3.38 – 3.25 (m, 5H, -CH_2_-, -OCH_3_), 2.75 (t, *J* = 7.1 Hz, 2H, -CH_2_-), 2.60 (q, *J* = 7.0 Hz, 4H, -2CH_2_-), 2.08 (p, *J* = 6.3 Hz, 2H, -CH_2_-), 1.03 (t, *J* = 7.1 Hz, 6H, -2CH_3_). ^13^C NMR (125 MHz, CDCl_3_) δ 198.72, 166.66, 162.20, 147.78, 143.96, 136.49, 127.36, 125.96, 125.40, 119.49, 110.69, 69.41, 63.79, 58.86, 51.18, 47.02, 46.22, 41.35, 34.98, 29.20, 11.86. HRMS (ESI): Calculated for C_25_H_36_O_3_N_4_ClS_2_ [M+H]^+^:539.19119, found: 539.19049.

 *3-(dimethylamino)propyl 4-(7-chloro-2-(3-methoxypropoxy)quinoline-4-carbonyl)piperazine-1-carbodithioate* (**F29**). Yellow solid; m.p. 85.3-86.9 ℃; yield 77.54%; ^1^H NMR (600 MHz, CDCl_3_) *δ* 7.86(s, 1H, Ar-H), 7.55 (d, *J* = 8.7 Hz, 1H, Ar-H), 7.34 (d, *J* = 8.5 Hz, 1H, Ar-H), 6.80(s, 1H, Ar-H), 4.53(s, 2H, -CH_2_-), 4.30(s, 2H, -CH_2_-), 4.01 (d, *J* = 39.8 Hz, 4H, -2CH_2_-), 3.55 (t, *J* = 5.5 Hz, 2H, -CH_2_-), 3.35(s, 3H, -CH_3_), 3.33 – 3.30 (m, 4H, -2CH_2_-), 2.34 (t, *J* = 7.1 Hz, 2H, -CH_2_-), 2.20(s, 6H, -2CH_3_), 2.08 (p, *J* = 6.3 Hz, 2H, -CH_2_-), 1.85 (p, *J* = 7.1 Hz, 2H, -CH_2_-). ^13^C NMR (150 MHz, CDCl_3_) *δ* 198.65, 166.66, 162.21, 147.79, 144.00, 136.48, 127.35, 125.95, 125.41, 119.49, 110.69, 69.42, 63.79, 58.82, 58.58, 46.21, 45.53, 41.33, 35.24, 29.21, 26.82. HRMS (ESI): Calculated for C_24_H_34_O_3_N_4_ClS_2_ [M+H]^+^:525.17554, found: 525.17523.

* 3-(diethylamino)propyl 4-(7-chloro-2-(3-methoxypropoxy)quinoline-4-carbonyl)piperazine-1-carbodithioate* (**F30**). White solid; m.p. 61.9-62.5 ℃; yield 63.87%; ^1^H NMR (600 MHz, CDCl_3_) *δ* 7.86 (d, *J* = 2.1 Hz, 1H, Ar-H), 7.56 (d, *J* = 8.7 Hz, 1H, Ar-H), 7.34 (dd, *J* = 8.7, 2.1 Hz, 1H, Ar-H), 6.80(s, 1H, Ar-H), 4.53(s, 2H, -CH_2_-), 4.39 – 3.79 (m, 6H, -3CH_2_-), 3.55 (t, *J* = 6.1 Hz, 2H, -CH_2_-), 3.36(s, 3H, -CH_3_), 3.33 – 3.28 (m, 4H, -2CH_2_-), 2.50 (q, *J* = 7.2 Hz, 6H, -3CH_2_-), 2.11 – 2.04 (m, 2H, -CH_2_-), 1.84 (p, *J* = 7.3 Hz, 2H, -CH_2_-), 1.00 (t, *J* = 7.1 Hz, 6H, -2CH_3_). ^13^C NMR (150 MHz, CDCl_3_) *δ* 198.83, 166.67, 162.22, 147.80, 144.01, 136.49, 127.36, 125.95, 125.40, 119.50, 110.69, 69.42, 63.80, 58.82, 51.74, 46.95, 46.22, 41.34, 35.50, 29.22, 26.21, 11.82. HRMS (ESI): Calculated for C_26_H_38_O_3_N_4_ClS_2_ [M+H]^+^:553.20684, found: 553.20624.

* 2-(dimethylamino)ethyl 4-(2-(2-methoxyethoxy)quinoline-4-carbonyl)piperazine-1-carbodithioate* (**F31**). White oil; yield 64.27%; ^1^H NMR (400 MHz, CDCl_3_) *δ* 7.87 (d, *J* = 8.3 Hz, 1H, Ar-H), 7.70 – 7.62 (m, 2H, Ar-H), 7.42 (ddd, *J* = 8.2, 7.0, 1.1 Hz, 1H, Ar-H), 6.92 (s, 1H, Ar-H), 4.66 (s, 2H, -CH_2_-), 4.40 (s, 2H, -CH_2_-), 4.07 (d, *J* = 49.1 Hz, 4H, -CH_2_-), 3.82 (t, *J* = 3.7 Hz, 2H, -CH_2_-), 3.50 – 3.44 (m, 5H, -CH_2_-, -CH_3_), 3.38 – 3.25 (m, 2H, -CH_2_-), 2.64 (t, *J* = 6.7 Hz, 2H, -CH_2_-), 2.30 (s, 6H, -2CH_3_). ^13^C NMR (100 MHz, CDCl_3_) δ 198.42, 166.99, 161.16, 146.83, 144.15, 130.50, 128.07, 125.23, 124.13, 121.10, 110.50, 70.87, 65.40, 59.19, 57.67, 46.11, 45.31, 41.23, 35.51. HRMS (ESI): Calculated for C_22_H_31_O_3_N_4_S_2_ [M+H]^+^:463.18321, found: 463.18250.

* 3-(dimethylamino)propyl 4-(2-(2-methoxyethoxy)quinoline-4-carbonyl)piperazine-1-carbodithioate* (**F32**). White solid; m.p. 68.3-69.8 ℃; yield 53.71%; ^1^H NMR (400 MHz, CDCl_3_) *δ* 7.87 (d, *J* = 8.2 Hz, 1H, Ar-H), 7.66 (ddd, *J* = 8.3, 5.7, 4.3 Hz, 2H, Ar-H), 7.41 (ddd, *J* = 8.2, 7.0, 1.1 Hz, 1H, Ar-H), 6.92 (s, 1H, Ar-H), 4.66 (s, 2H, -CH_2_-), 4.40 (s, 2H, -CH_2_-), 4.08 (d, *J* = 53.4 Hz, 4H, -CH_2_-), 3.82 (s, 2H, -CH_2_-), 3.47 (s, 3H, -CH_2_-), 3.34 (t, *J* = 7.3 Hz, 4H, -2CH_2_-), 2.36 (t, *J* = 7.2 Hz, 2H, -CH_2_-), 2.22 (s, 6H, -2CH_3_), 1.87 (p, *J* = 7.2 Hz, 2H, -CH_2_-). ^13^C NMR (100 MHz, CDCl_3_) δ 198.42, 166.96, 161.15, 146.81, 144.18, 130.48, 128.05, 125.21, 124.14, 121.09, 110.48, 70.85, 65.39, 59.17, 58.50, 46.10, 45.49, 41.19, 35.13, 26.76. HRMS (ESI): Calculated for C_23_H_33_O_3_N_4_S_2_ [M+H]^+^:477.19886, found: 477.19806.

 *3-(diethylamino)propyl 4-(2-(2-methoxyethoxy)quinoline-4-carbonyl)piperazine-1-carbodithioate* (**F33**). Yellow solid; m.p. 63.5-64.8 ℃; yield 66.69%; ^1^H NMR (400 MHz, CDCl_3_) *δ* 7.87 (d, *J* = 8.3 Hz, 1H, Ar-H), 7.66 (dd, *J* = 12.3, 7.6 Hz, 2H, Ar-H), 7.42 (t, *J* = 7.5 Hz, 1H, Ar-H), 6.92 (s, 1H, Ar-H), 4.66 (s, 2H, -CH_2_-), 4.39 (s, 2H, -CH_2_-), 4.08 (d, *J* = 47.9 Hz, 4H, -2CH_2_-), 3.82 (s, 2H, -CH_2_-), 3.47 (s, 3H, -CH_3_), 3.32 (t, *J* = 7.2 Hz, 4H, -2CH_2_-), 2.52 (q, *J* = 7.2 Hz, 6H, -3CH_2_-), 1.91 – 1.82 (m, 2H, -CH_2_-), 1.02 (t, *J* = 7.1 Hz, 6H, -2CH_3_). ^13^C NMR (100 MHz, CDCl_3_) *δ* 198.62, 166.99, 161.17, 146.82, 144.19, 130.49, 128.06, 125.23, 124.14, 121.10, 110.49, 70.87, 65.40, 59.19, 51.65, 46.85, 46.13, 41.21, 35.43, 26.08, 11.77. HRMS (ESI): Calculated for C_25_H_37_O_3_N_4_S_2_ [M+H]^+^:505.23016, found: 505.22922.

* 2-(dimethylamino)ethyl 4-(2-(3-methoxypropoxy)quinoline-4-carbonyl)piperazine-1-carbodithioate* (**F34**). White solid; m.p. 108.7-110.3 ℃; yield 64.36%; ^1^H NMR (500 MHz, CDCl_3_) *δ* 7.85 (d, *J* = 8.2 Hz, 1H, Ar-H), 7.67 – 7.58 (m, 2H, Ar-H), 7.41 – 7.36 (m, 1H, Ar-H), 6.83(s, 1H, Ar-H), 4.63 – 4.18 (m, 4H, -2CH_2_-), 4.15 – 3.72 (m, 4H, -2CH_2_-), 3.56 (q, *J* = 6.0 Hz, 2H, -CH_2_-), 3.45 (t, *J* = 6.6 Hz, 2H, -CH_2_-), 3.36(s, 3H, -CH_3_), 3.34 – 3.28 (m, 2H, -CH_2_-), 2.61 (t, *J* = 6.8 Hz, 4H, -2CH_2_-), 2.27(s, 6H, -2CH_3_), 2.09 (p, *J* = 6.4 Hz, 2H, -CH_2_-). ^13^C NMR (125 MHz, CDCl_3_) *δ* 198.54, 161.48, 147.06, 144.13, 130.59, 128.15, 125.20, 124.14, 121.00, 110.52, 69.51, 63.50, 58.89, 57.72, 46.22, 45.46, 41.30, 35.67, 29.27. HRMS (ESI): Calculated for C_23_H_33_O_3_N_4_S_2_ [M+H]^+^:477.19886, found: 477.19815.

* 2-(diethylamino)ethyl 4-(2-(3-methoxypropoxy)quinoline-4-carbonyl)piperazine-1-carbodithioate* (**F35**). White oil; yield 58.73%; ^1^H NMR (400 MHz, CDCl_3_) *δ* 7.87 (d, *J* = 8.2 Hz, 1H, Ar-H), 7.70 – 7.60 (m, 2H, Ar-H), 7.44 – 7.37 (m, 1H, Ar-H), 6.85 (s, 1H, Ar-H), 4.66 – 4.23 (m, 2H, -CH_2_-), 4.17 – 3.78 (m, 4H, -2CH_2_-), 3.61 – 3.54 (m, 2H, -CH_2_-), 3.48 – 3.40 (m, 2H, -CH_2_-), 3.38 – 3.32 (m, 5H, -CH_2_-, -CH_3_), 2.76 (td, *J* = 8.1, 7.1, 3.3 Hz, 2H, -CH_2_-), 2.61 (q, *J* = 7.3 Hz, 4H, -2CH_2_-), 2.11 (p, *J* = 6.4 Hz, 2H, -CH_2_-), 1.04 (t, *J* = 6.9 Hz, 6H, -2CH_3_). ^13^C NMR (100 MHz, CDCl_3_) δ 198.60, 167.06, 161.40, 147.00, 144.09, 130.46, 128.08, 125.09, 124.07, 120.95, 110.43, 69.44, 63.43, 58.77, 51.15, 46.96, 46.14, 41.22, 34.99, 29.21, 11.88. HRMS (ESI): Calculated for C_25_H_37_O_3_N_4_S_2_ [M+H]^+^:505.23016, found: 505.22943.

* 3-(dimethylamino)propyl 4-(2-(3-methoxypropoxy)quinoline-4-carbonyl)piperazine-1-carbodithioate* (**F36**). Yellow solid; m.p. 69.5-71.3 ℃; yield 60.55%; ^1^H NMR (500 MHz, CDCl_3_) δ 7.84 (d, *J* = 8.3 Hz, 1H, Ar-H), 7.66 – 7.58 (m, 2H, Ar-H), 7.37 (ddd, *J* = 8.1, 7.0, 1.0 Hz, 1H, Ar-H), 6.82(s, 1H, Ar-H), 4.63 – 4.17 (m, 4H, -2CH_2_-), 4.15-3.61 (m, 4H, -2CH_2_-), 3.55 (q, *J* = 5.9 Hz, 2H, -CH_2_-), 3.34(s, 3H, -CH_3_), 3.32 – 3.27 (m, 4H, -2CH_2_-), 2.36 – 2.28 (m, 2H, -CH_2_-), 2.19(s, 6H, -2CH_3_), 2.08 (p, *J* = 6.4 Hz, 2H, -CH_2_-), 1.88 – 1.78 (m, 2H, -CH_2_-). ^13^C NMR (125 MHz, CDCl_3_) δ 198.50, 167.13, 161.47, 147.05, 144.16, 130.57, 128.14, 125.19, 124.15, 121.00, 110.50, 69.49, 63.49, 58.87, 58.58, 46.22, 45.59, 41.26, 35.19, 29.26, 26.84. HRMS (ESI): Calculated for C_24_H_35_O_3_N_4_S_2_ [M+H]^+^:491.21451, found: 491.21387.

* 3-(diethylamino)propyl 4-(2-(3-methoxypropoxy)quinoline-4-carbonyl)piperazine-1-carbodithioate* (**F37**). White oil; yield 64.10%; ^1^H NMR (400 MHz, CDCl_3_) *δ* 7.87 (d, *J* = 8.2 Hz, 1H, Ar-H), 7.70 – 7.61 (m, 2H, Ar-H), 7.44 – 7.38 (m, 1H, Ar-H), 6.85 (s, 1H, Ar-H), 4.57 (d, *J* = 17.0 Hz, 2H, -CH_2_-), 4.30 (s, 2H, -CH_2_-), 4.14 – 3.73 (m, 4H, -2CH_2_-), 3.59 (s, 2H, -CH_2_-), 3.41 – 3.26 (m, 7H, -2CH_2_-, -CH_3_), 2.52 (q, *J* = 7.2 Hz, 6H, -3CH_2_-), 2.12 (p, *J* = 6.4 Hz, 2H, -CH_2_-), 1.85 (p, *J* = 7.4 Hz, 2H, -CH_2_-), 1.02 (t, *J* = 7.1 Hz, 6H, -2CH_3_). ^13^C NMR (100 MHz, CDCl_3_) *δ* 198.63, 167.07, 161.41, 147.00, 144.11, 130.46, 128.08, 125.09, 124.07, 120.95, 110.43, 69.44, 63.43, 58.77, 51.64, 46.85, 46.15, 41.21, 35.42, 29.21, 26.09, 11.77. HRMS (ESI): Calculated for C_26_H_39_O_3_N_4_S_2_ [M+H]^+^:519.24581, found: 519.24530.

**5. ^1^H, ^13^C NMR and HRMS data**


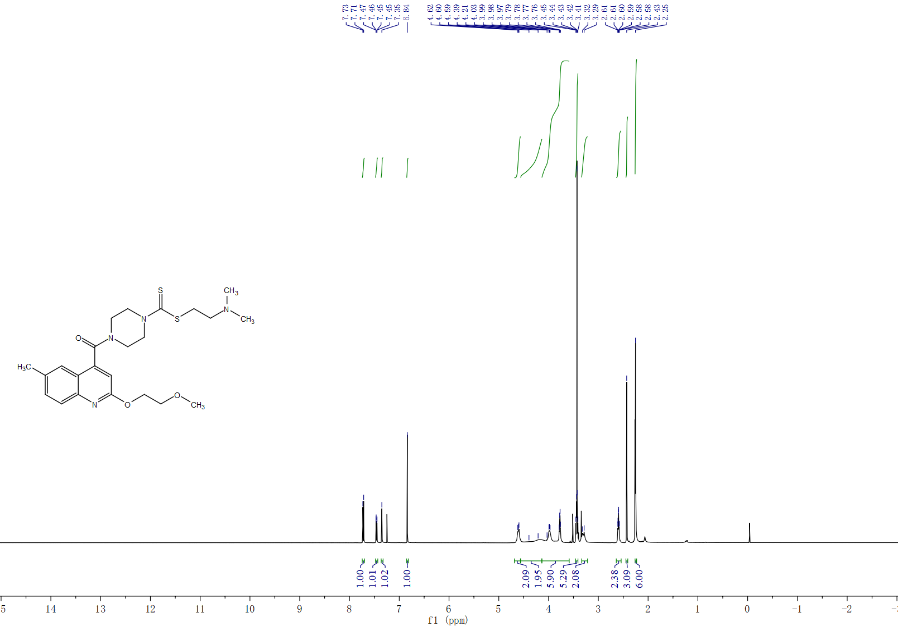


^1^H NMR of Compound **F1**


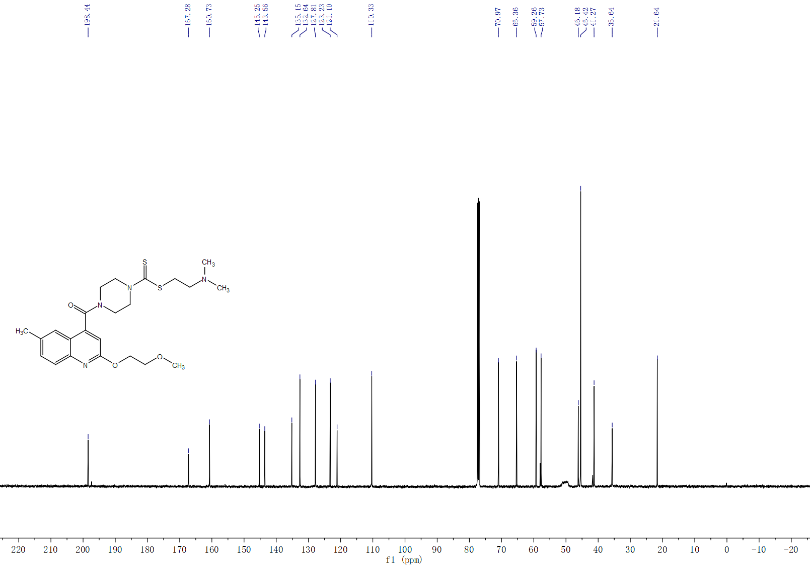


^13^C NMR of Compound **F1**

HRMS of Compound **F1**


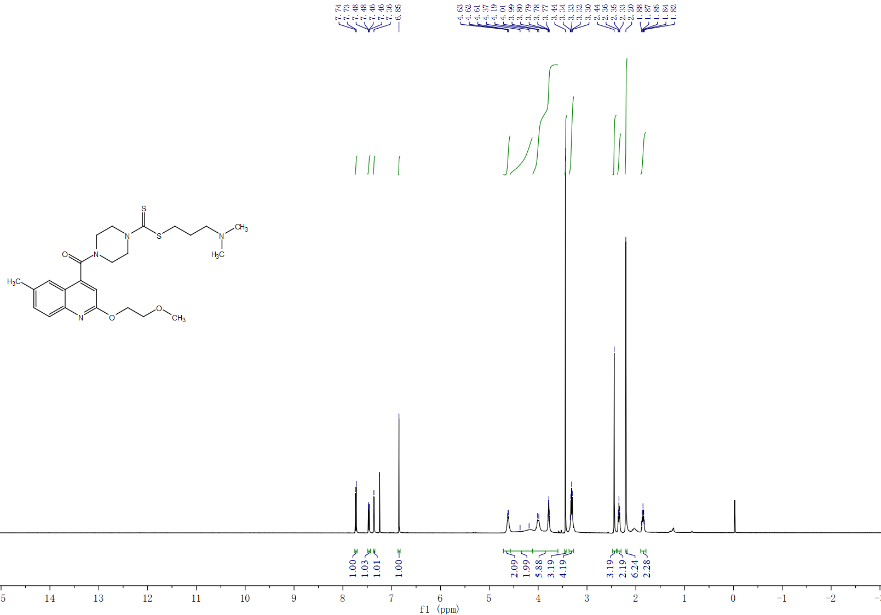


^1^H NMR of Compound **F2**


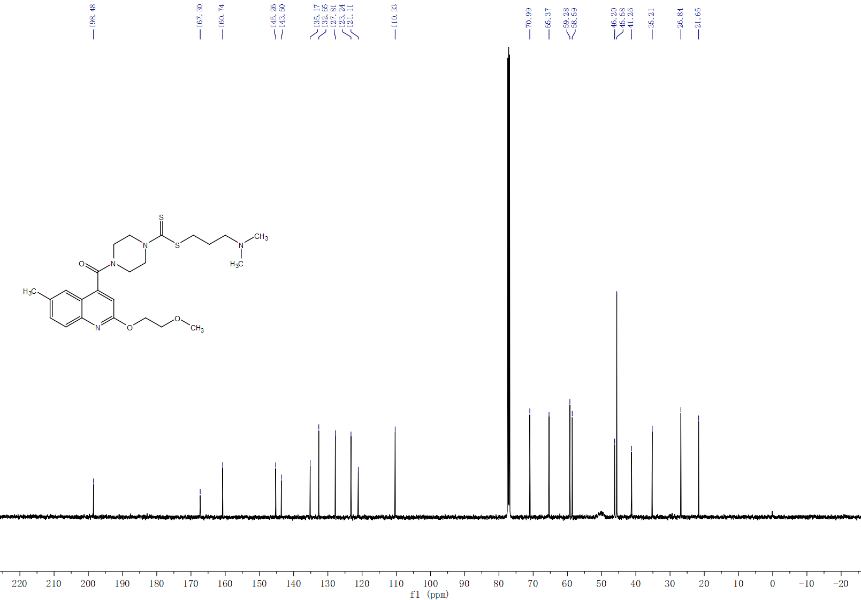


^13^C NMR of Compound **F2**

HRMS of Compound **F2**


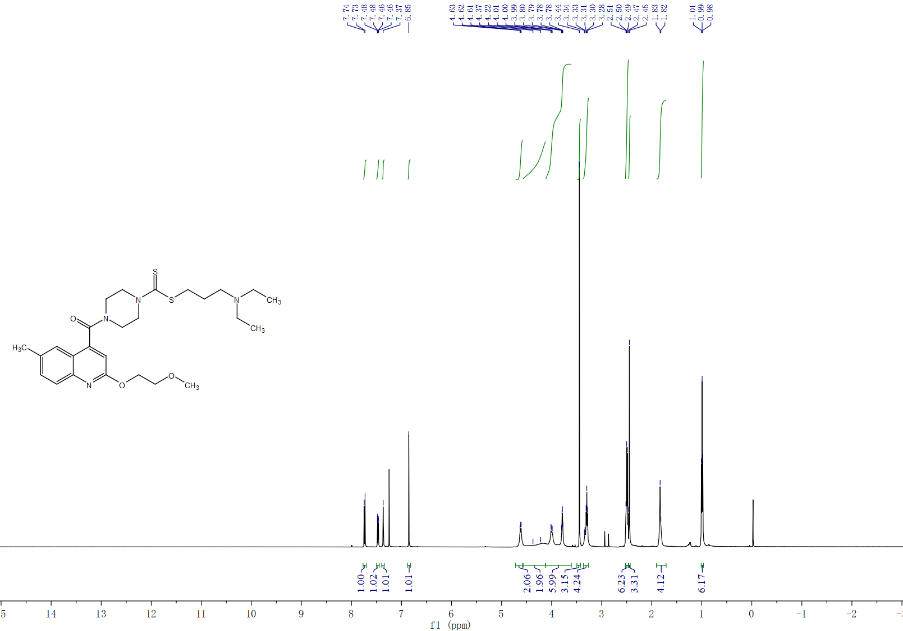


^1^H NMR of Compound **F3**


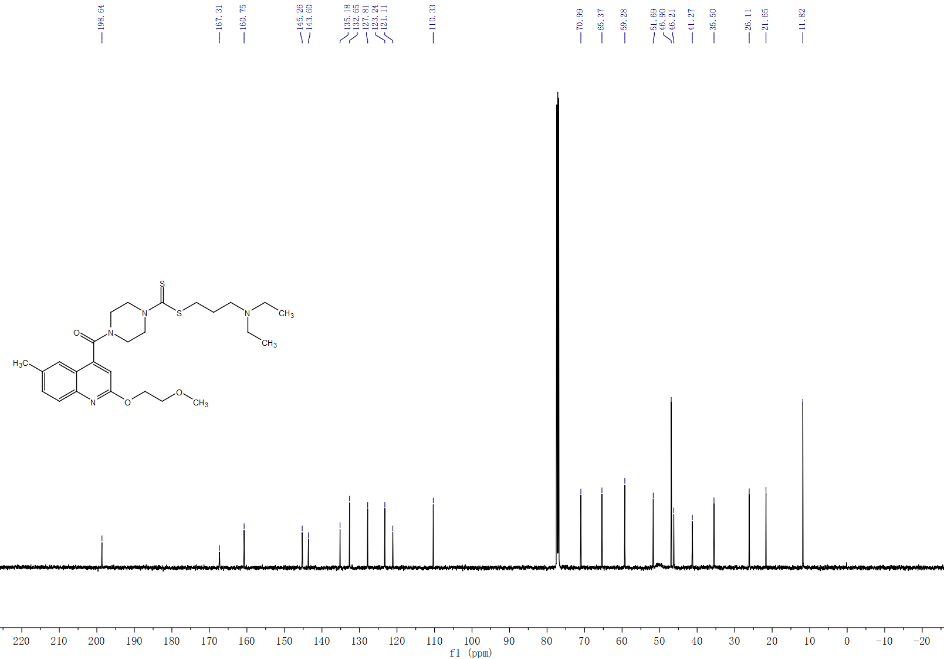


^13^C NMR of Compound **F3**

HRMS of Compound **F3**


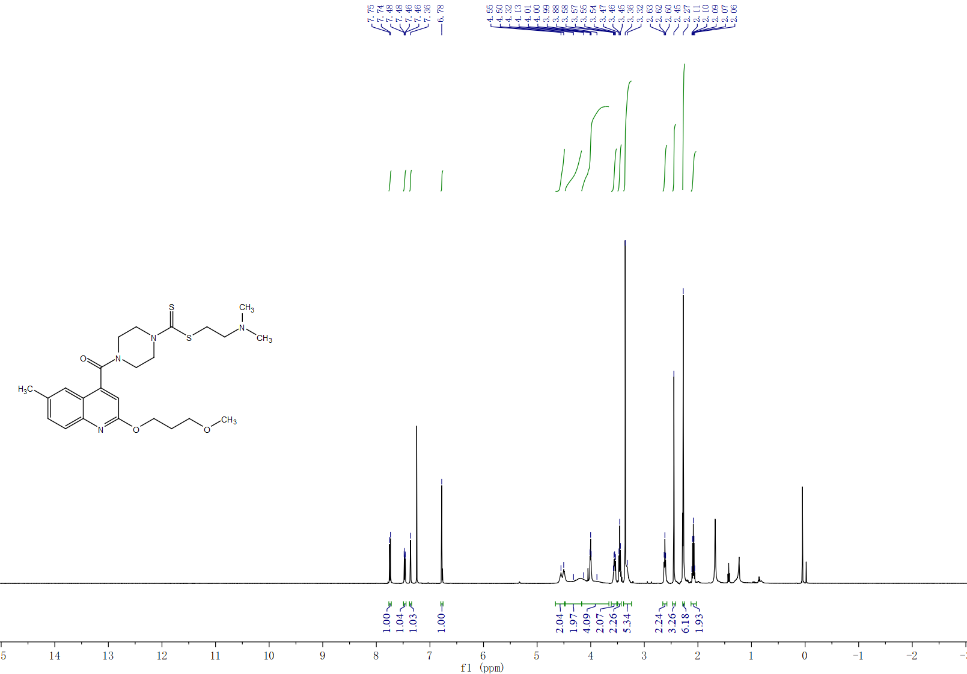


^1^H NMR of Compound **F4**


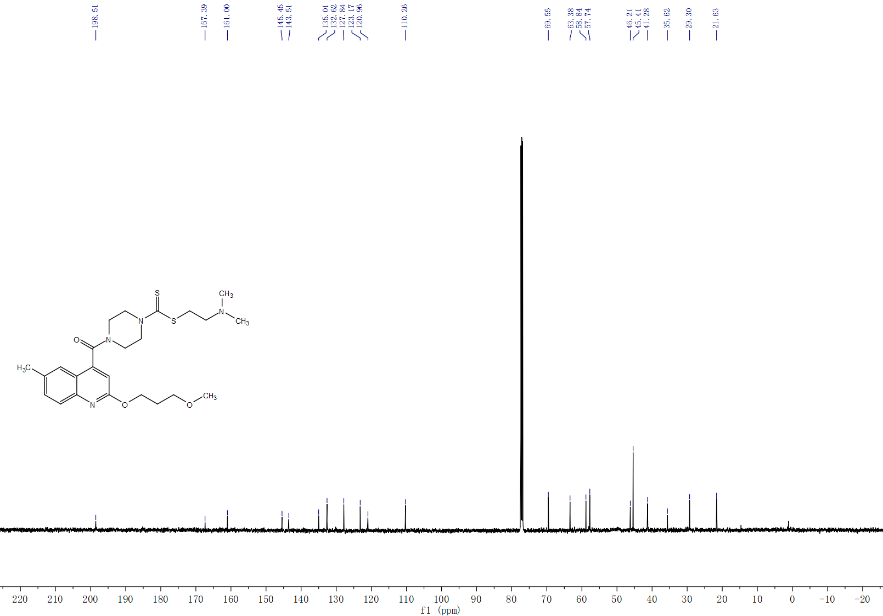


^13^C NMR of Compound **F4**

HRMS of Compound **F4**


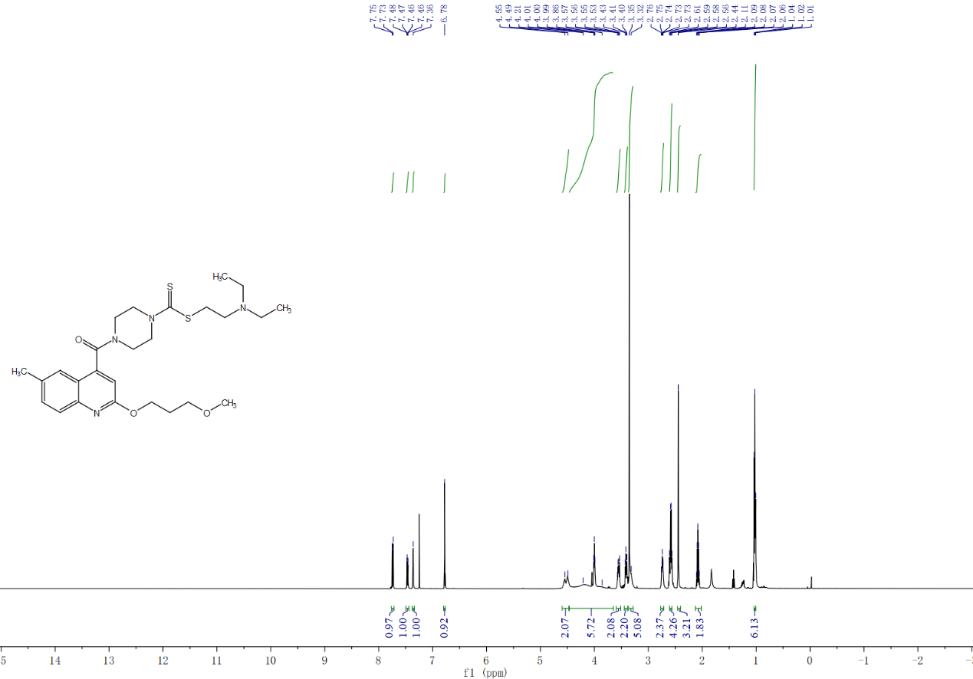


^1^H NMR of Compound **F5**


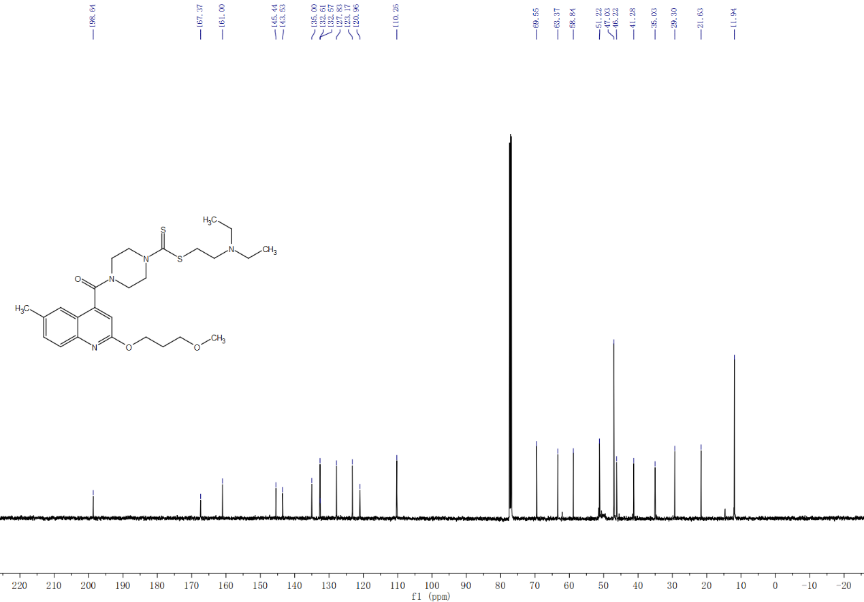


^13^C NMR of Compound **F5**

HRMS of Compound **F5**


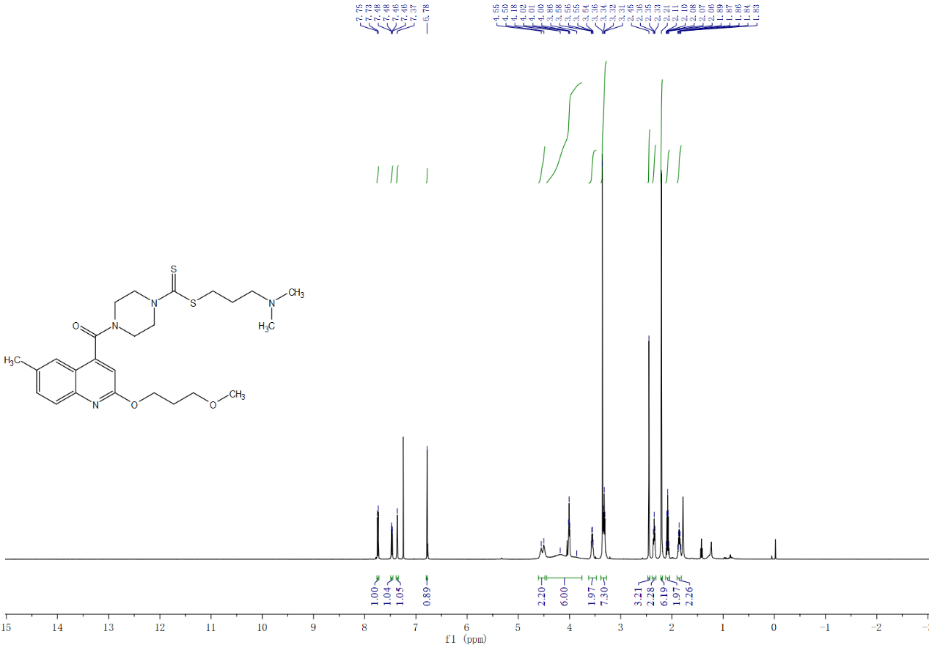


^1^H NMR of Compound **F6**


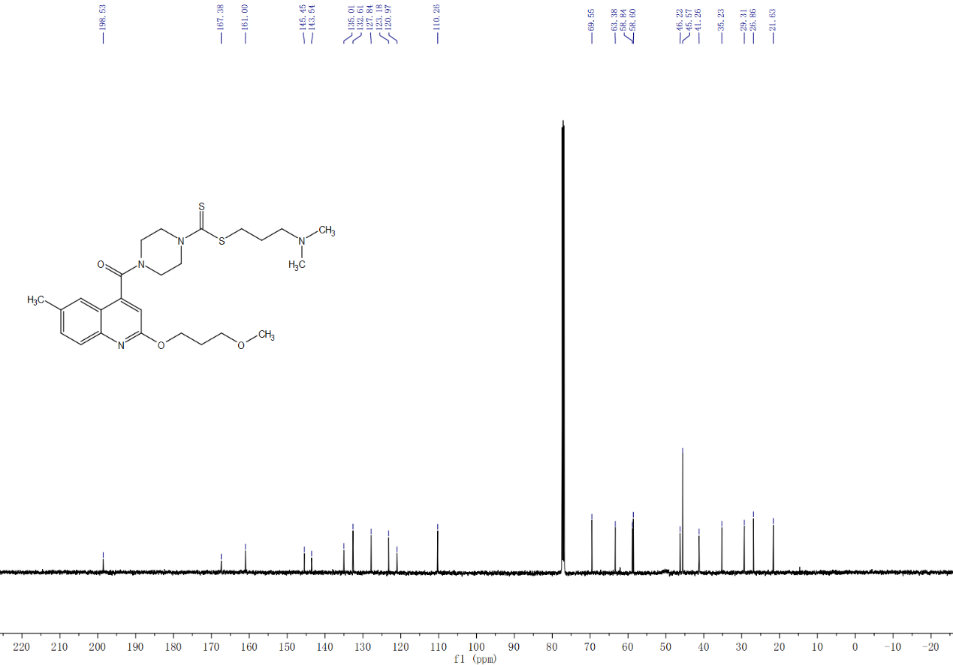


^13^C NMR of Compound **F6**

HRMS of Compound **F6**


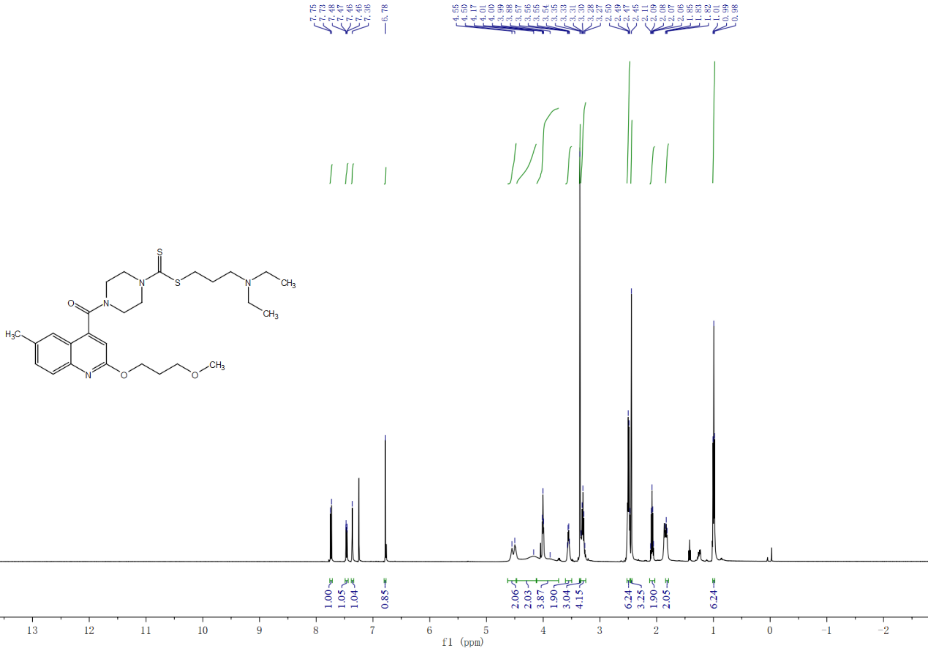


^1^H NMR of Compound **F7**


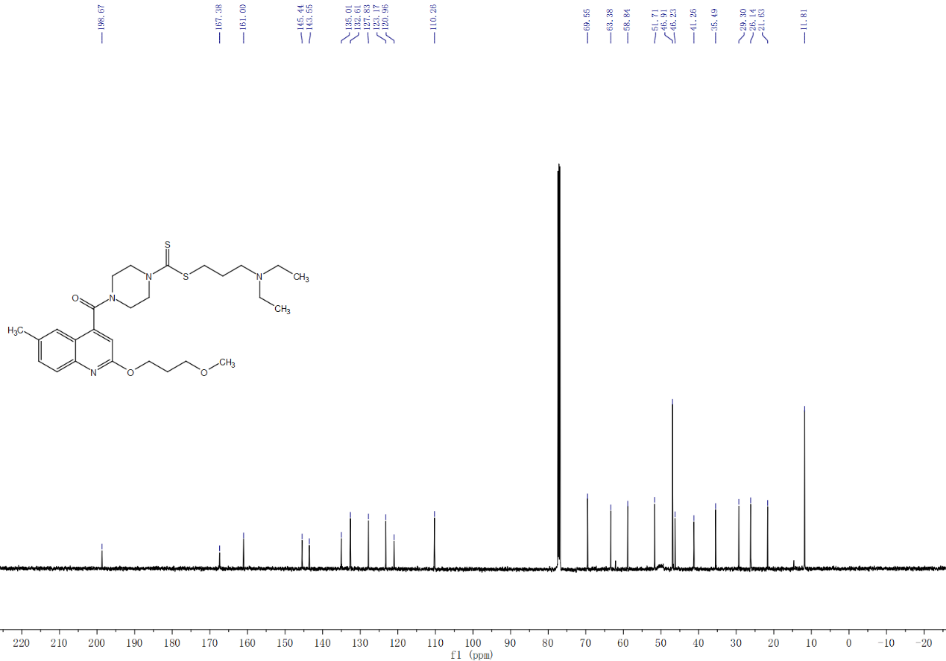


^13^C NMR of Compound **F7**

HRMS of Compound **F7**


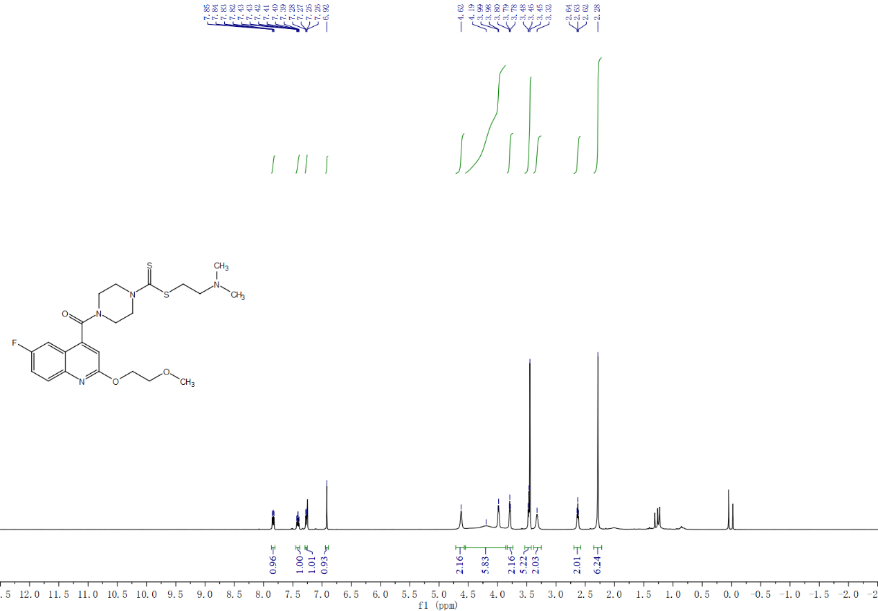


^1^H NMR of Compound **F8**


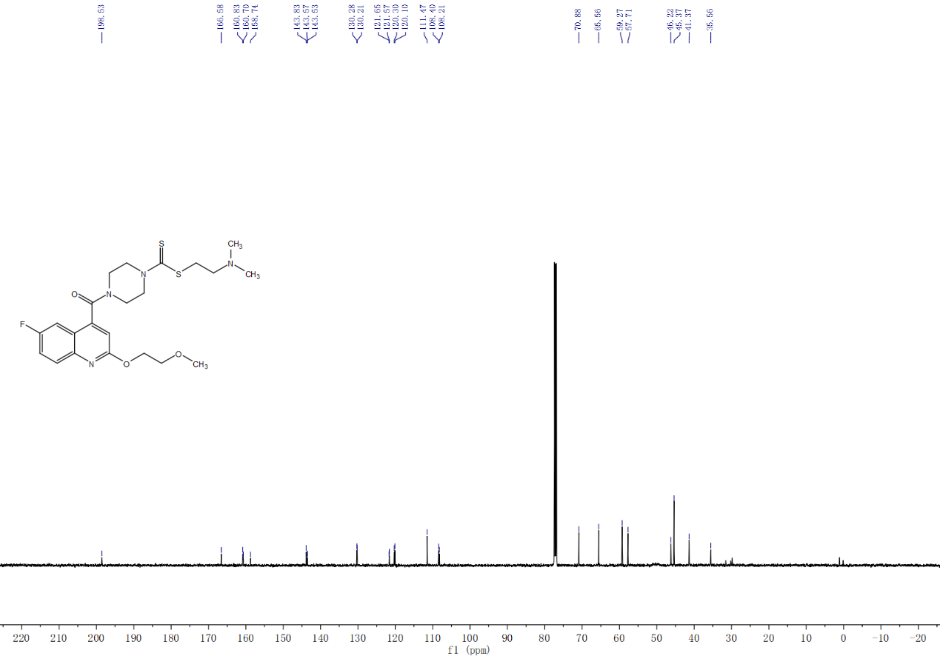


^13^C NMR of Compound **F8**

HRMS of Compound **F8**


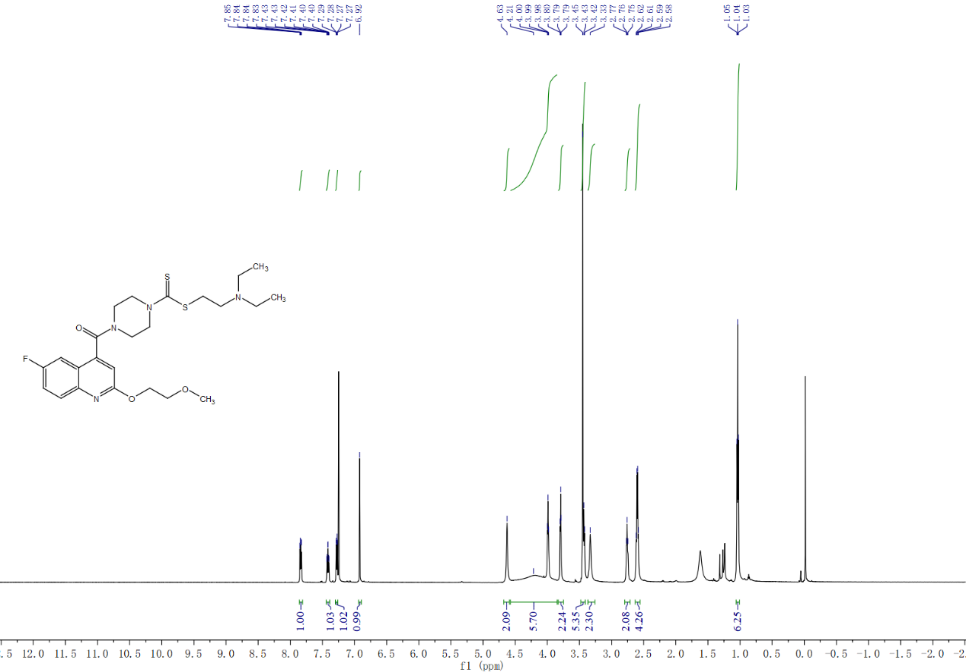


^1^H NMR of Compound **F9**


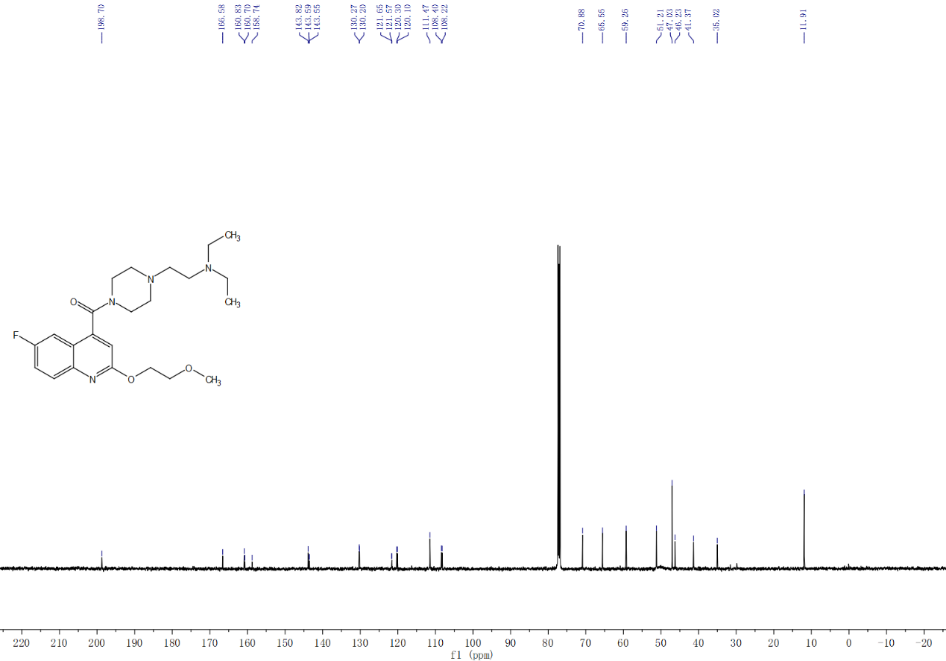


^13^C NMR of Compound **F9**

HRMS of Compound **F9**


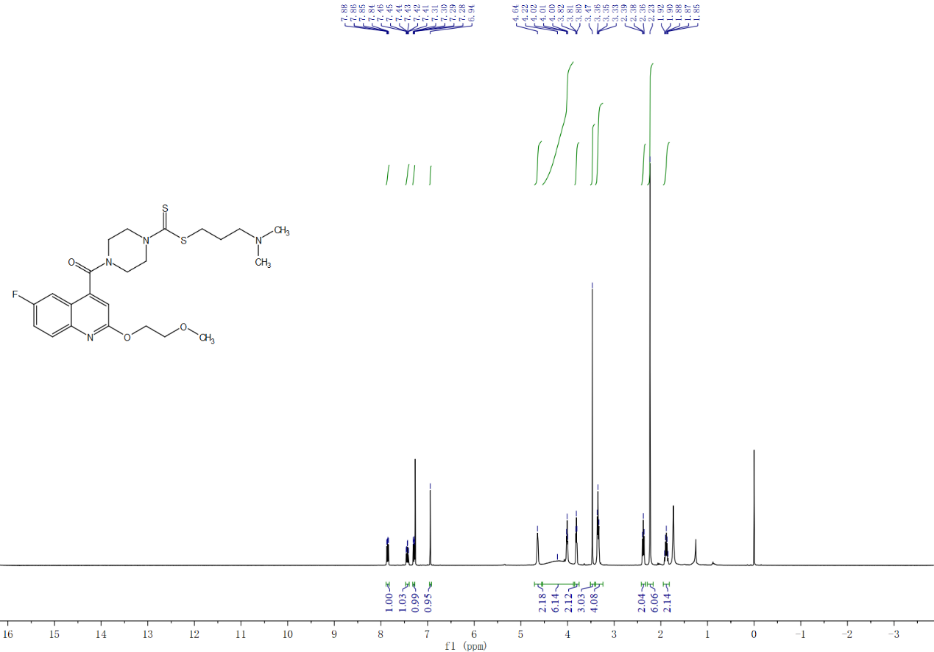


^1^H NMR of Compound **F10**


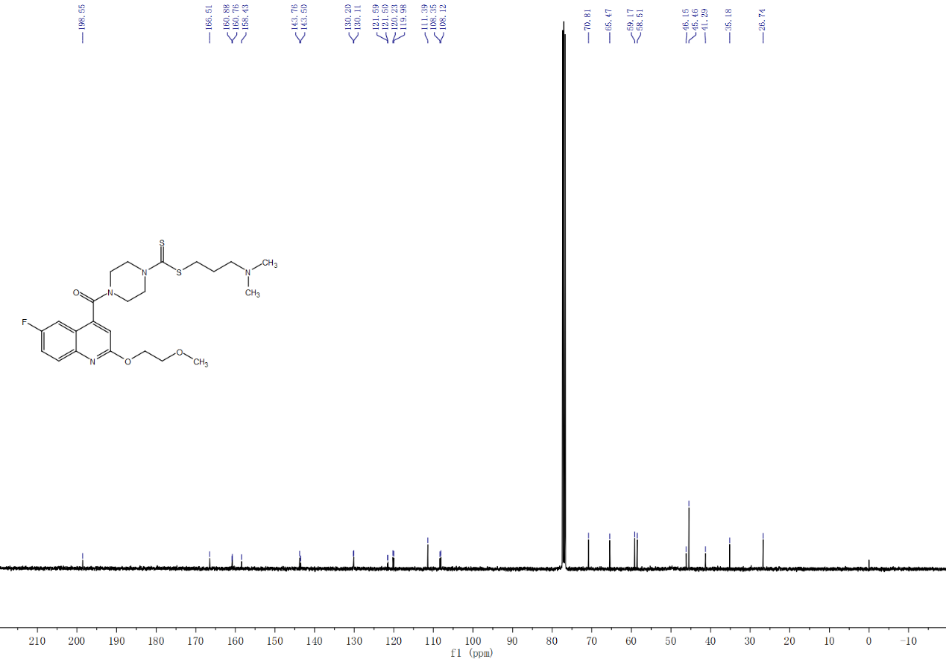


^13^C NMR of Compound **F10**

HRMS of Compound **F10**


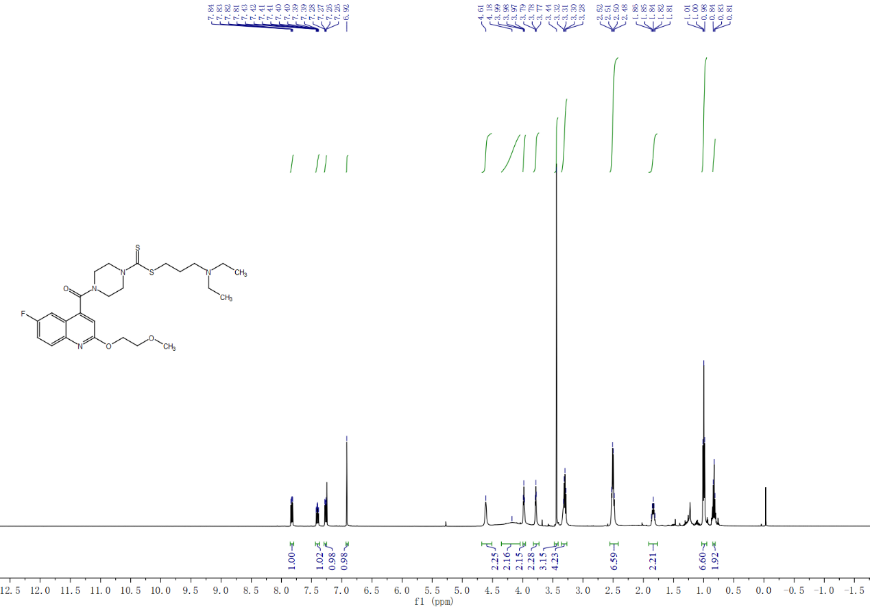


^1^H NMR of Compound **F11**


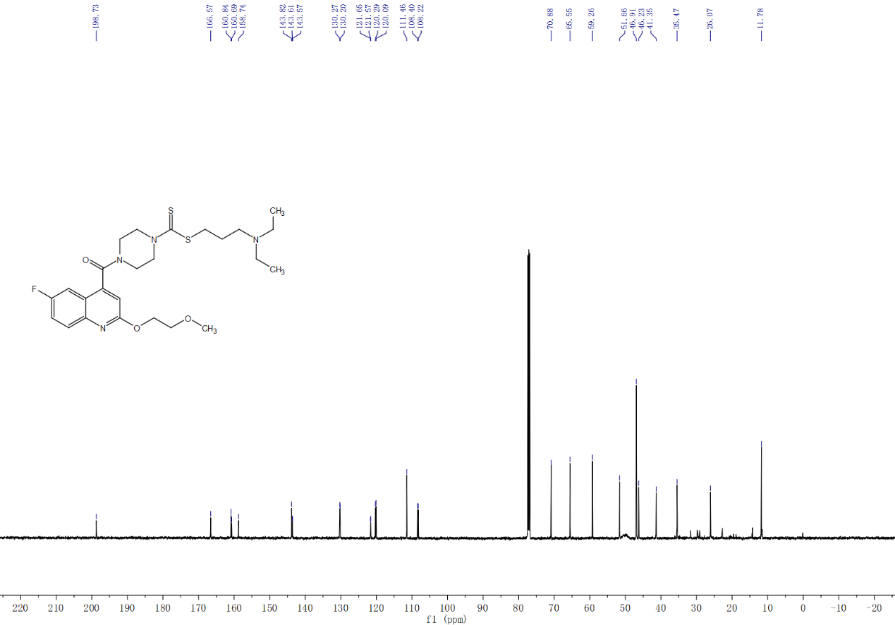


^13^C NMR of Compound **F11**

HRMS of Compound **F11**


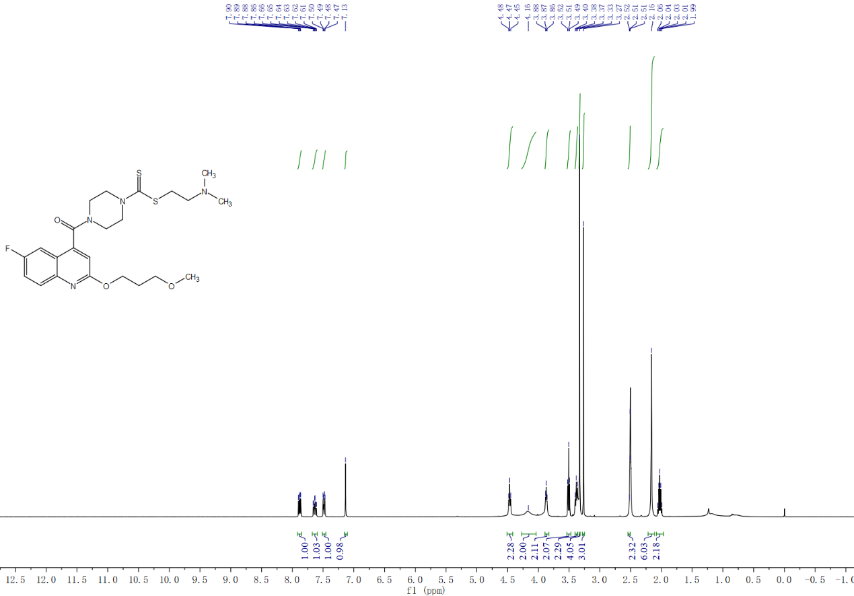


^1^H NMR of Compound **F12**


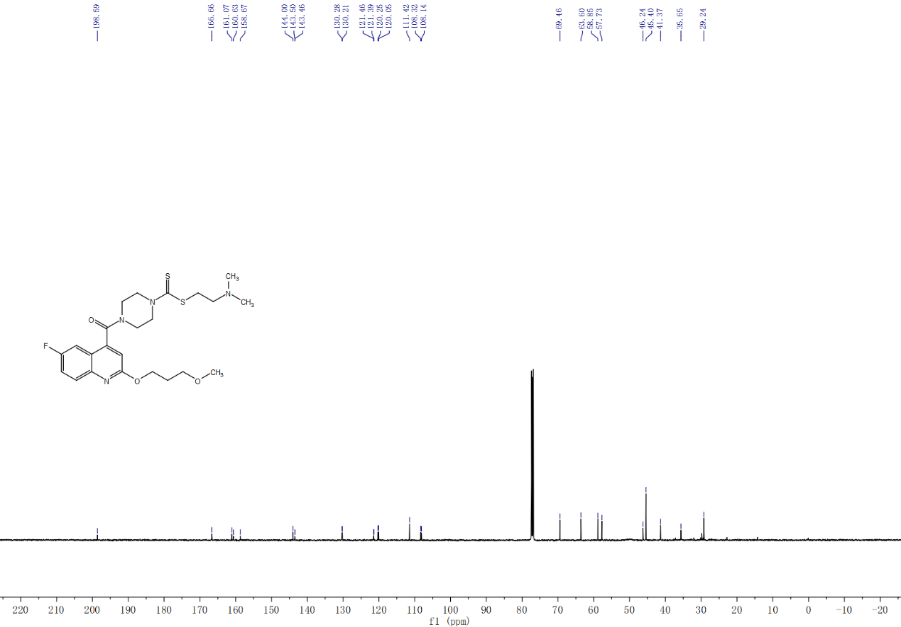


^13^C NMR of Compound **F12**

HRMS of Compound **F12**


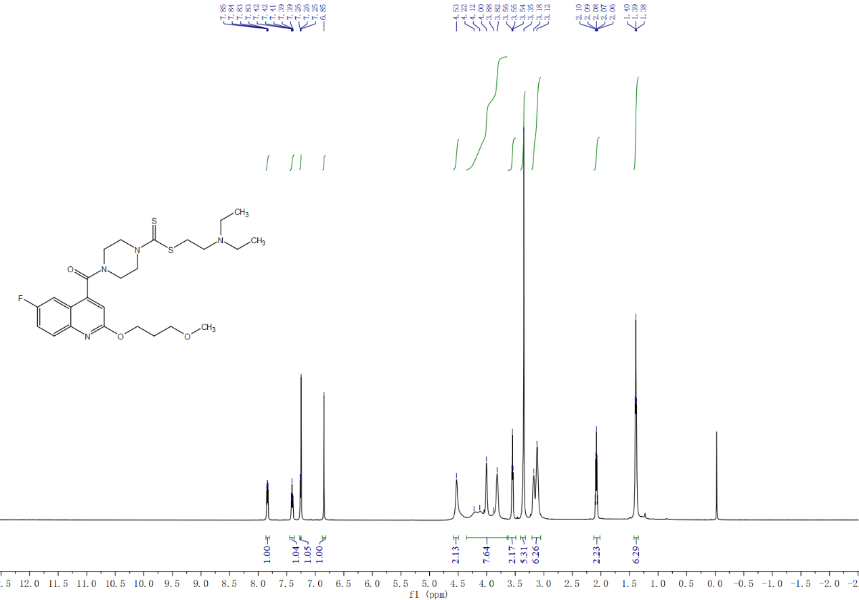


^1^H NMR of Compound **F13**


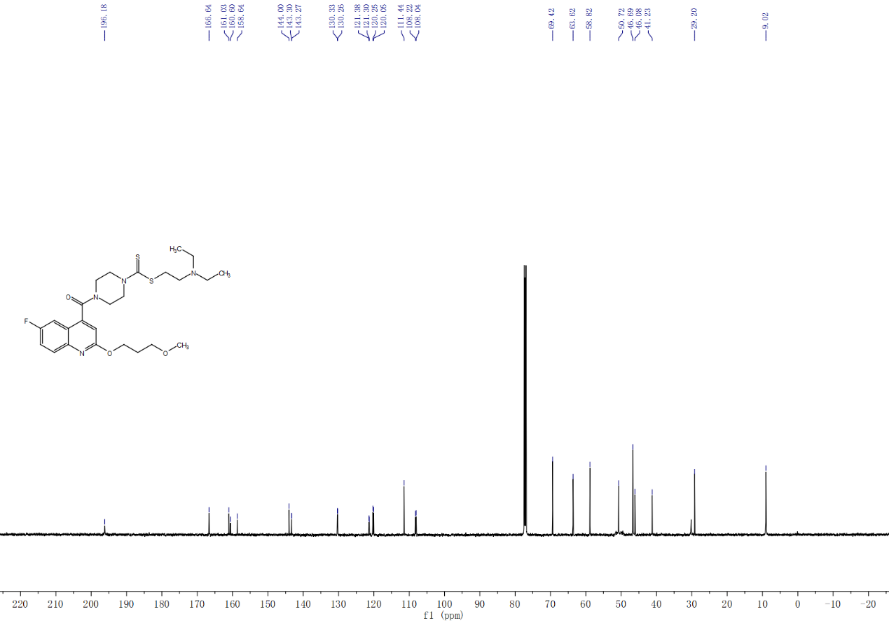


^13^C NMR of Compound **F13**

HRMS of Compound **F13**


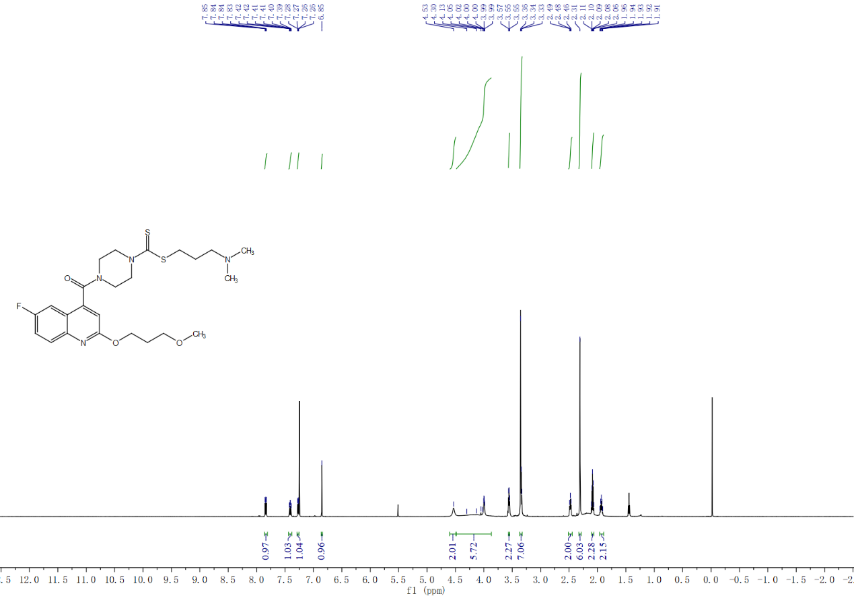


^1^H NMR of Compound **F14**


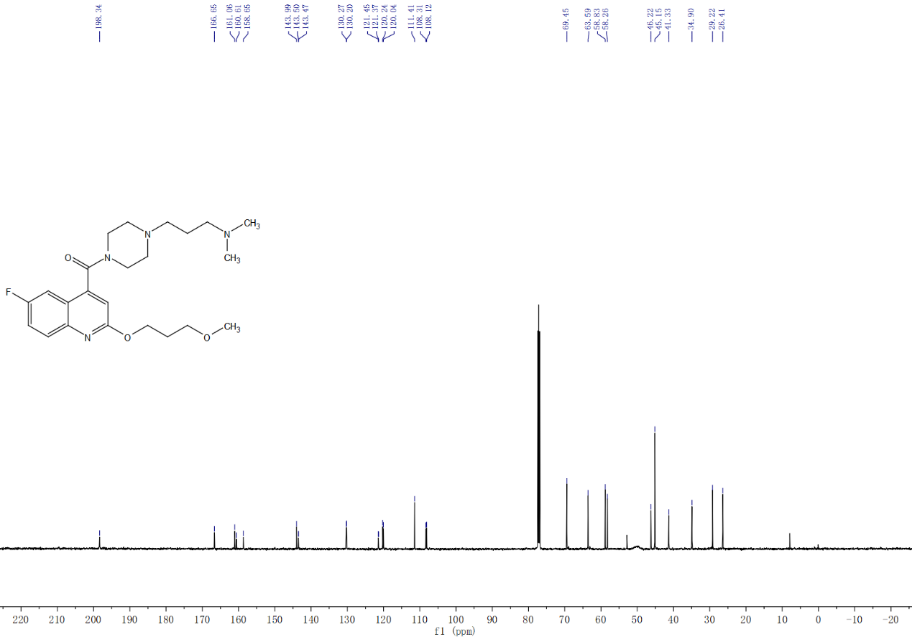


^13^C NMR of Compound **F14**

HRMS of Compound **F14**


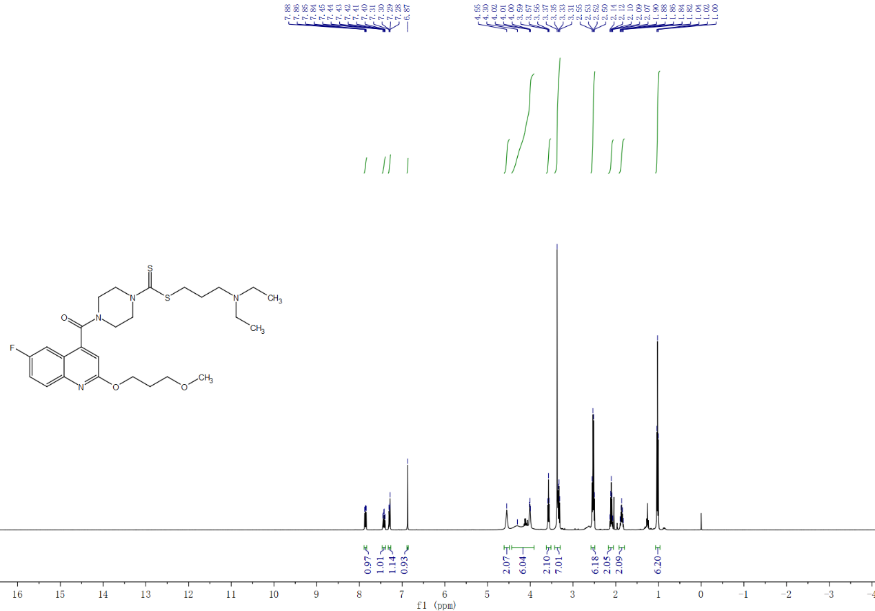


^1^H NMR of Compound **F15**


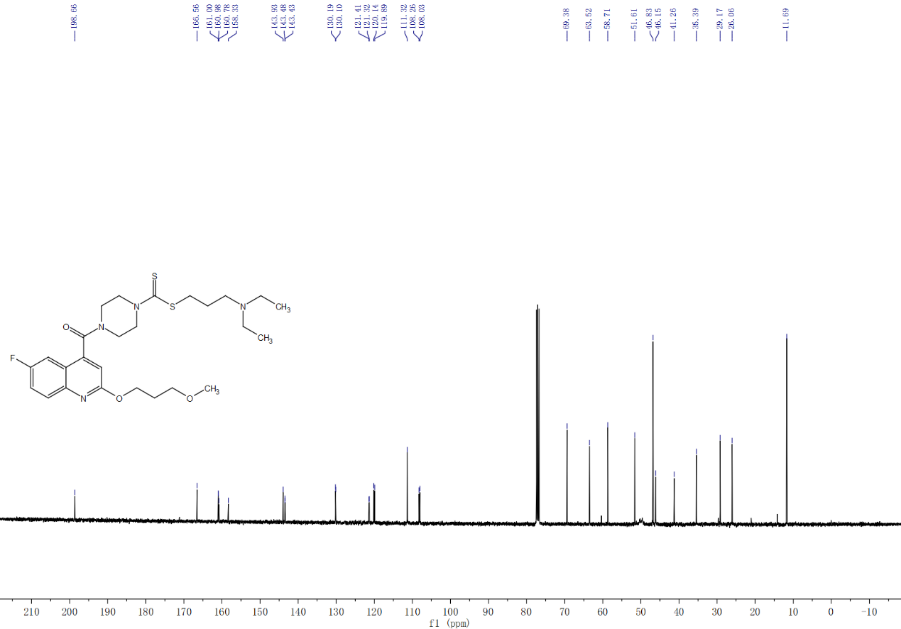


^13^C NMR of Compound **F15**

HRMS of Compound **F15**


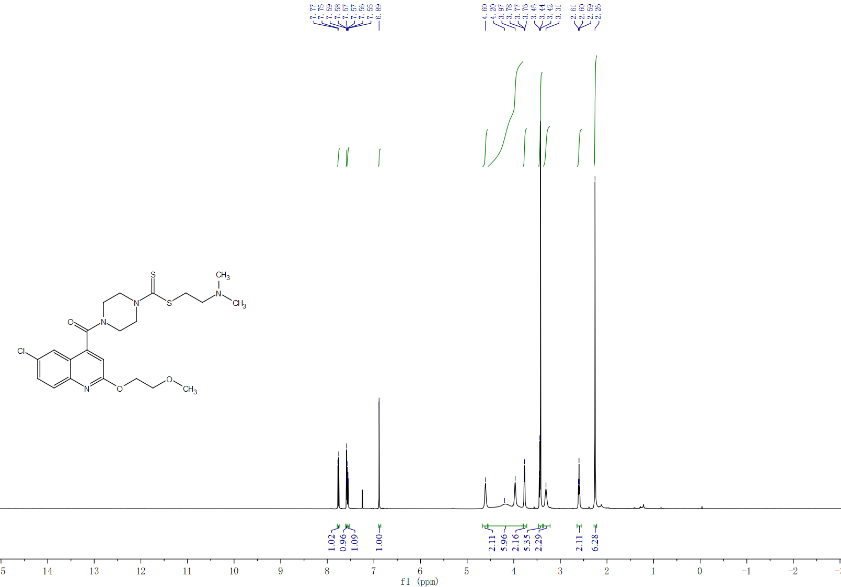


^1^H NMR of Compound **F16**


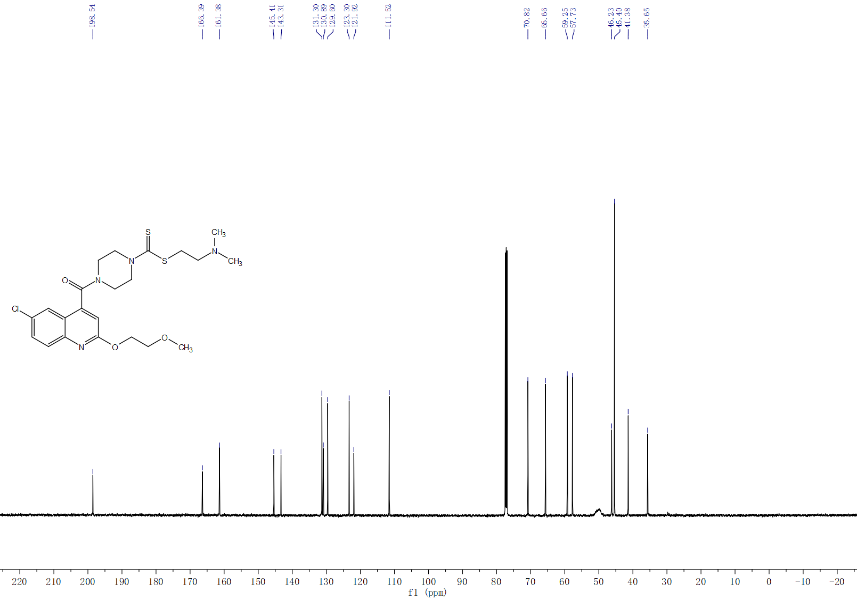


^13^C NMR of Compound **F16**

HRMS of Compound **F16**


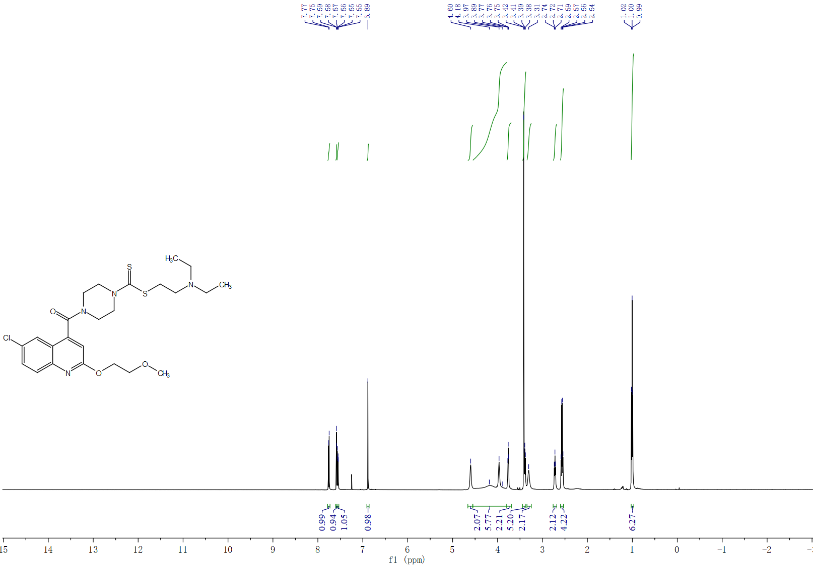


^1^H NMR of Compound **F17**


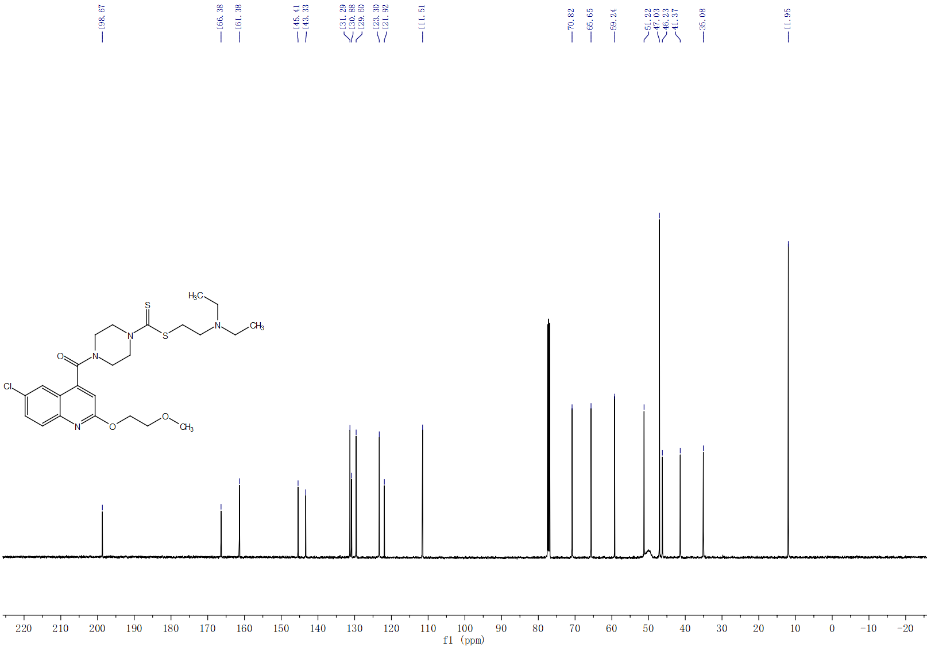


^13^C NMR of Compound **F17**

HRMS of Compound **F17**


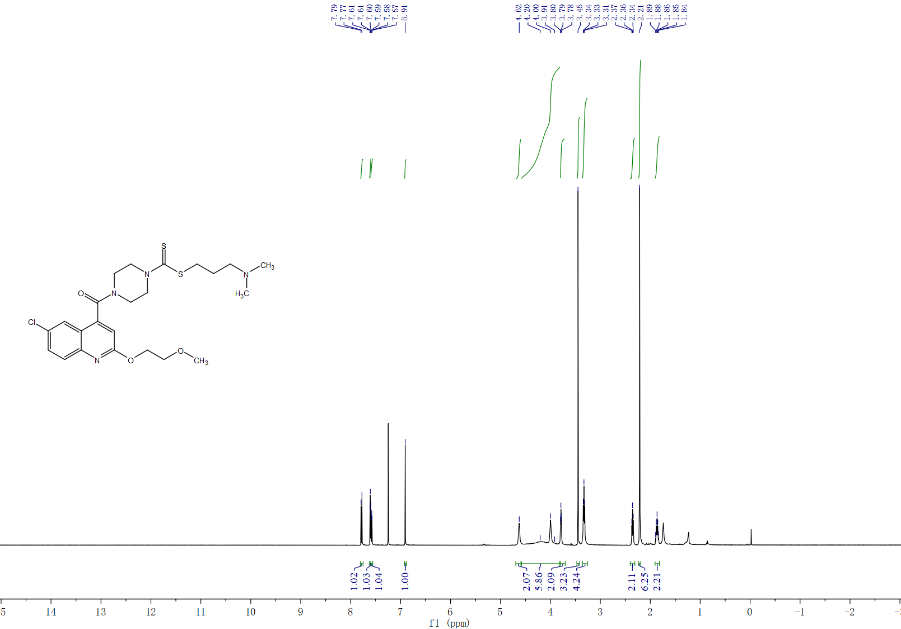


^1^H NMR of Compound **F18**


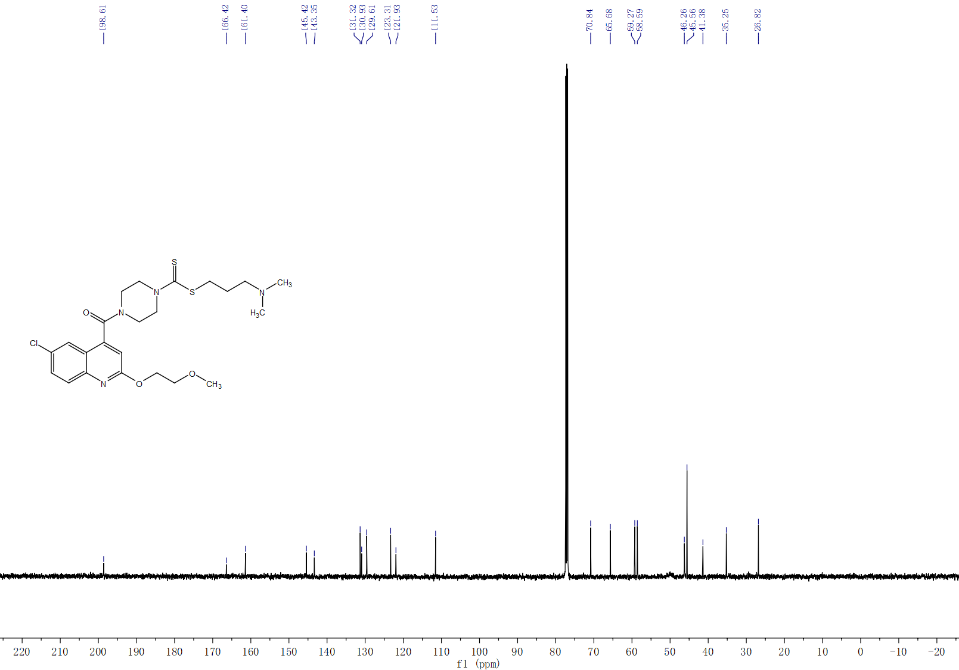


^13^C NMR of Compound **F18**

HRMS of Compound **F18**


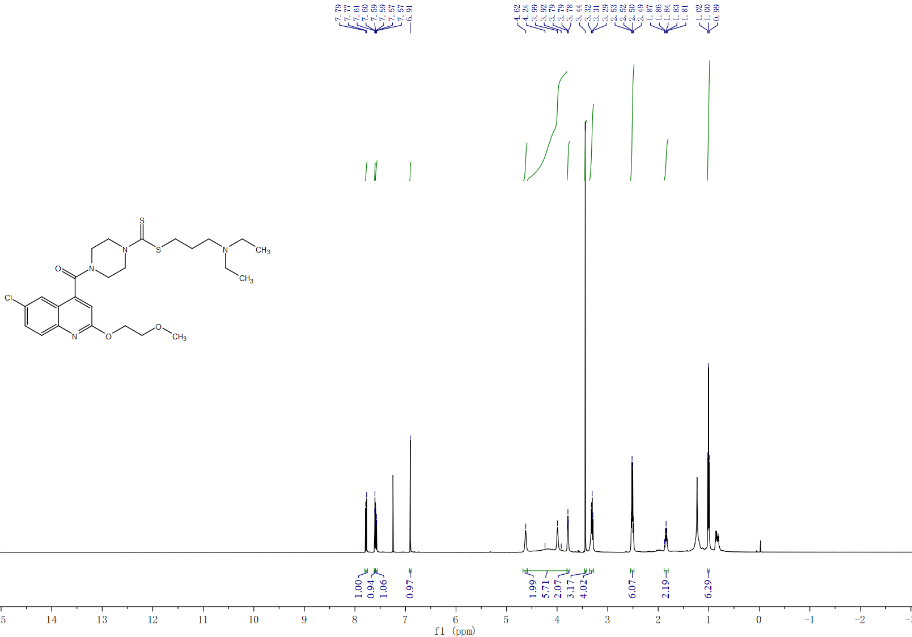


^1^H NMR of Compound **F19**


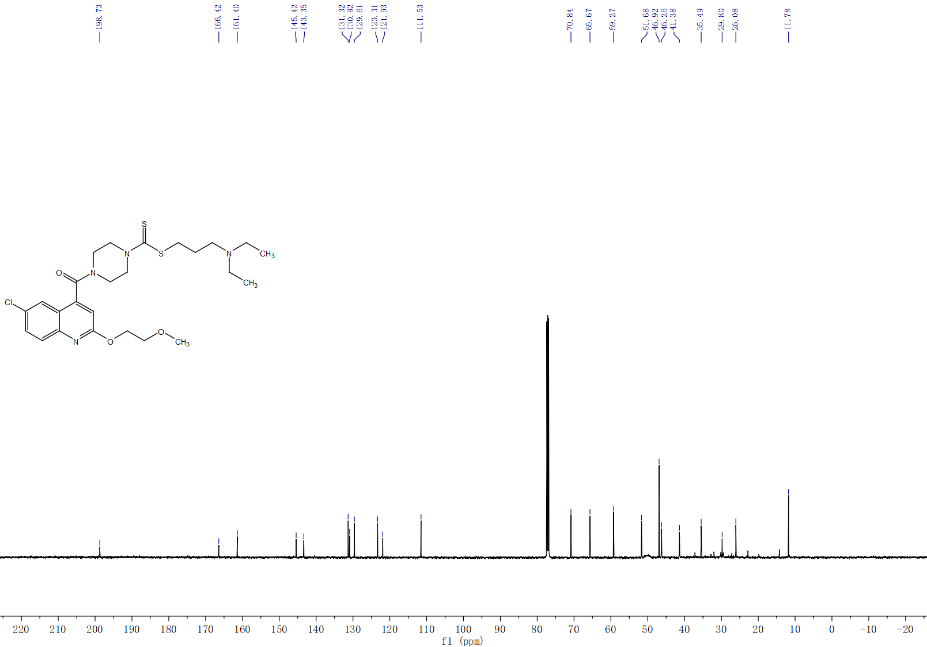


^13^C NMR of Compound **F19**

HRMS of Compound **F19**


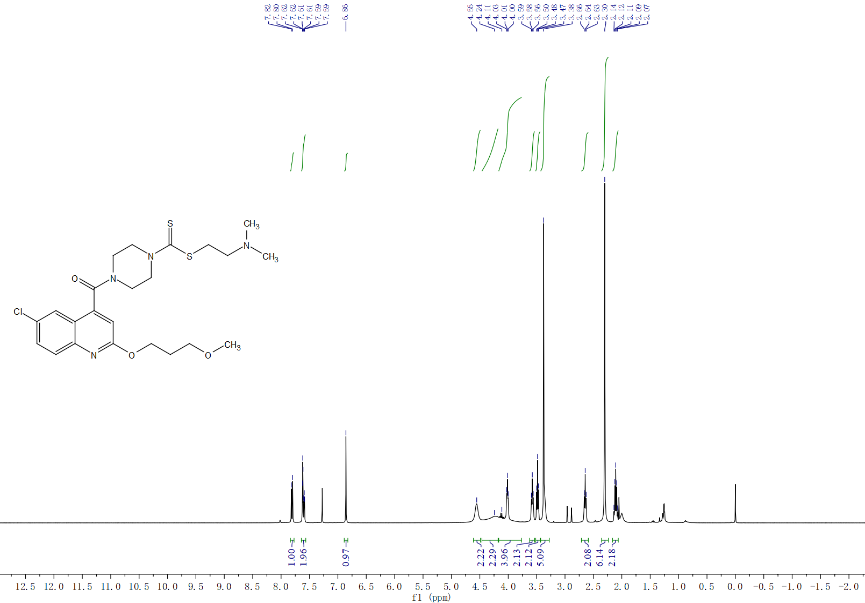


^1^H NMR of Compound **D20**


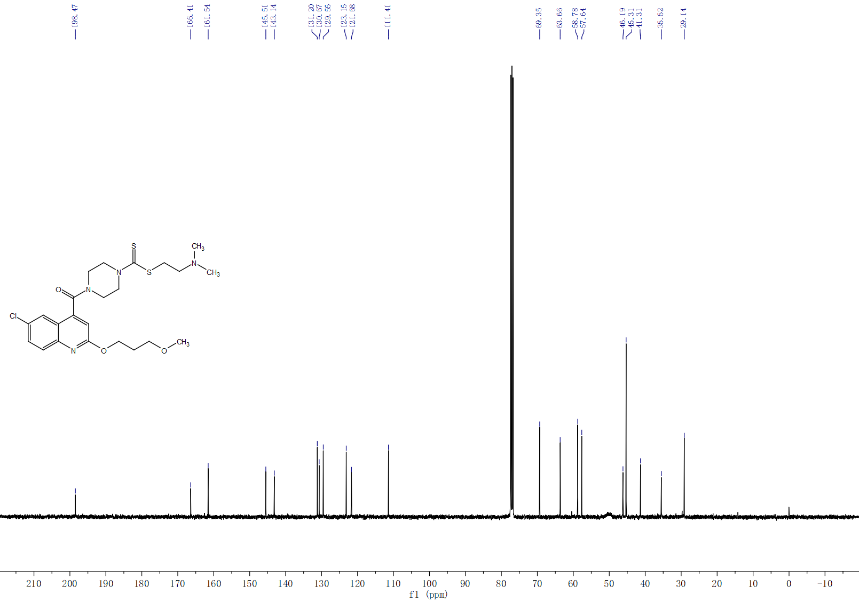


^13^C NMR of Compound **D20**

HRMS of Compound **F20**


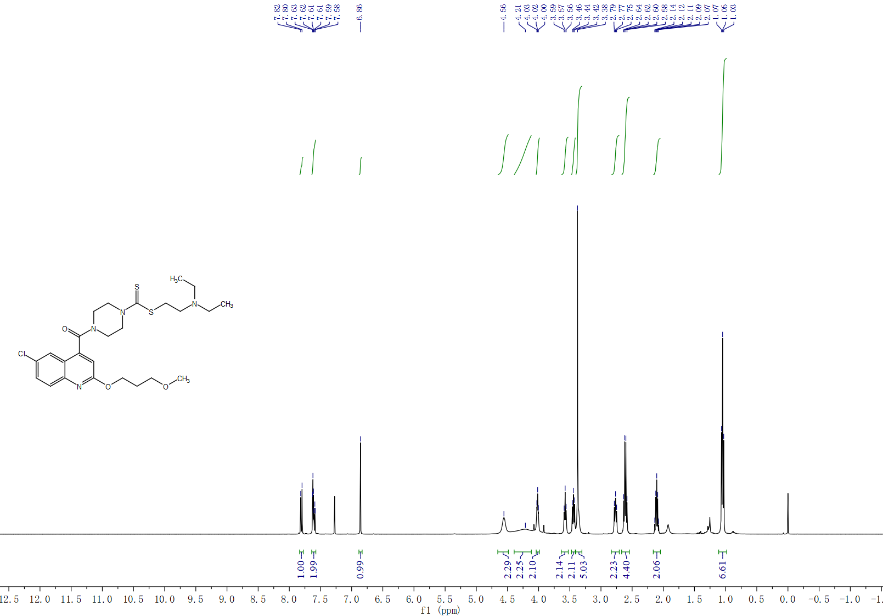


^1^H NMR of Compound **F21**


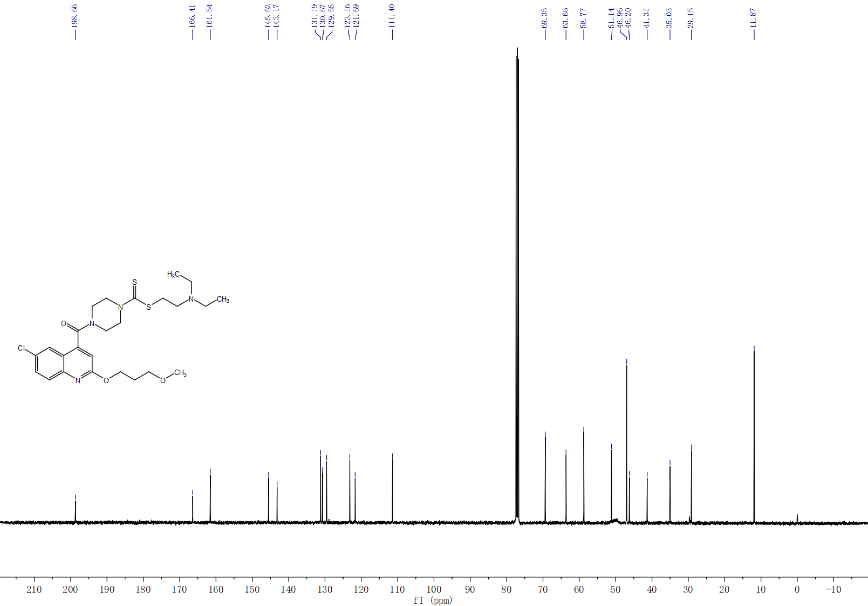


^13^C NMR of Compound **F21**

HRMS of Compound **F21**


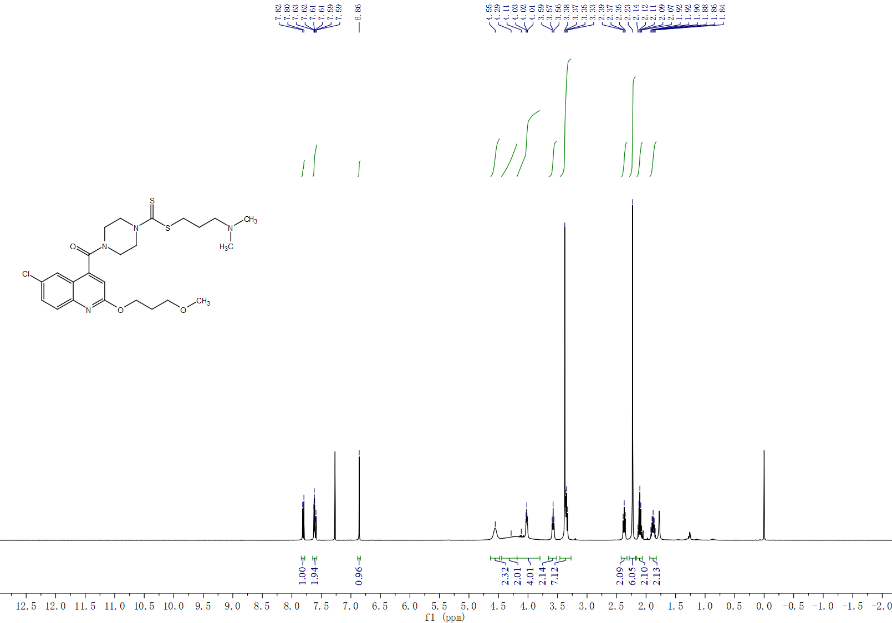


^1^H NMR of Compound **F22**


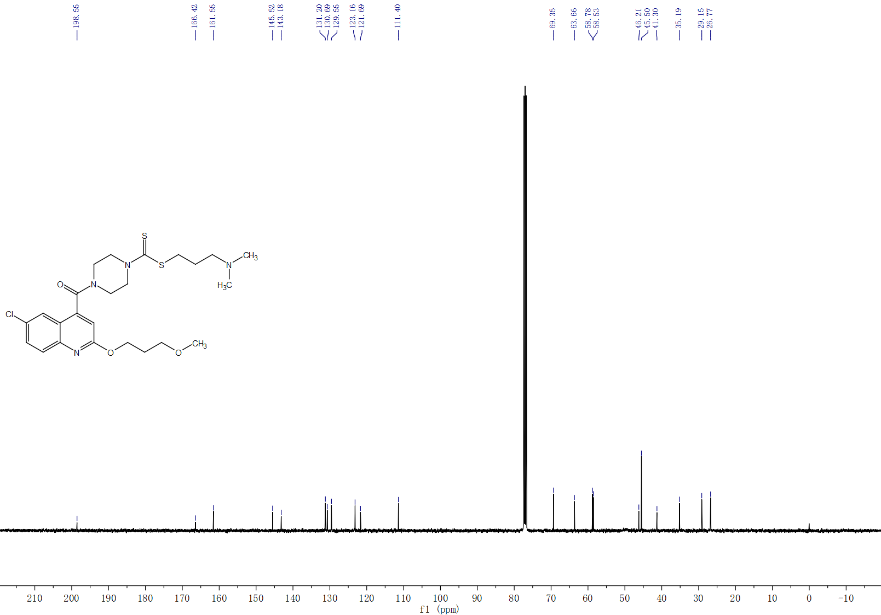


^13^C NMR of Compound **F22**

HRMS of Compound **F22**


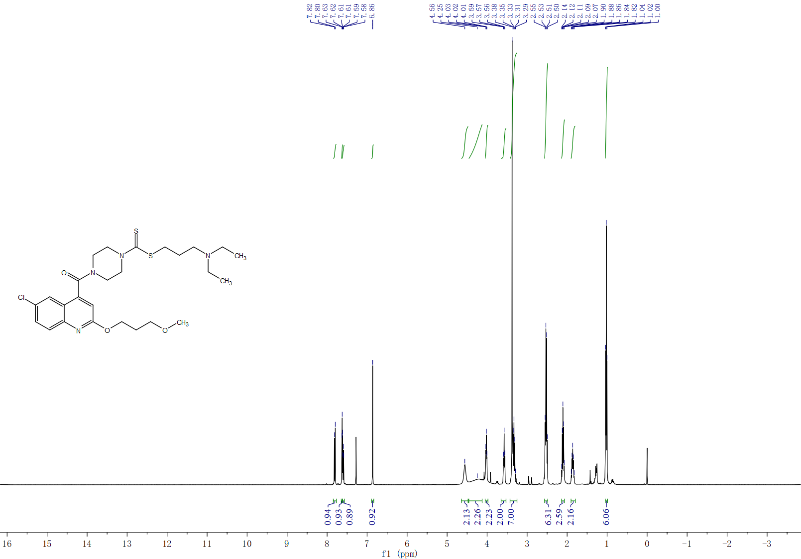


^1^H NMR of Compound **F23**


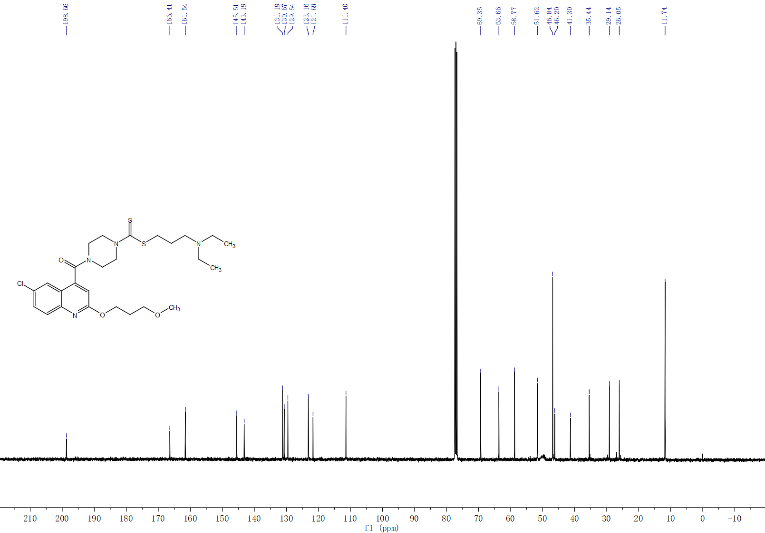


^13^C NMR of Compound **F23**

HRMS of Compound **F23**


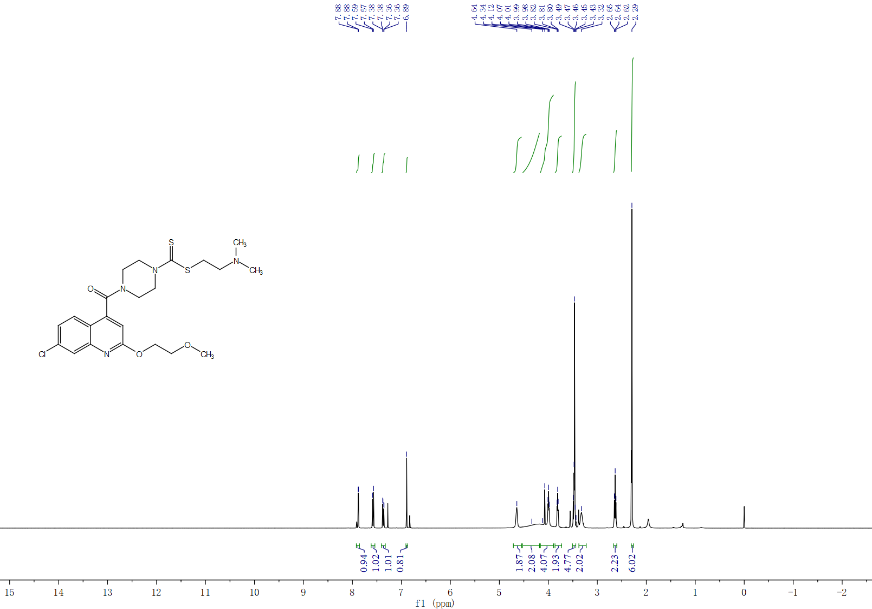


^1^H NMR of Compound **F24**


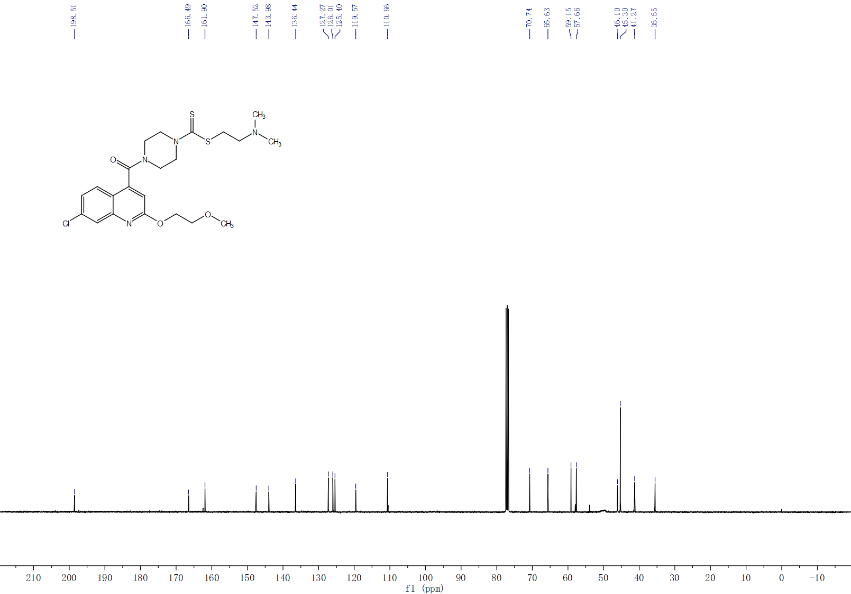


^13^C NMR of Compound **F24**

HRMS of Compound **F24**


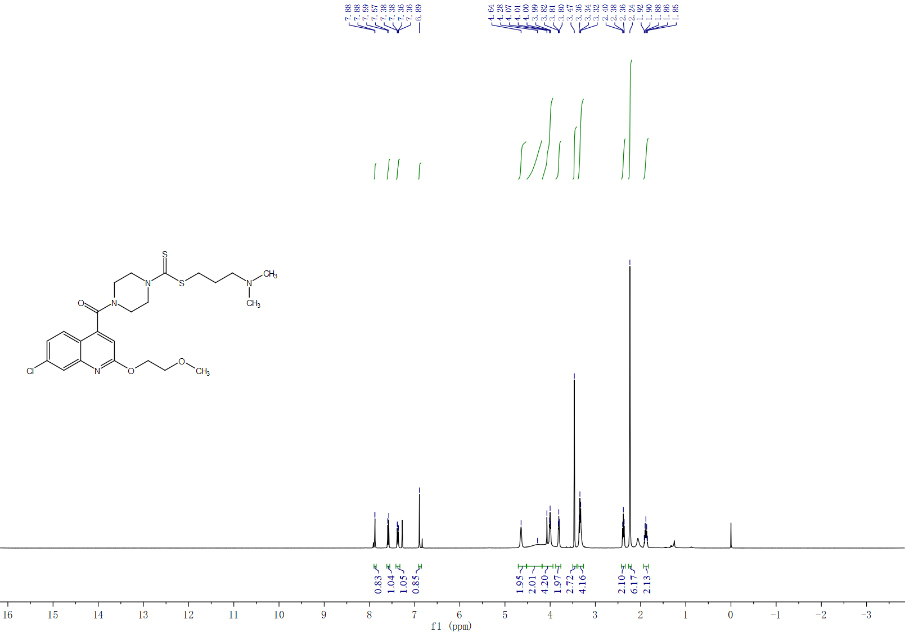


^1^H NMR of Compound **F25**


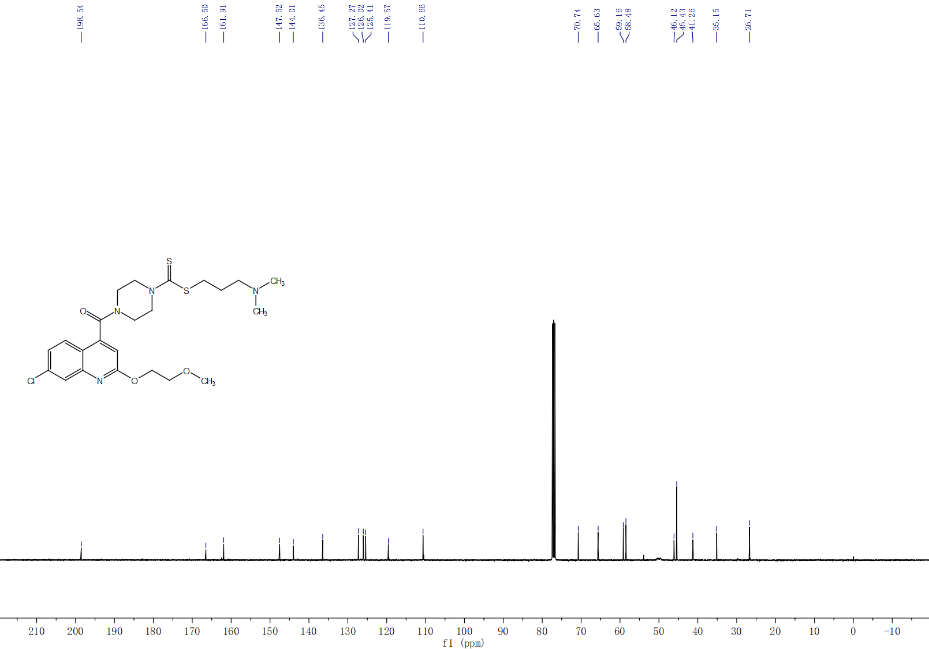


^13^C NMR of Compound **F25**

HRMS of Compound **F25**


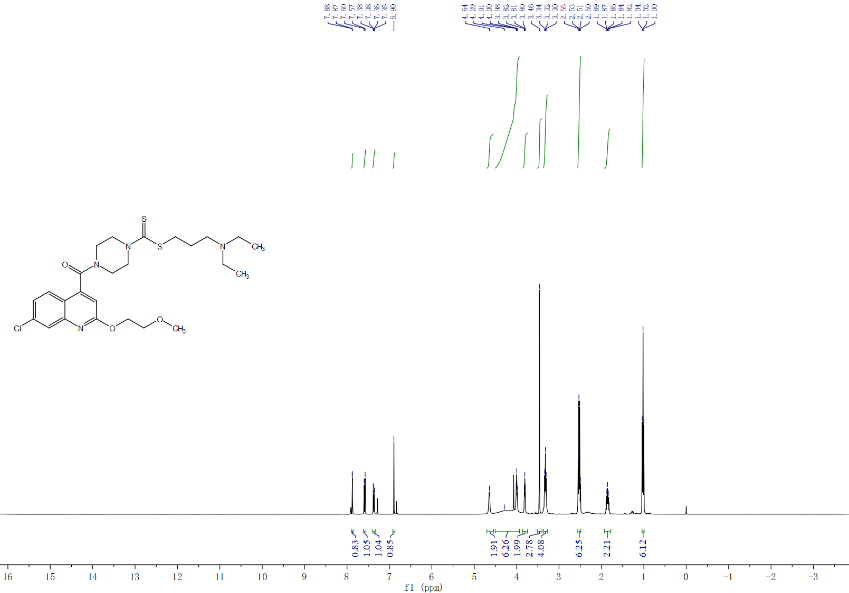


^1^H NMR of Compound **F26**


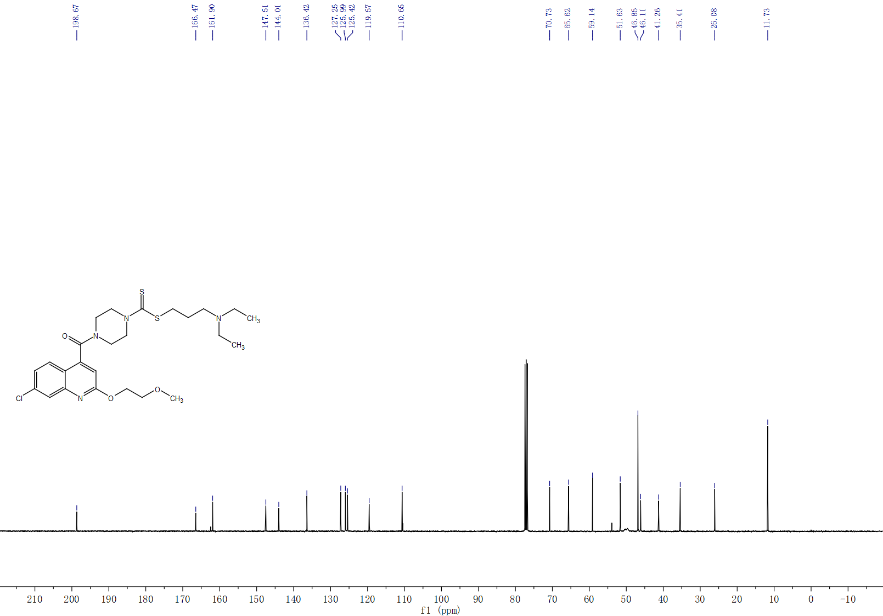


^13^C NMR of Compound **F26**

HRMS of Compound **F26**


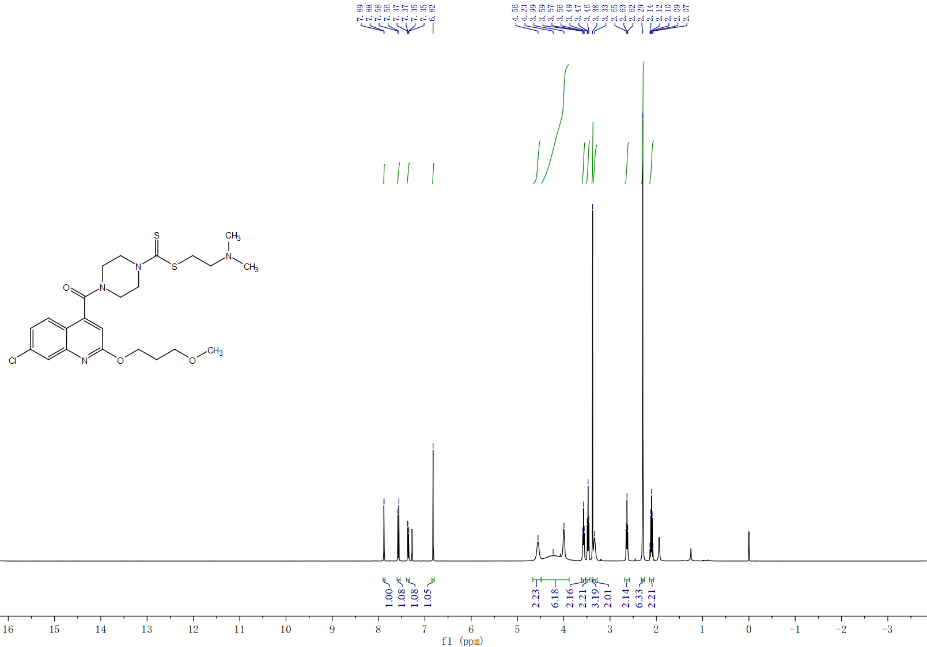


^1^H NMR of Compound **F27**


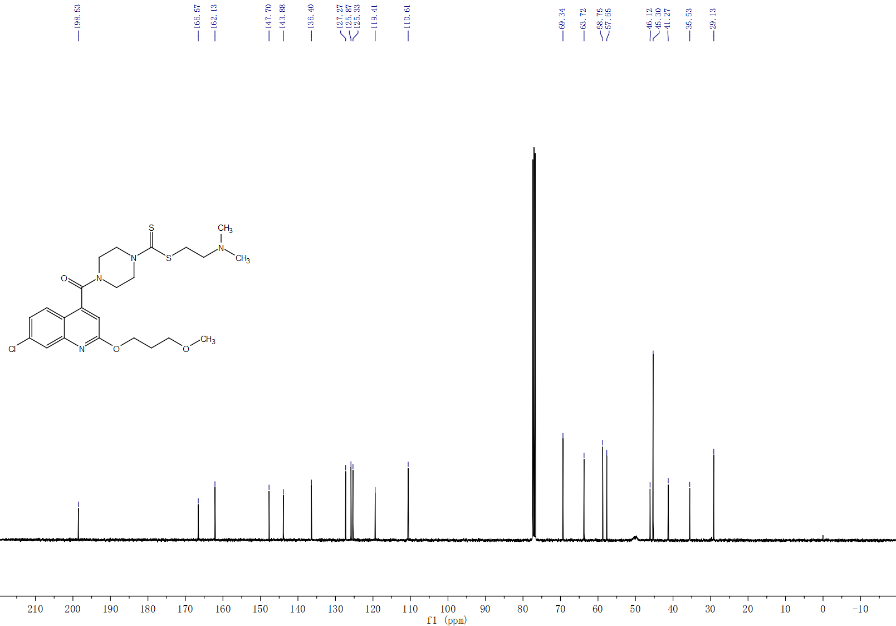


^13^C NMR of Compound **F27**

HRMS of Compound **F27**


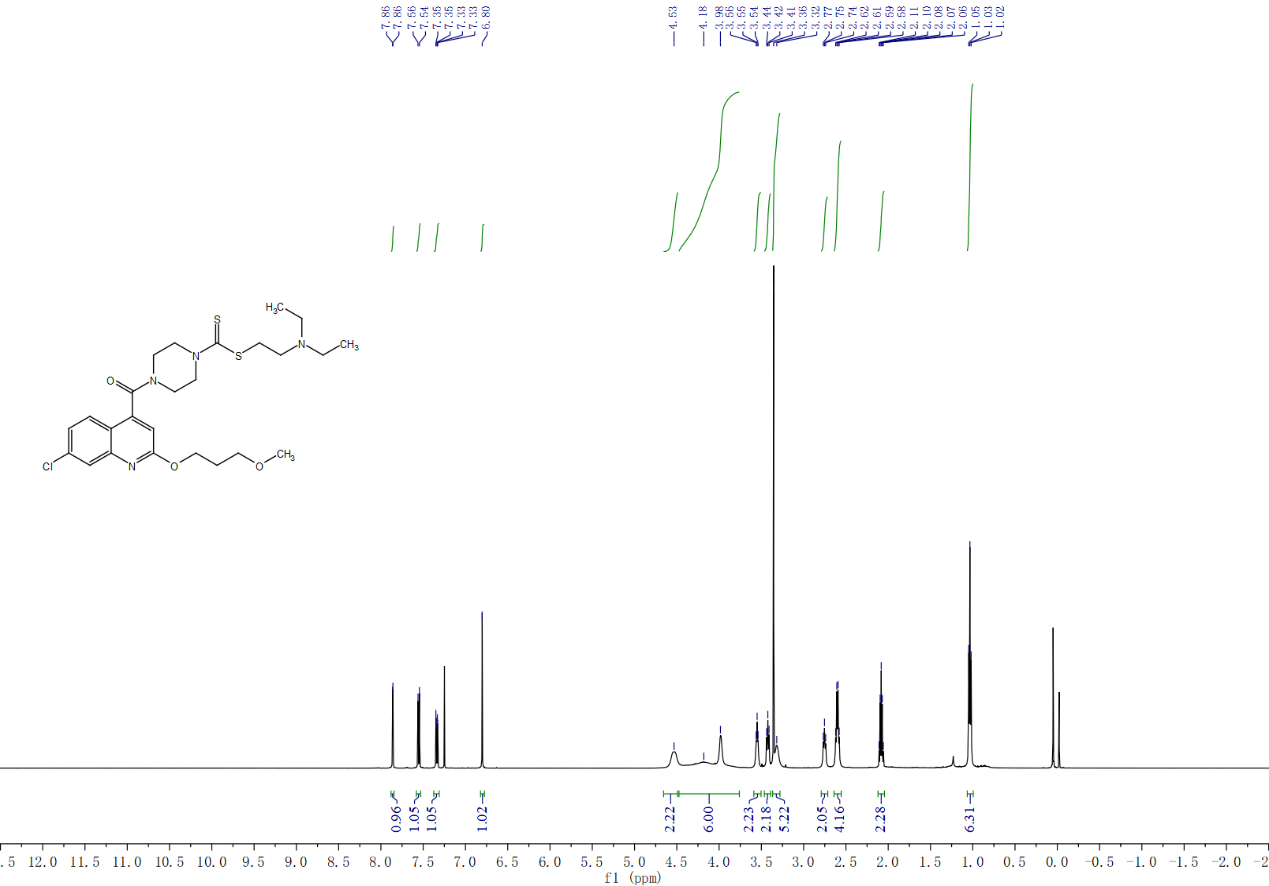


^1^H NMR of Compound **F28**


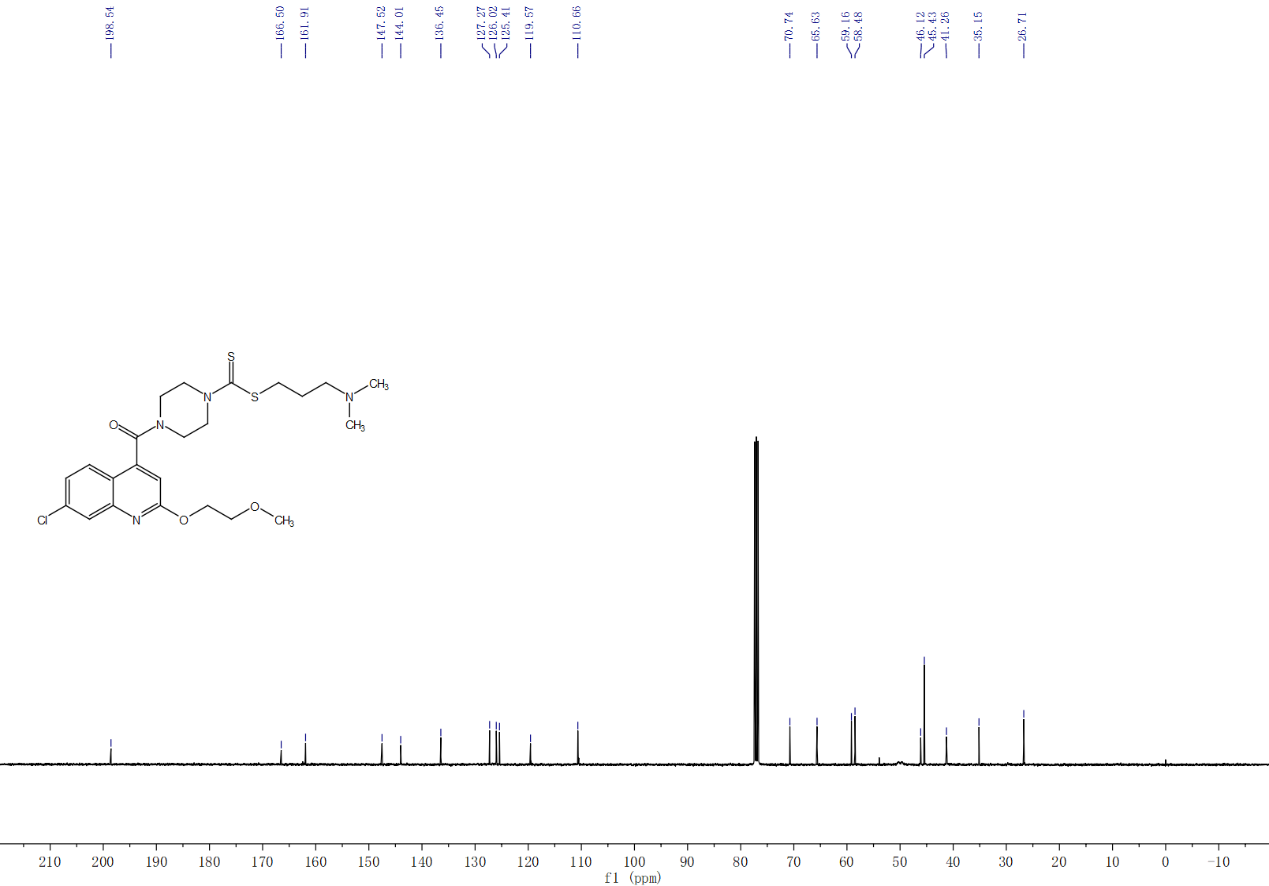


^13^C NMR of Compound **F28**

HRMS of Compound **F28**


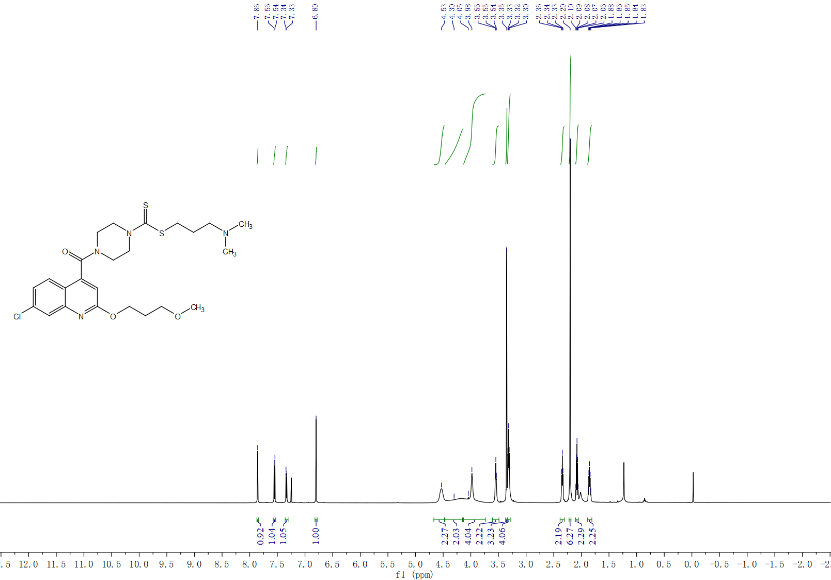


^1^H NMR of Compound **F29**


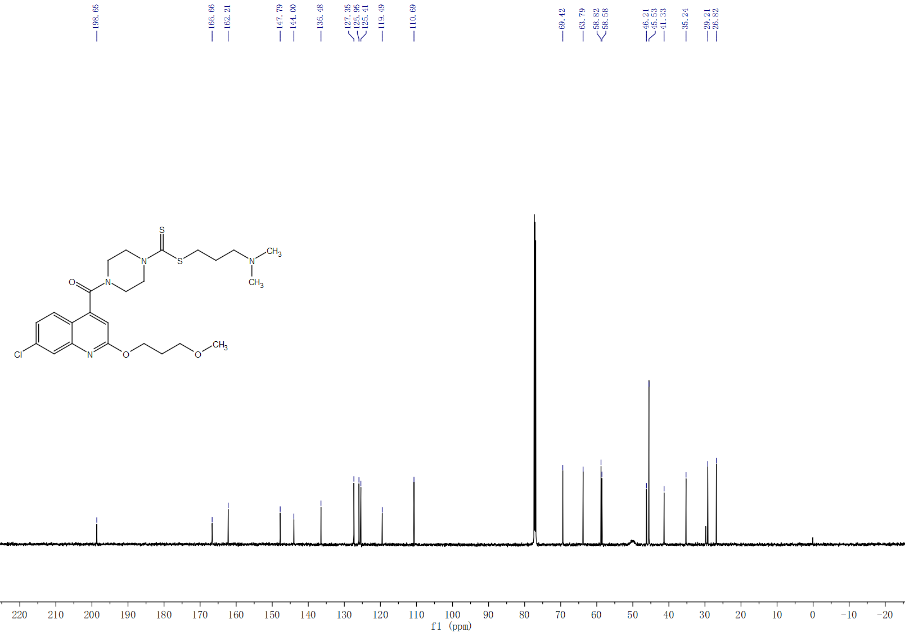


^13^C NMR of Compound **F29**

HRMS of Compound **F29**


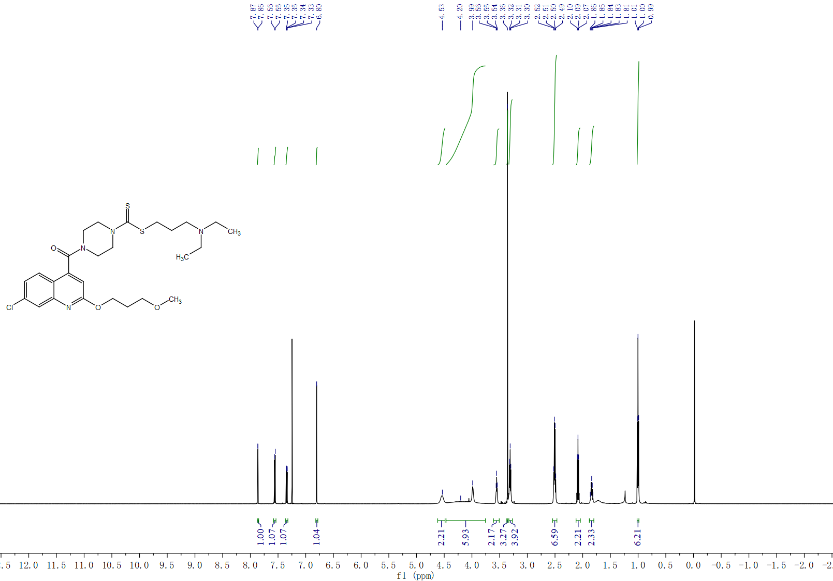


^1^H NMR of Compound **F30**


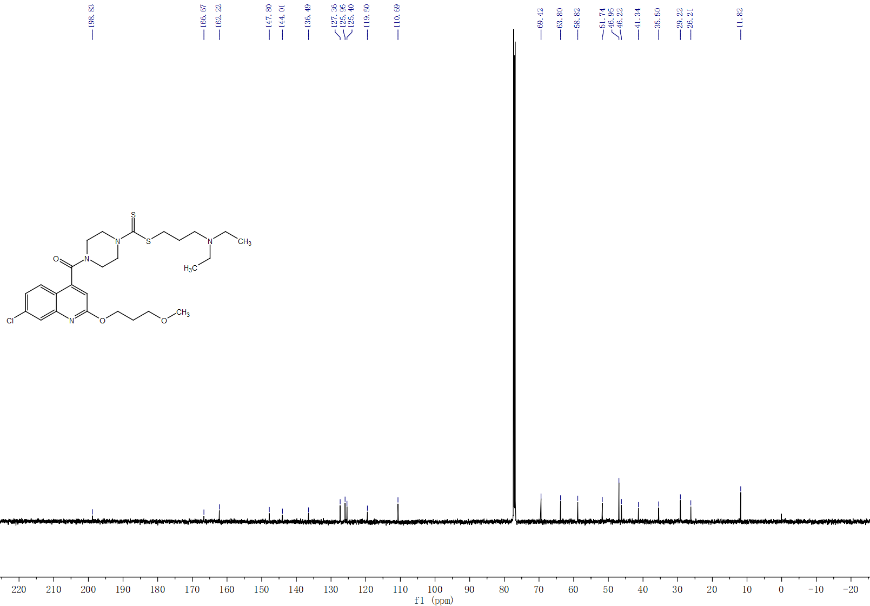


^13^C NMR of Compound **F30**

HRMS of Compound **F30**


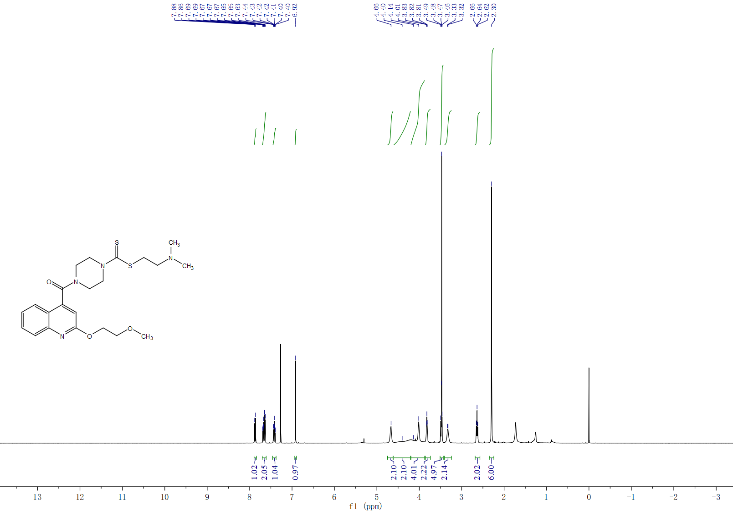


^1^H NMR of Compound **F31**


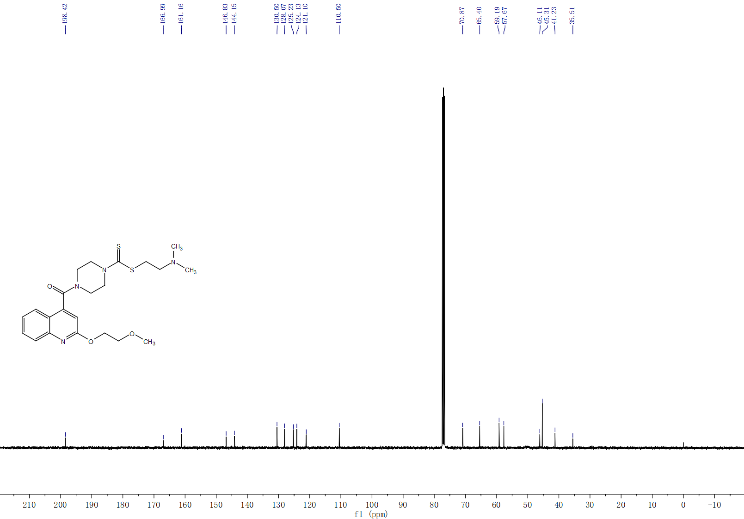


^13^C NMR of Compound **F31**

HRMS of Compound **F31**


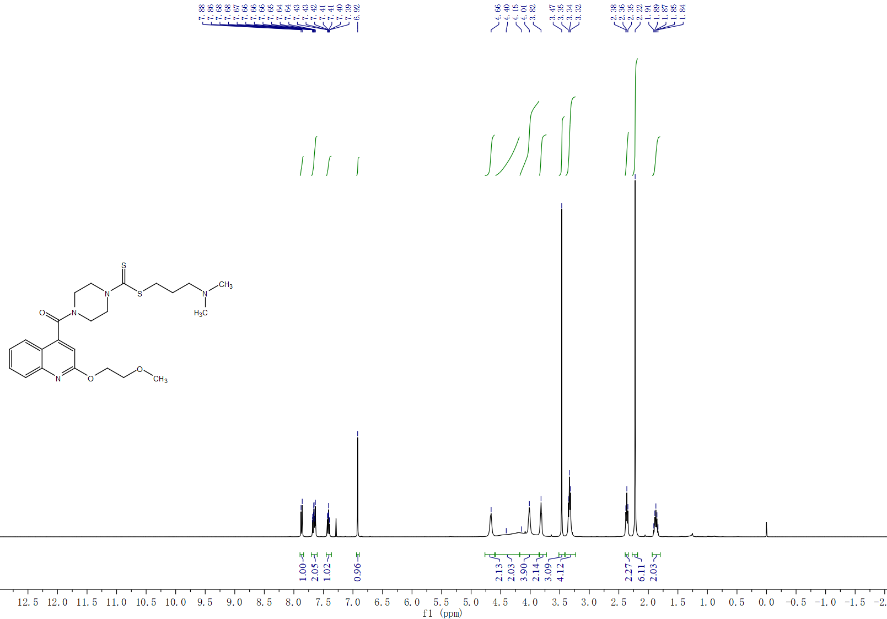


^1^H NMR of Compound **F32**


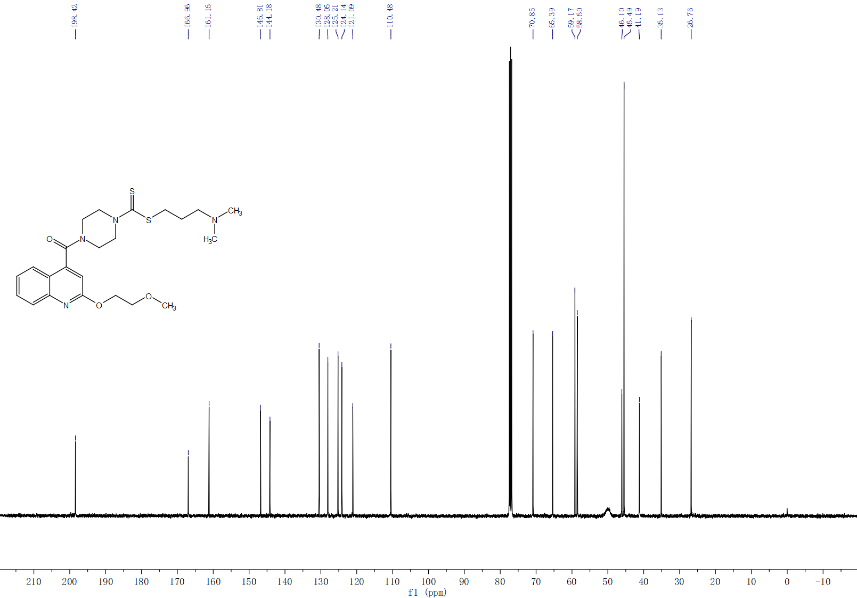


^13^C NMR of Compound **F32**

HRMS of Compound **F32**


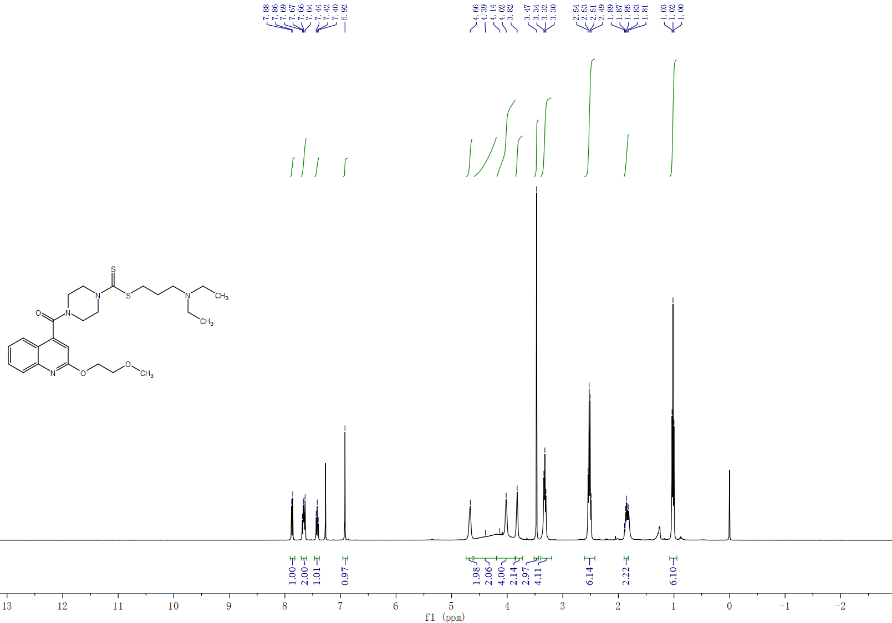


^1^H NMR of Compound **F33**


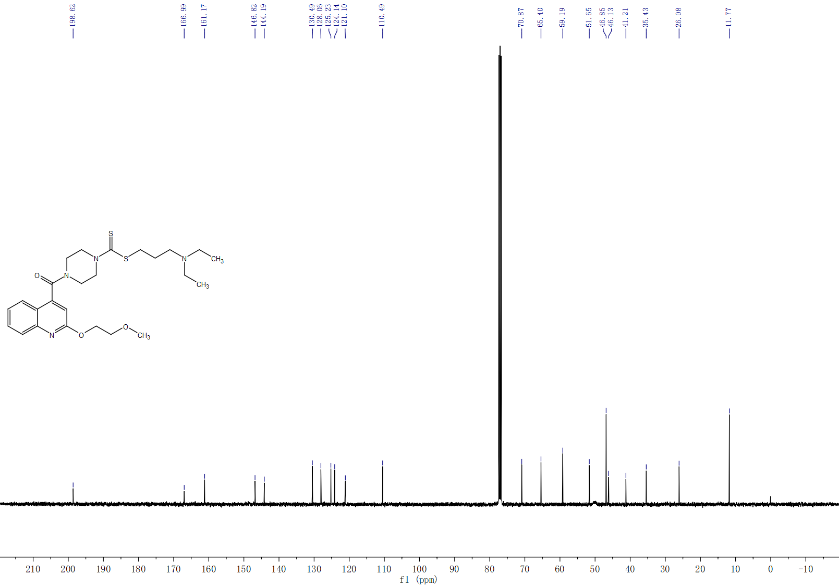


^13^C NMR of Compound **F33**

HRMS of Compound **F33**


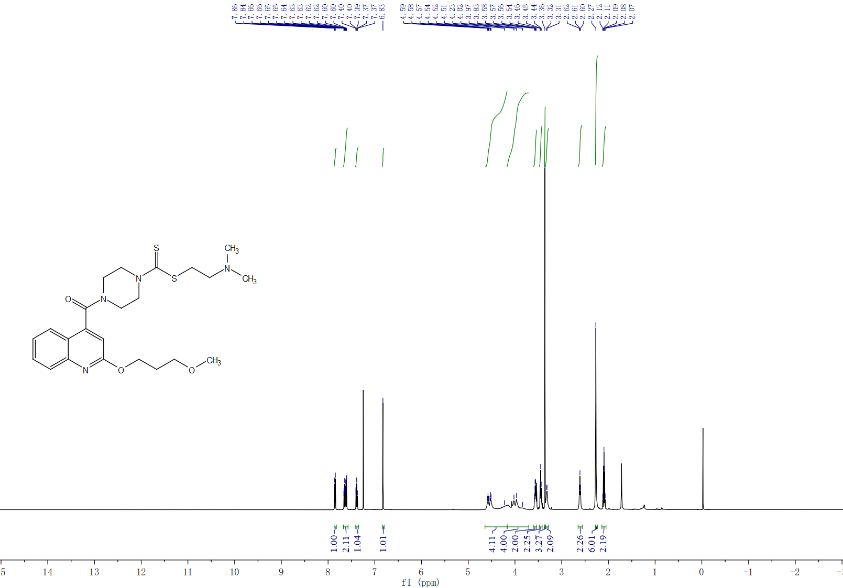


^1^H NMR of Compound **F34**


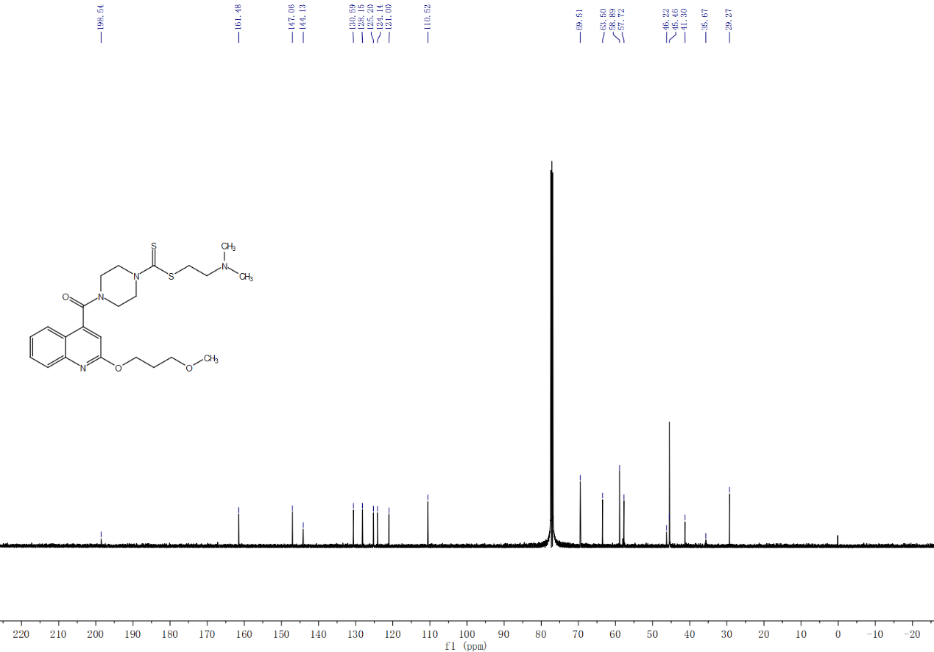


^13^C NMR of Compound **F34**

HRMS of Compound **F34**


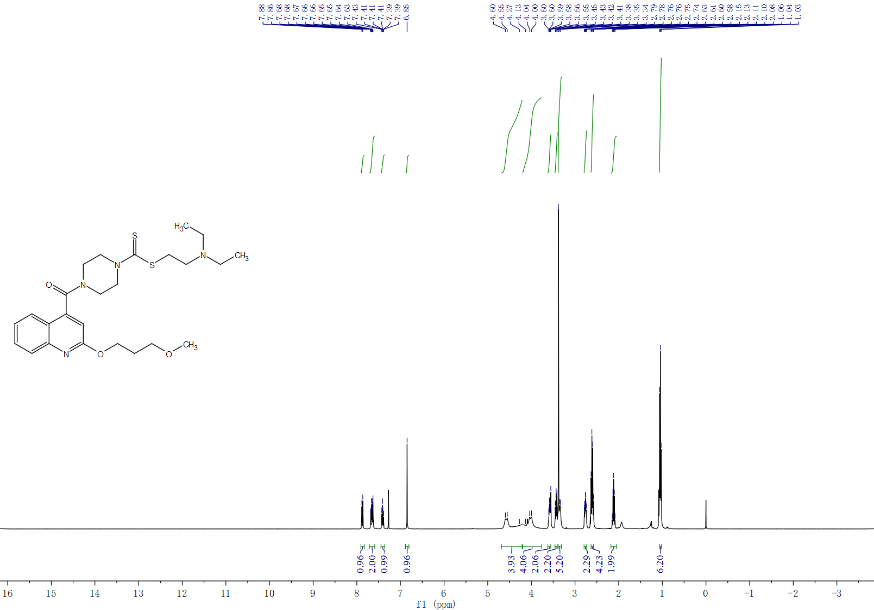


^1^H NMR of Compound **F35**


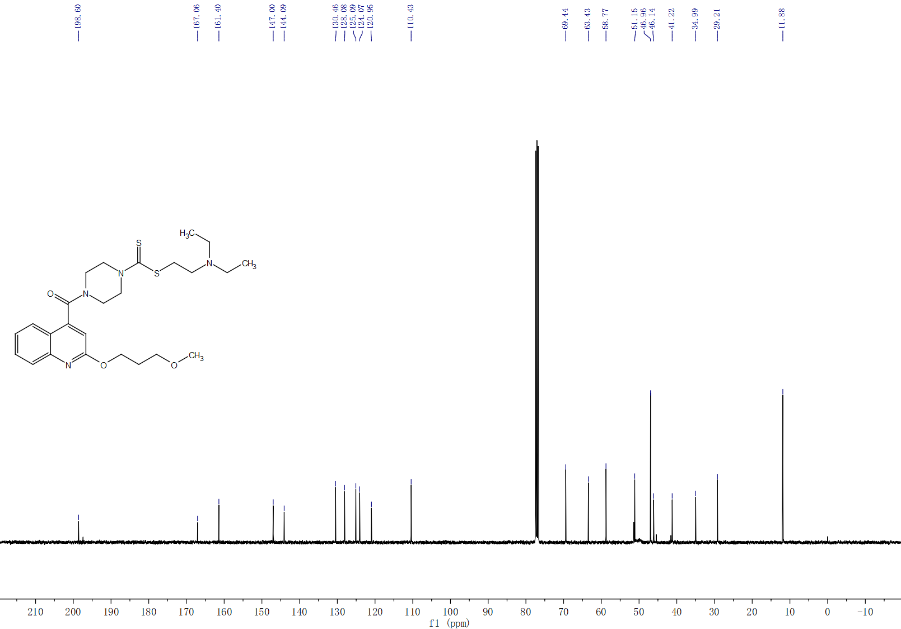


^13^C NMR of Compound **F35**

HRMS of Compound **F35**


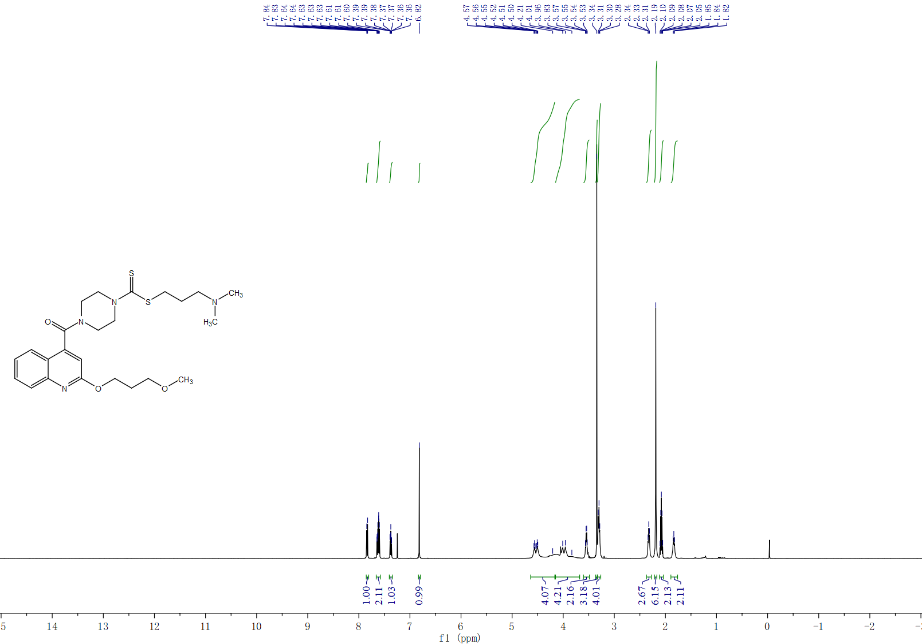


^1^H NMR of Compound **F36**


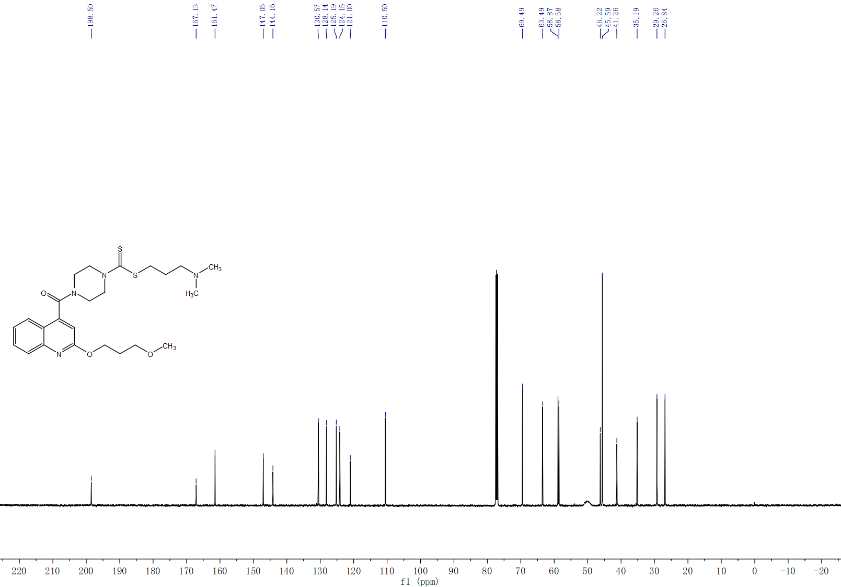


^13^C NMR of Compound **F36**

HRMS of Compound **F36**


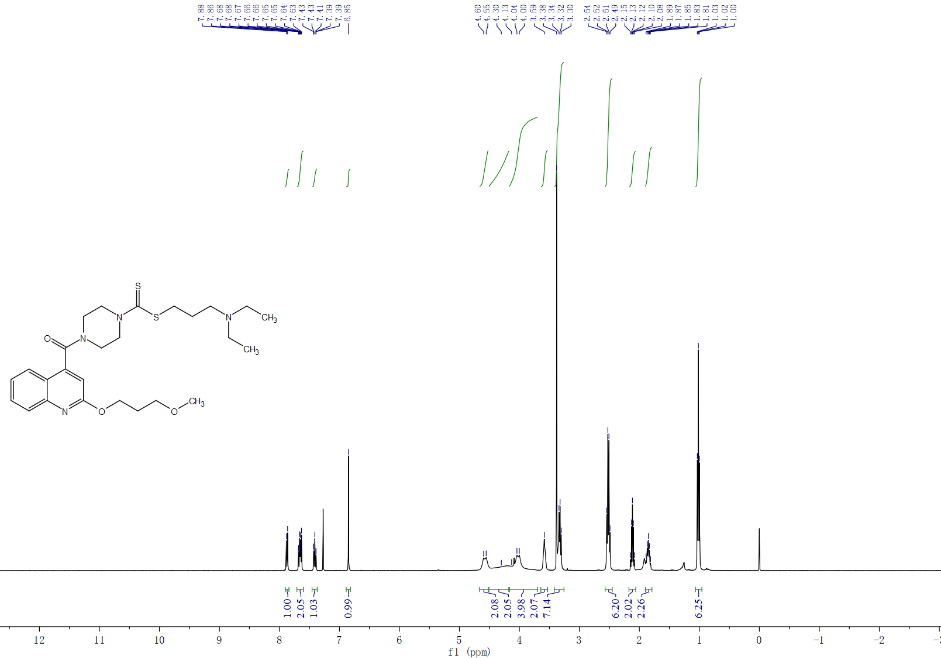


^1^H NMR of Compound **F37**


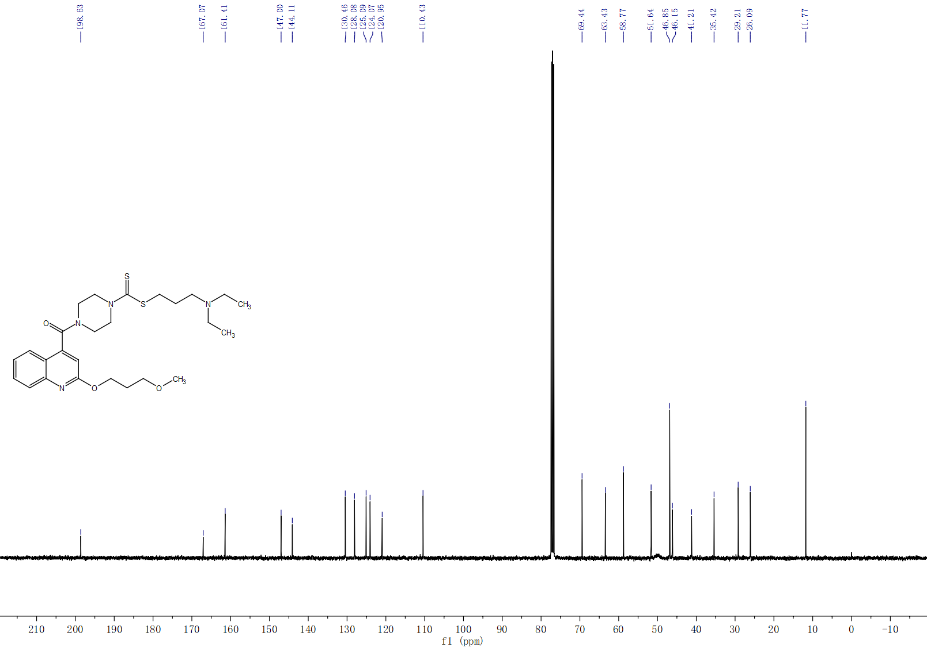


^13^C NMR of Compound **F37**

HRMS of Compound **F37**

**6. References**

Li, L.J., Liu, Z.B., Tang, S.Y., Li, J., Ren, X.H., Yang, G.Y., Li, H. *et al.* (2019) Amphiphilic ligands for Cu-catalyzed aerobic oxidation to synthesize 9-fluorenones in water. *Catal. Commun.* **127,** 34-38.

Li, W.L., Xu, F.J., Shuai, W., Sun, H.H., Yao, H., Ma, C., Xu, S.T. *et al.* (2018) Discovery of novel quinoline-chalcone derivatives as potent antitumor agents with microtubule polymerization inhibitory activity. *J. Med. Chem.* **62,** 993-1013.

Su, Y., Li, R.D., Ning, X.L., Lin, Z.Q., Zhao, X.Y., Zhou, J.T., Liu, J. *et al.* (2019) Discovery of 2,4-diarylaminopyrimidine derivatives bearing dithiocarbamate moiety as novel FAK inhibitors with antitumor and anti-angiogenesis activities. *Eur. J. Med. Chem.* **177,** 32-46.

Wang, L.X., Zhou, X.B., Xiao, M.L., Jiang, N., Liu, F., Zhou, W.X., Wang, X.K. *et al.* (2014) Synthesis and biological evaluation of substituted 4-(thiophen-2-ylmethyl)-2H-phthalazin-1-ones as potent PARP-1 inhibitors. *Bioorg. Med. Chem. Lett.* **24,** 3739-3743.

Jo, S., Kim, T., Iyer, V.G. and Im, W. (2008) CHARMM-GUI: A Web-based Graphical User Interface for CHARMM. *J. Comput. Chem.* **29***.* 1859-1865.

Brooks, B.R., Brooks, C.L., Mackerell, A.D., Nilsson, L., Petrella, R.J., Roux, B., Won, Y. *et al.* (2009) CHARMM: The biomolecular simulation program. *J. Comput. Chem.* **30**, 1545-1614.

Supplementary Movie 1. The fusion of IBs resulting from the interaction between TSWV N^WT^ and NbPGK in BiFC assays was observed by confocal microscopy at 48 hpi.

Supplementary Movie 2. The fission of IBs resulting from the interaction between TSWV N^WT^ and NbPGK in BiFC assays was observed by confocal microscopy at 48 hpi.

Supplementary Movie 3. FRAP analysis of condensates formed by the interaction between TSWV N^WT^ and NbPGK in BiFC assays.

Supplementary Movie 4. FRAP analysis of the droplets formed by sfGFP-N^WT^ and sfGFP-NbPGK *in vitro*.

Supplementary Movie 5. Fluorescence time-lapse confocal images showing the fusion of IBs formed by N-YFP in *N. benthamiana* cells at 48 hpi.

Supplementary Movie 6. Fluorescence time-lapse confocal images showing the fission of IBs formed by N-YFP in *N. benthamiana* cells at 48 hpi.

Supplementary Movie 7. FRAP analysis of condensates formed by N-YFP.
